# Rodent Ectoparasites in the Middle East: A Systematic Review and Meta-Analysis

**DOI:** 10.3390/pathogens10020139

**Published:** 2021-01-31

**Authors:** Md Mazharul Islam, Elmoubashar Farag, Khalid Eltom, Mohammad Mahmudul Hassan, Devendra Bansal, Francis Schaffner, Jolyon M. Medlock, Hamad Al-Romaihi, Zilungile Mkhize-Kwitshana

**Affiliations:** 1 Department of Animal Resources, Ministry of Municipality and Environment, Doha, P.O. Box 35081, Qatar; 2 School of Laboratory Medicine and Medical Sciences, College of Health Sciences, University of KwaZulu Natal, Durban 4000, South Africa; 3 Ministry of Public Health, Doha, P.O. Box 42, Qatar; dbansal@moph.gov.qa (D.B.); halromaihi@moph.gov.qa (H.A.-R.); 4 Department of Virology, The Central Laboratory, Ministry of Higher Education and Scientific Research, Khartoum, Khartoum 7099, Sudan; eltom.k.m.a@gmail.com; 5 Faculty of Veterinary Medicine, Chattogram Veterinary and Animal Sciences University, Khulshi, Chattogram 4225, Bangladesh; miladhasan@yahoo.com; 6 Francis Schaffner Consultancy, 4125 Riehen, Switzerland; fschaffner.consult@gmail.com; 7 Medical Entomology and Zoonoses Ecology, Public Health England, Salisbury SP4 0JG, UK; Jolyon.Medlock@phe.gov.uk; 8 School of Life Sciences, College of Agriculture, Engineering & Science, University of KwaZulu Natal, Durban 40000, South Africa; mkhizekwitshanaz@ukzn.ac.za; 9 South African Medical Research Council, Cape Town 7505, South Africa

**Keywords:** rodents, ectoparasites, fleas, lice, mites, ticks, Middle East, systematic review, meta-analysis

## Abstract

Rodents carry many ectoparasites, such as ticks, lice, fleas, and mites, which have potential public health importance. Middle Eastern countries are hotspots for many emerging and re-emerging infectious diseases, such as plague, leishmaniasis, Crimean Congo hemorrhagic fever, and Q fever, due to their ecological, socioeconomic, and political diversity. Rodent ectoparasites can act as vectors for many of these pathogens. Knowledge of rodent ectoparasites is of prime importance in controlling rodent ectoparasite-borne zoonotic diseases in this region. The current systematic review and meta-analysis performs a comprehensive synthesis of the available knowledge, providing an evidence-based overview of the ectoparasites detected on rodents in Middle Eastern countries. Following a systematic search in Pubmed, Scopus, and Web of Science, a total of 113 published articles on rodent ectoparasites were studied and analyzed. A total of 87 rodent species were documented, from which *Mus musculus*, *Rattus norvegicus*, and *Rattus rattus* were found to be the most common. Fleas were the most reported ectoparasites (87 articles), followed by mites (53), ticks (44), and lice (25). *Xenopsylla cheopis*, *Polyplax spinulosa*, *Ornithonyssus bacoti*, and *Hyalomma rhipicephaloides* were the most commonly described fleas, lice, mites, and ticks, respectively. Based on the reviewed articles, the median flea, louse, mite, and tick indices were highest in Israel (4.15), Egypt (1.39), Egypt (1.27), and Saudi Arabia (1.17), respectively. Quantitative meta-analysis, using a random-effects model, determined the overall pooled flea prevalence in the Middle East as 40% (95% CI: 25–55, *I*^2^ = 100%, *p* < 0.00001), ranging between 13% (95% CI: 0–30, *I*^2^ = 95%, *p* < 0.00001) in Iran and 59% (95% CI: 42–77, *I*^2^ = 75%, *p* < 0.00001) in Israel. The overall pooled louse prevalence was found to be 30% (95% CI: 13–47, *I*^2^ = 100%, *p* < 0.00001), ranging between 25% in Iran (95% CI: 1–50, *I*^2^ = 99%) and 38% in Egypt (95% CI: 7–68, *I*^2^ = 100%). In the case of mites, the pooled prevalence in this region was 33% (95% CI: 11–55, *I*^2^ = 100%, *p* < 0.00001), where the country-specific prevalence estimates were 30% in Iran (95% CI: 4–56, *I*^2^ = 99%) and 32% in Egypt (95% CI: 0–76, *I*^2^ = 100%). For ticks, the overall prevalence was found to be 25% (95% CI: 2–47, *I*^2^ = 100%, *p* < 0.00001), ranging from 16% in Iran (95% CI: 7–25, *I*^2^ = 74%) to 42% in Egypt (95% CI: 1–85, *I*^2^ = 100%). The control of rodent ectoparasites should be considered to reduce their adverse effects. Using the One Health strategy, rodent control, and precisely control of the most common rodent species, i.e., *Mus musculus*, *Rattus norvegicus*, and *Rattus rattus*, should be considered to control the rodent-borne ectoparasites in this region.

## 1. Introduction

Ectoparasites are organisms that infest the exterior surface, such as skin or its integument, of a host [1,2]. The vast majority of human and animal ectoparasites are arthropods. Ectoparasites can cause multiple health problems for the host, such as anemia, hypersensitivity, irritability, and skin lesions [2]. They also act as vectors of many pathogens of public and animal health importance, such as *Crimean-Congo hemorrhagic fever virus* (CCHFV), *Coxiella*, *Rickettsia* and *Hymenolepis* [3,4,5,6]. 

Rodents are the largest and most diverse group of animals among mammals in the world [7]. These animals are one of the major causes of crop and resource damage worldwide [8]. Moreover, after bats, rodents have the highest importance for carrying zoonotic pathogens [9]. Since the middle ages, rodents have contributed to the spread of many disease pandemics, such as plague, murine typhus, and leishmaniasis. Rodents carry different ectoparasites, which act as vectors of these pathogens [10]. There are many other zoonotic pathogens, such as *Hymenolepis diminuta*, *Bartonella* sp., *Coxiella burnetii*, and *Rickettsia* sp., which have been identified from rodent-borne fleas, mites, and ticks [4,11,12]. Rodents carry many ectoparasites, such as lice, fleas, ticks, and mites [10], that are associated with low socioeconomic status, war, famine, climatic events (e.g., floods), and environmental changes, facilitating the transmission of pathogens among the human and animal populations [13,14,15]. 

The Middle East is centered on Afro-Eurasia, and includes member countries of the Gulf Cooperation Council (Bahrain, Kuwait, Oman, Qatar, Saudi Arabia, and United Arab Emirates (UAE)), in addition to Cyprus, Iran, Iraq, Israel, Jordan, Lebanon, Palestine, Syria, Turkey, and Yemen [16,17]. Countries in the Middle East are hotspots for emerging and re-emerging infectious diseases, partly because of their ecological, cultural, socioeconomic, and political diversity, but also due to the unrest, conflict, and wars in this region [18,19]. The lack of relevant information on infectious diseases, their sources, and their diversity is a major drawback for public health studies in this area, possibly misguiding both civilians and governments in their attempts at mitigation [20]. 

In the past, the Middle East experienced several rodent ectoparasite-associated disease epidemics that caused the loss of millions of lives, such as plague and murine typhus [21,22,23]. Even today, many Middle Eastern countries remain at risk of particular rodent ectoparasite-associated infectious diseases, such as leishmaniasis [24]. As such, it is of the utmost importance for regional health authorities to control the spread of rodents and their ectoparasites, and fully-characterize their ecological niche and diversity. To date, several studies have been undertaken on rodent ectoparasites and related diseases in this region. However, to the best of our knowledge, this is the first systematic review that aims to summarize, analyze and interpret the available baseline data to provide an in-depth understanding of the presence and abundance of rodent ectoparasites in this region.

## 2. Methods

**Figure 1:**
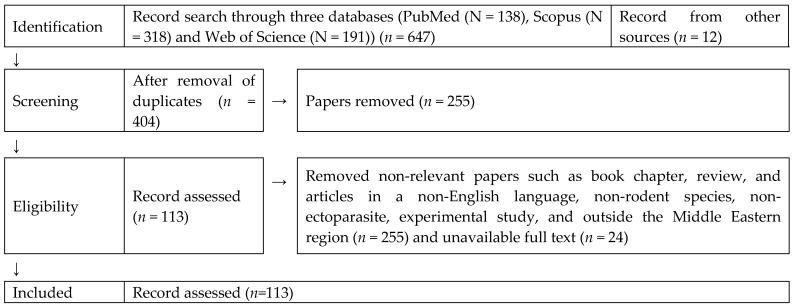
Systematic review preferred reporting items for systematic reviews and meta-analysis (PRISMA) flow diagram describing the selection of published articles on rodent ectoparasites in the Middle East and the inclusion/exclusion process used in the study.

This systematic review was conducted in full accordance with the preferred reporting items for systematic reviews and meta-analysis (PRISMA) guidelines (Figure 1 and Supplementary Table S1) [25]. One author performed the search in electronic databases, two authors cross-examined the titles, abstracts, and full-texts of the retrieved citations against a set of predetermined selection criteria, and then one author compiled the relevant data. Subsequently, three authors organized the data and conducted the meta-analysis. The review protocol was registered in Open Science Framework (OSF) Registries under the following DOI: 10.17605/OSF.IO/RPYK8.

### 2.1. Search Strategy

Systematic searches on PubMed, Scopus, and Web of Science were performed by 16 October 2020. The search covered every original research article published in English containing field information on rodent ectoparasites in the Middle Eastern countries without any restrictions on publication dates. Following a previous systematic review [26], the keywords included (Rodent OR Rat OR Jird OR Gerbil OR Vole OR Mouse OR Hamster OR Porcupine OR Squirrel OR Jerboa) AND (Ectoparasite OR Flea OR Mite OR Lice OR Tick) AND (17 Middle Eastern countries name linked with OR). We used advanced search strategies, i.e., [Title/Abstract] in PubMed, [TITLE-ABS-KEY] in Scopus, and [Topic] in Web of Science, to screen the searches.

### 2.2. Search of Relevant Articles

At first, EndNote X9 (Clarivate Analytics, Philadelphia, PA, USA) was used to identify and exclude duplicate studies. Imported citations were then transferred to Rayyan (https://rayyan.qcri.org/) for title and abstract screening. If any article’s title and abstract were ambiguous in terms of relevance to our study, it was subjected to full-text analysis. 

### 2.3. Quality Assessment of the Selected Articles

The quality assessment of all included articles was conducted using a modified version of the critical appraisal tool for prevalence studies created by the Joanna Briggs Institute and reported by Munn et al. [27]. A checklist with 10 questions was used (Supplementary Table S2) to assess the risk of confounding bias, selection bias, and bias related to measurement and data analysis. Each question was answered either with “yes”, “no”, “unclear” or “not/applicable”. A score was calculated as the number of questions answered with a “yes” for each study. According to this score, studies were categorized into three groups based on their quality: low (a score of 0–4), intermediate (5–6), and high quality (7–10). Representative samples were those with basic characteristics that mimic our targeted population (rodents and ectoparasites) selected through the fieldwork. For practical reasons, the adequate sample size for each study was estimated in a case-by-case manner, taking into account the geographical area it represents, study type, and the rodent species in question. The sampling location and other details of the setting of fieldwork had to be described appropriately. Studies had to explain how they identified different rodents and ectoparasite species in detail, or use valid references of identification methods. Additionally, articles had to explicitly report the calculations of ectoparasite indices and prevalence, or provide enough baseline data for the reviewers to calculate these measures on their behalf. The appropriateness of statistical analysis was evaluated in relation to the objectives of each study. Important subgrouping was expected according to the type and species of rodents and ectoparasites. 

### 2.4. Data Extraction

We considered only the field reports on rodent ectoparasites for data extraction. The extracted variables were the country and year of sampling, rodent-specific data (species, gender, total rodent count, and the number of ectoparasite-infected rodents), ectoparasite-specific data (type, species, and total number), and the associating factors for ectoparasite abundance on rodents (Supplementary Table S3). The taxonomy of all reported rodents and ectoparasites were verified through online databases, namely the National Center for Biotechnology Information (NCBI) Taxonomy Browser, the Global Biodiversity Information Facility (GBIF), Animal Diversity Web (ADW), and the Zoological Institute of Russian Academy of Sciences.

### 2.5. Data Analysis

The extracted data were organized and stored in Microsoft Excel (MS Office, 2019) spreadsheets. The initial descriptive analysis of the included studies was conducted using the same application. Ectoparasite indices were calculated for each of the four types of ectoparasites (fleas, lice, mites, and ticks) by dividing the total numbers detected for the specific ectoparasite by the total number of sampled rodents [28]. Central tendency and dispersion were calculated for country-specific ectoparasite indices and illustrated in Boxplots using the BoxplotR web tool [29]. An ectoparasite’s prevalence was calculated by dividing the total number of ectoparasite-positive rodents over the total number of sampled rodents, and was expressed in decimals. Quantitative meta-analysis was conducted by one co-author (K.E.) using Review Manager 5.3 (The Nordic Cochrane Centre, Cochrane Collaboration, Copenhagen, Denmark), and the results were verified by another co-author (MMH) using STATA/IC-13.0 (Stata Corp, 4905 Lakeway Drive, College Station, Texas 77845, USA). In both instances, a random-effects model was applied to calculate the pooled prevalence of all types of ectoparasites with 95% confidence intervals (CI). Studies were weighted according to the inverse of variance. The prevalence reported by each study was used as the effect estimate, and its standard error (SE) was calculated using the formula *SE* = *SQRT*(*p*(1 − *p*)/*n*), where *p* is the reported prevalence, and *n* is its sample size. The Inconsistency Index (*I*^2^) was used to assess the degree of heterogeneity among studies, as it is known to be less influenced by the number of included studies. According to the country and rodent species, subgroup meta-analyses were performed to investigate possible explanations of significant heterogeneity (*I*^2^ > 75%). However, each subgroup had to be represented by at least three studies to be included for analysis. The results of all meta-analyses were illustrated in forest plots. Finally, funnel plots were generated and visually-examined to assess the possibility of publication bias.

## 3. Results

### 3.1. Descriptive Analysis

**Figure 2:**
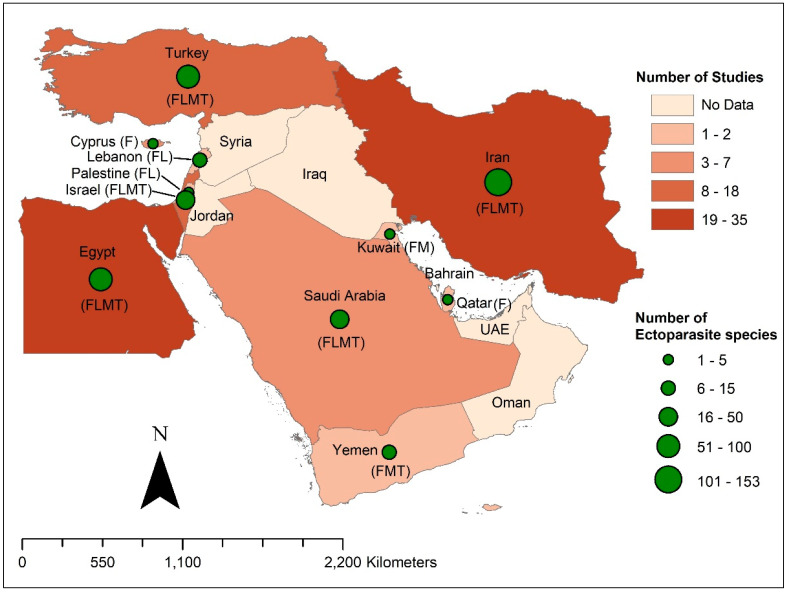
The map describes the Middle Eastern countries with the total number of studies and the number of ectoparasite species detected on rodents (the letters F, L, M, and T indicate information available about fleas, lice, mites, and ticks, respectively).

The literature search resulted in 113 articles (Figure 1) published from 1914 to 2020 [3,4,5,11,12,14,24,30,31,32,33,34,35,36,37,38,39,40,41,42,43,44,45,46,47,48,49,50,51,52,53,54,55,56,57,58,59,60,61,62,63,64,65,66,67,68,69,70,71,72,73,74,75,76,77,78,79,80,81,82,83,84,85,86,87,88,89,90,91,92,93,94,95,96,97,98,99,100,101,102,103,104,105,106,107,108,109,110,111,112,113,114,115,116,117,118,119,120,121,122,123,124,125,126,127,128,129,130,131,132,133,134,135]. The articles were covering 11 out of 17 Middle Eastern countries (Figure 2). However, no information was available from the countries Bahrain, Iraq, Jordan, Oman, Syria, or the UAE. Among the 113 published articles, 82 articles focused on rodent fleas, 38 on rodent lice, 53 on rodent mites, and 44 on rodent ticks. A total of 61 (54%) articles were of high quality, followed by 29 (26%) with intermediate quality, and 23 (20%) were low-quality articles (Supplementary Table S2). The visual examination of funnel plots revealed evidence of possible publication bias in all meta-analyses, as more articles were near the top, with an asymmetrical distribution on both sides of the overall pooled prevalence estimate (Supplementary Figure S1).

The 113 studies examined at least 26,003 rodents from 87 rodent species belonging to seven families (Supplementary Table S4a). Among these, *Mus musculus* (9% of total examined rodents), *Rattus norvegicus* (48%), and *Rattus rattus* (19%) were found to be the most common and widely-distributed rodents. Moreover, *Acomys cahirinus*, *Acomys dimidiatus*, *Apodemus mystacinus*, *Apodemus sylvaticus*, *Cricetulus migratorius*, *Gerbillus nanus*, *Jaculus jaculus*, *Meriones crassus*, *Meriones libycus*, and *Meriones tristrami* were reported from at least three countries of the Middle East, and can be considered as widely-distributed rodents in this region.

**Figure 3:**
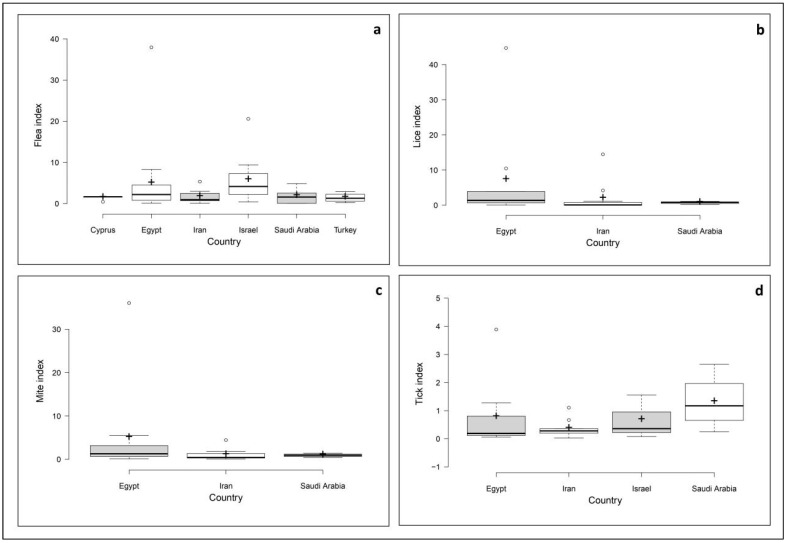
Ectoparasite indices in the Middle Eastern countries; (**a**) flea index, (**b**) louse index, (**c**) mite index, and (**d**) tick index. Centerlines indicate the medians; box limits indicate the 25 to 75 percentiles as determined by R software; whiskers extend the interquartile range 1.5-fold from the 25 to the 75 percentiles; outliers are represented by dots; crosses represent sample means.

Based on the reviewed articles, the Boxplots (Figure 3) summarize the results of the reported ectoparasite indices in some of the Middle Eastern countries. The median flea index was the highest in Israel (4.15) and lowest in Iran (0.95). In the case of louse, it ranged from a median of 0.09 in Iran to 1.39 in Egypt. The median mite index was 0.42 in Iran, 0.94 in Saudi Arabia, and 1.27 in Egypt, whereas the median tick indices in Middle Eastern countries were 0.19 in Egypt, 0.28 in Iran, 0.36 in Israel, and 1.17 in Saudi Arabia. 

### 3.2. Fleas Carried by Rodents in the Middle East

Based on the records of 82 articles with rodent fleas, a total of 67,057 fleas were examined, which were from 104 flea species (Supplementary Table S4b), of which most of the fleas were *Xenopsylla cheopis*, *Echidnophaga gallinacea*, and *Xenopsylla cleopatrae* (23.6%, 16.3%, and 14.9% of total fleas, respectively). The most frequently reported species of fleas were *Xenopsylla cheopis* (41 reports), *Leptopsylla segnis* (22), and *Ctenocephalides felis* (19). Fifteen species of fleas were reported from at least three countries, such as *Echidnophaga murina*, *Leptopsylla segnis*, *Leptopsylla taschenbergi*, *Nosopsyllus fasciatus*, *Nosopsyllus iranus*, *Parapulex chephrenis*, *Pulex irritans*, *Stenoponia tripectinata*, *Xenopsylla astia*, *Xenopsylla cheopis*, *Xenopsylla cleopatrae*, *Xenopsylla conformis*, *Xenopsylla nubica*, and *Xenopsylla ramesis*.

**Figure 4:**
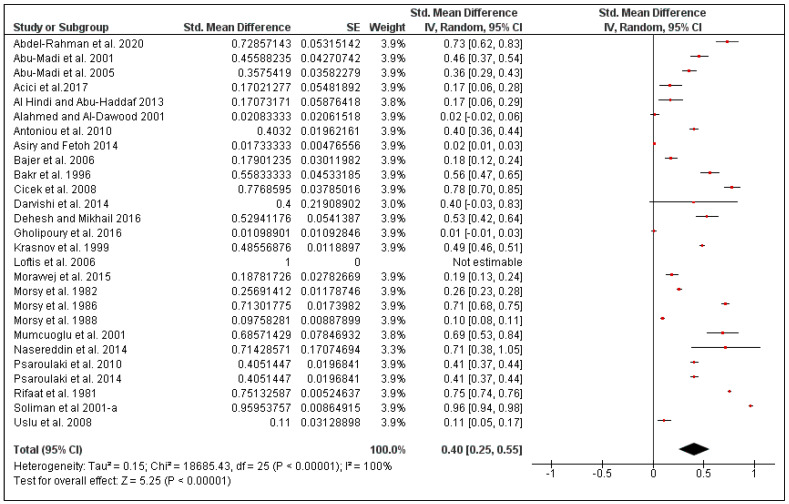
Forest plot of the pooled overall flea prevalence on rodents in the Middle Eastern countries. The central red square represents point estimates, whereas the square size represents the weight of each study in the meta-analysis.

**Figure 5:**
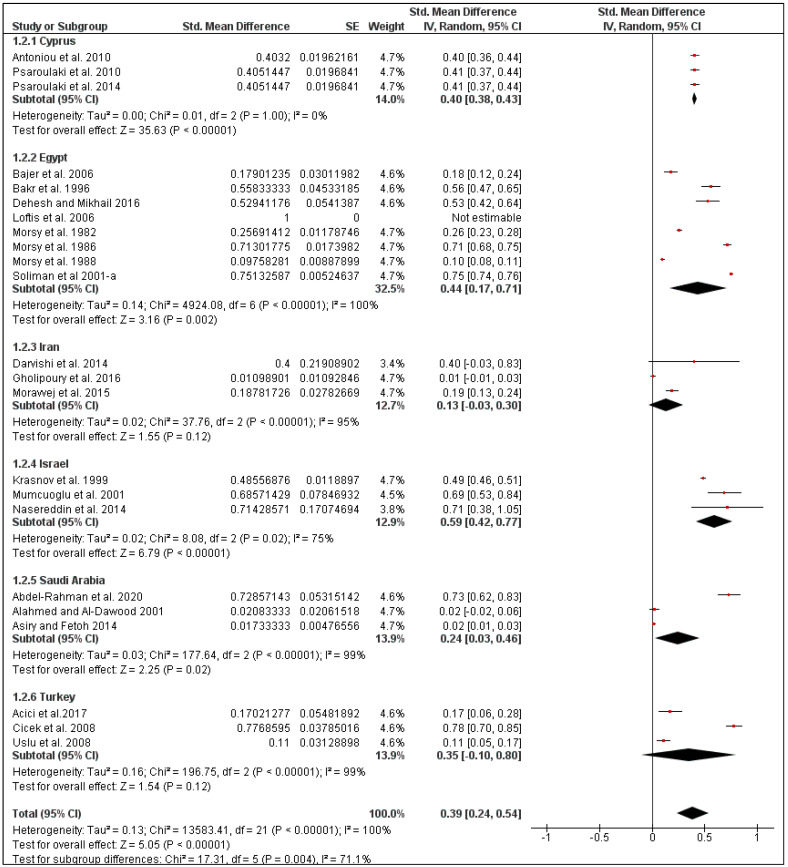
Forest plot illustrating subgroup meta-analysis of country-specific flea prevalence on rodents in the Middle Eastern countries. The central red square represents point estimates, whereas the square size represents the weight of each study in the meta-analysis.

**Figure 6:**
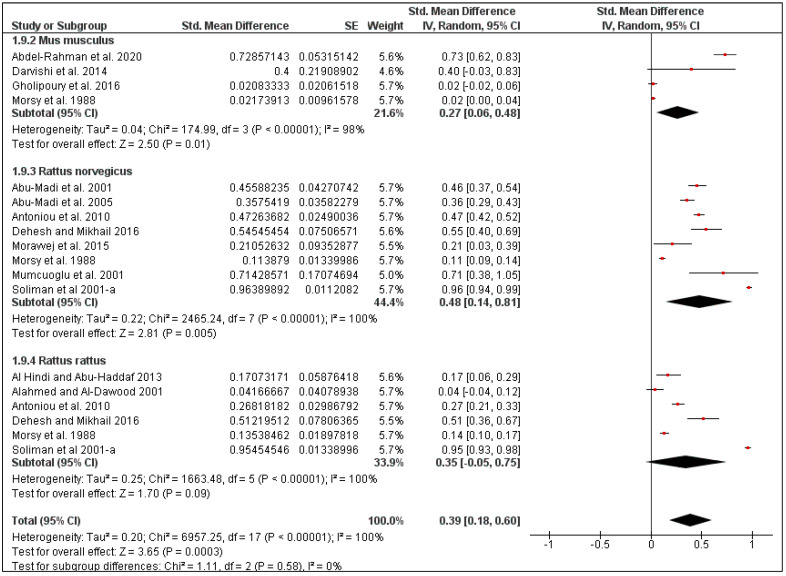
Forest plot illustrating subgroup meta-analysis of flea prevalence according to rodent species in Middle Eastern countries. The central red square represents point estimates, whereas the square size represents the weight of each study in the meta-analysis.

The overall pooled flea prevalence in the Middle East was found to be 40% (95% CI: 25–55, *I*^2^ = 100%, *p* < 0.00001), ranging between 13% (95% CI: 0–30, *I*^2^ = 95%, *p* < 0.00001) in Iran and 59% (95% CI: 42–77, *I*^2^ = 75%, *p* < 0.00001) in Israel (Figure 4 and Figure 5). Species-specific prevalence was calculated only for three rodent species: *Mus musculus* (27%, 95% CI: 6–48, *I*^2^ = 98%), *Rattus norvegicus* (48%, 95% CI: 14–81, *I*^2^ = 100%) and *Rattus rattus* (35%, 95% CI: 0–75, *I*^2^ = 100%) (Figure 6).

### 3.3. Lice Carried by Rodents in the Middle East

The 39 articles studied a collective 31,543 lice on rodents, and detected 28 species of lice in the Middle Eastern rodents (Supplementary Table S4c). However, *Polyplax spinulosa* represented 88.79% of the total lice, and was reported by 25 articles from Egypt, Iran, Kuwait, Palestine and Saudi Arabia. 

**Figure 7:**
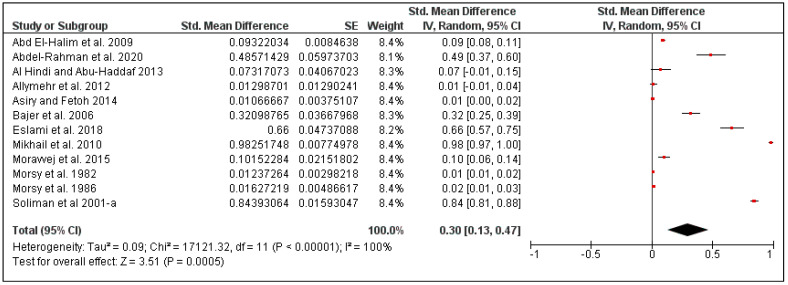
Forest plot of the pooled overall louse prevalence on rodents in the Middle Eastern countries. The central red square represents point estimates, whereas the square size represents the weight of each study in the meta-analysis.

**Figure 8:**
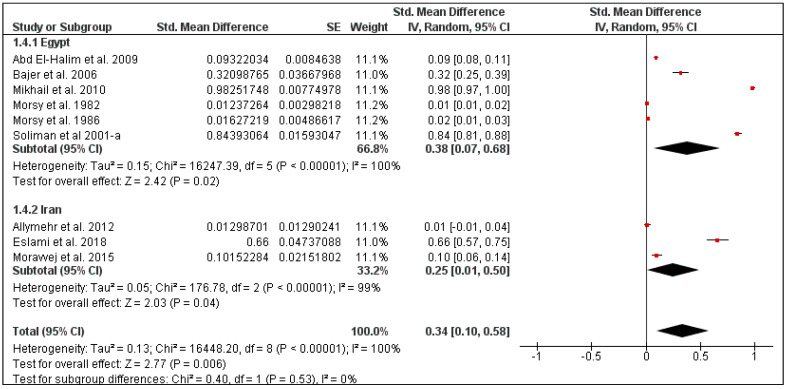
Forest plot illustrating subgroup meta-analysis of country-specific louse prevalence on rodents in the Middle Eastern countries. The central red square represents point estimates, whereas the square size represents the weight of each study in the meta-analysis.

**Figure 9:**
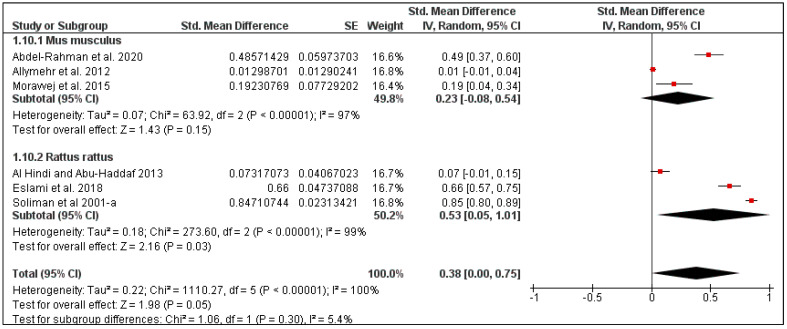
Forest plot illustrating subgroup meta-analysis of country-specific louse prevalence on rodents in Middle Eastern countries. The central red square represents point estimates, whereas the square size represents the weight of each study in the meta-analysis.

For rodents in this region, the overall pooled louse prevalence was 30% (95% CI: 13–47, *I*^2^ = 100%, *p* < 0.00001), ranging between 25% in Iran (95% CI: 1–50, *I*^2^ = 99%) and 38% in Egypt (95% CI: 7–68, *I*^2^ = 100%) (Figure 7 and Figure 8). Moreover, the louse prevalence was 23% in *Mus musculus* (95% CI: 7–68, *I*^2^ = 100%), and 53% in *Rattus rattus* (95% CI: 7–68, *I*^2^ = 100%) (Figure 9).

### 3.4. Mites Carried by Rodents in the Middle East

The review detected 134 species (Supplementary Table S4d) of mites (n = 26,476) on rodents in Middle Eastern countries, of which 73% were from three species, i.e., *Laelaps nuttalli* (29%), *Ornithonyssus bacoti* (34%), and *Radfordia ensifera* (10%). However, *Echinolaelaps echidninus*, *Eulaelaps stabularis*, *Haemolaelaps glasgowi*, *Laelaps nuttalli*, and *Ornithonyssus bacoti* were reported from at least three countries of the Middle East, whereas *Ornithonyssus bacoti* and *Laelaps nuttalli* were the highest reported mites (24 and 20 studies respectively out of 51 total studies on mites). 

**Figure 10:**
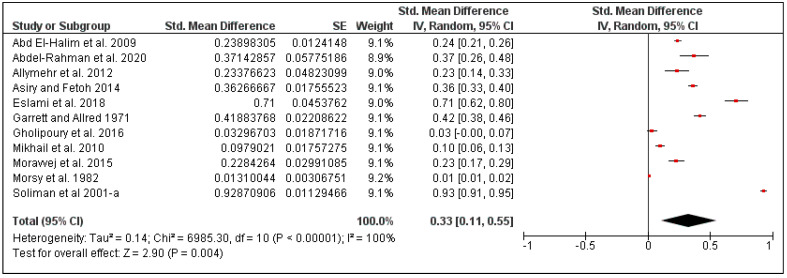
Forest plot of the pooled overall mite prevalence on rodents in the Middle Eastern countries. The central red square represents point estimates, whereas the square size represents the weight of each study in the meta-analysis.

**Figure 11:**
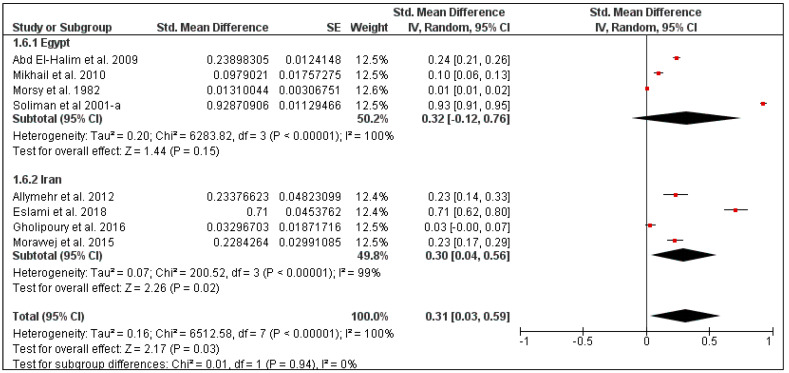
Forest plot illustrating subgroup meta-analysis of country-specific mite prevalence on rodents in the Middle Eastern countries. The central red square represents point estimates, whereas the square size represents the weight of each study in the meta-analysis.

**Figure 12:**
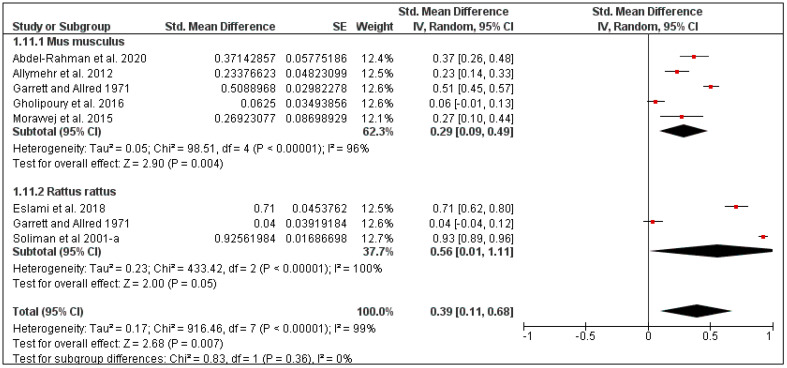
Forest plot illustrating subgroup meta-analysis of country-specific mite prevalence on rodents in the Middle Eastern countries. The central red square represents point estimates, whereas the square size represents the weight of each study in the meta-analysis.

The overall pooled mite prevalence in the Middle East was 33% (95% CI: 11–55, *I*^2^ = 100%, *p* < 0.00001) (Figure 10). Country-specific prevalence was calculated for Iran (30%, 95% CI: 4–56, *I*^2^ = 99%) and Egypt (32%, 95% CI: 0–76, *I*^2^ = 100%) (Figure 11). The prevalence also varied according to rodent species, from 29% in *Mus musculus* (95% CI: 9–49, *I*^2^ = 96%) to 56% in *Rattus rattus* (95% CI: 1–100, *I*^2^ = 100%) (Figure 12). 

### 3.5. Ticks Carried by Rodents in the Middle East

The reviewed studies identified 2897 ticks from at least 27 species (Supplementary Table S4e), of which 69.7% and 15.7% were *Hyalomma rhipicephaloides* and *Ixodes eldaricus*, respectively. Three species of ticks were reported from more than three countries, such as *Ixodes* spp., *Rhipicephalus sanguineus*, and *Rhipicephalus turanicus*. 

**Figure 13:**
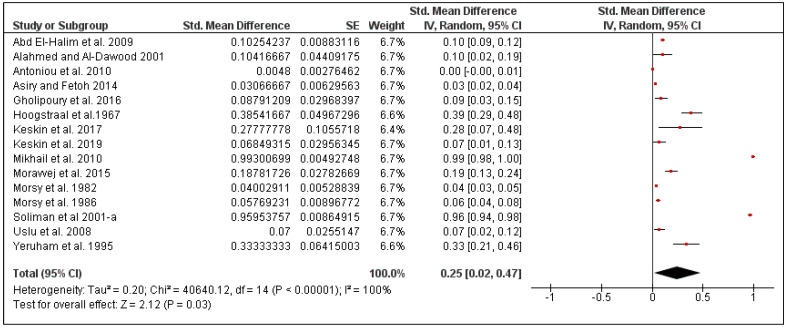
Forest plot of the pooled overall tick prevalence on rodents in Middle Eastern countries. The central red square represents point estimates, whereas the square size represents the weight of each study in the meta-analysis.

**Figure 14:**
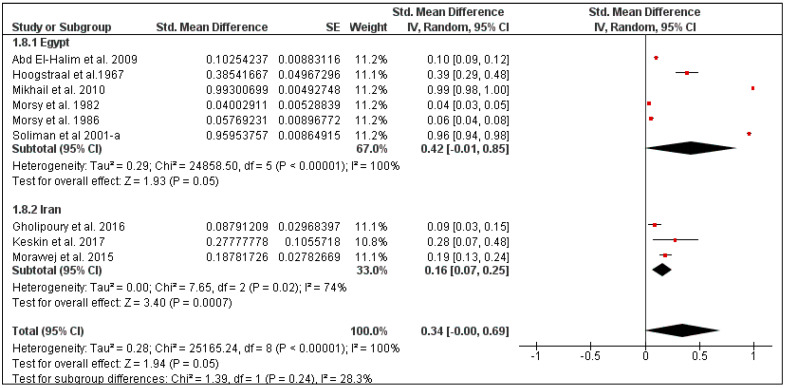
Forest plot illustrating subgroup meta-analysis of country-specific tick prevalence on rodents in Middle Eastern countries. The central red square represents point estimates, whereas the square size represents the weight of each study in the meta-analysis.

**Figure 15:**
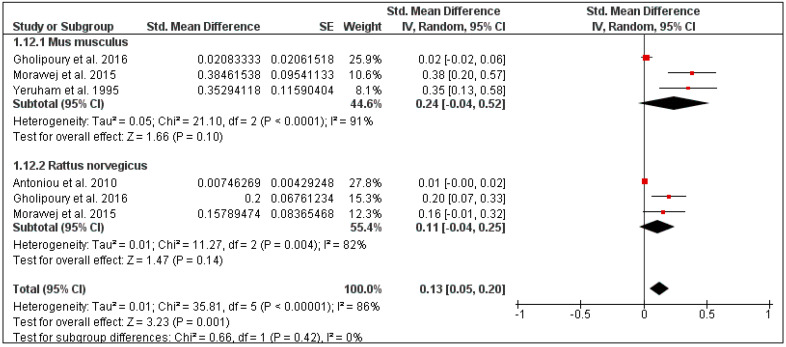
Forest plot illustrating subgroup meta-analysis of country-specific tick prevalence on rodents in the Middle Eastern countries. The central red square represents point estimates, whereas the square size represents the weight of each study in the meta-analysis.

The overall tick prevalence in this region was 25% (95% CI: 2–47, *I*^2^ = 100%, *p* < 0.00001) (Figure 13), ranging from 16% in Iran (95% CI: 7–25, *I*^2^ = 74%) to 42% in Egypt (95% CI: 1–85, *I*^2^ = 100%) (Figure 14). The tick prevalence also varied according to rodent species, from 11% in *Rattus norvegicus* (95% CI: 0–25, *I*^2^ = 82%), to 24% in *Mus musculus* (95% CI: 0–52, *I*^2^ = 91%) (Figure 15). 

## 4. Discussion

Our study reviewed the published literature on rodent ectoparasites in the Middle Eastern countries to provide a comprehensive overview of rodent ectoparasites in this region. Most of the studies were from Iran, Egypt, and Israel (82 out of 113). A previous history of rodent-borne disease epidemics, such as plague, leishmaniasis, and murine typhus, may be behind the increased interest in rodent-related pathogens by researchers in these countries [26]. Ectoparasite index and prevalence are suitable descriptors to quantify parasites in a host or estimate ectoparasite abundance [136,137]. These indices are essential to use in conjunction with rodent and vector surveillance to estimate human and epizootic risks [28]. However, the current review failed to calculate the pooled abundance of most Middle Eastern countries, possibly affecting the generalizability of our results and emphasizing the need for further detailed studies to understand the rodent ectoparasite abundance in this region, the resultant threat to the local population, and the necessary control measures. 

Although there were no rodent ectoparasite reports from Bahrain, Iraq, Jordan, Oman, Syria, and UAE in our systematic review, there are rodent-related ectoparasites reported in some of these countries from non-rodent hosts. The brown dog tick, *Rhipicephalus sanguineus*, is abundant on stray dogs in Jordan [138]. *Rhipicephalus sanguineus* and *Xenopsylla astia* were identified on domestic cats in UAE [139]. This indicates that there is a considerable gap in the knowledge in these countries where rodent-borne zoonoses are concerned. A previous review reported a knowledge gap as regards rodent-borne helminths in some of these countries, such as Bahrain and Oman [26], suggesting that it is essential to conduct more comprehensive studies on rodent-borne diseases, including ectoparasites, in certain countries such as UAE, Jordan, Oman, Iraq, and Bahrain. 

The present review listed a total of 87 species of rodents that occur in the Middle Eastern region. In Iran, 79 species of rodents have been described, of which 15 are considered common, i.e., *Allactaga* sp., *Apodemus witherbyi*, *Dryomys nitedula*, *Gerbillus nanus*, *Jaculus blanfordi*, *Meriones crassus*, *Meriones libycus*, *Meriones persicus*, *Microtus socialis*, *Mus musculus*, *Nesokia indica*, *Rattus norvegicus*, *Rattus rattus*, *Rhombomys opimus*, *Tatera indica* [140,141]. Seventeen species of rodents are reported in Sinai, Egypt: *Acomys cahirinus*, *Acomys russatus*, *Dipodillus dasyurus*, *Eliomys quercinus*, *Gerbillus andersoni*, *Gerbillus gerbillus*, *Gerbillus pyramidium*, *Jaculus jaculus*, *Jaculus orientalis*, *Meriones crassus*, *Meriones sacramenti*, *Meriones tristrami*, *Mus musculus*, *Psammomys obesus*, *Rattus norvegicus*, *Rattus rattus*, *Sekeetamys calurus* [142]. All these common rodents in Iran and Egypt have been reported in this present review.

Some of the rodent ectoparasites addressed in this review have high public and animal health importance. Similar to their impact on humans and other animals, they can also cause certain diseases in the host rodents. Nevertheless, the ectoparasites identified in this review are not always rodent-specific. The host specificity of ectoparasites generally falls within one of three broad categories: (i) ectoparasites specific to rodents, which do not, or only accidentally, infest other mammals (including humans) and birds; (ii) ectoparasites specific to other species that accidentally attack rodents; or (iii) ectoparasites with a broad host range. Rodent fur mites *Radfordia musculi*, *Radfordia musculinus*, *Radfordia affinis,* and *Radfordia ensifera* are mainly found in laboratory rodents [143,144,145]. *Dermanyssus gallinae* and *Ornithonyssus sylviarum* are poultry mites [82,135,146,147,148,149]. They attack humans and other mammals accidentally when exposed to them [150,151]. Some mites were detected on rodents from Egypt, Iran, and Turkey [54,98,121], such as *Macrocheles* spp. *Tryophagus* sp. and *Zygoribatula* sp., which are known as non-parasitic mites [152,153,154]. Reports of these mites parasitizing on rodents may be accidental infestations. On the other hand, some ectoparasites have a broad host range and can infect different birds or mammals, including humans and rodents. An excellent example is the soft tick *Ornithodoros* sp., which can parasitize humans, rodents, livestock, and poultry [155,156]. 

There is considerable public health importance attributed to ectoparasites with a broad host range, mainly if this includes humans, such as *Ctenocephalides canis* and *Ctenocephalides felis*, which can infest dogs, cats, rodents, and humans. These fleas carry multiple zoonotic pathogens, such as *Bartonella*, *Rickettsia felis*, *Dipylidium caninum*, and *Yersinia pestis*, which can be transmitted at the humans–animal interface [157,158,159,160]. The Oriental rat flea *Xenopsylla cheopis* is an essential vector of Bartonellosis, plague, and murine typhus [160,161]. The tropical rat mite *Ornithonyssus bacoti* can transmit numerous pathogens such as *Rickettsia typhi* (murine typhus), *Coxiella burnetti* (Q-fever), and *Trypanosoma cruzi* (Chagas’ disease) [162]. The northern fowl mite *Ornithonyssus sylviarum* can bite humans and cause allergic reactions [163]. *Ornithodoros* sp. has been described to carry Alkhurma hemorrhagic fever virus in Saudi Arabia [164]; *Borrelia* sp. in Egypt [165,166,167], Iran [168,169], Israel [170,171], Jordan [172], Palestine [171] and Turkey [155]; and CCHFV in Iran [156]. *Rhipicephalus* spp. were also found to carry genomes of CCHFV in Iran [156] and Saudi Arabia [173], and *Coxiella*, *Francisella*, *Rickettsia*, *Babesia*, and *Theileria* in Turkey [174]. Moreover, many ectoparasites, such as the house dust mite *Cheyletus* sp., cause allergy in humans [175]. Infestation with *Dermanyssus gallinae* and *Dermanyssus americanus* can cause dermatitis in humans [151,176].

However, meticulously-designed and well-implemented control programs against rodent ectoparasites are of the utmost importance to regional health authorities to control rodent ectoparasite-borne zoonotic diseases effectively. A useful approach would be to limit the spread of rodents themselves. Many of the reviewed articles in this study [30,34,40] stated that rodent abundance is a crucial contributing factor to rodent-borne ectoparasites abundance. The season and location of trapping are other significant determinants of ectoparasites abundance [43,44,47]. More concentration is required to control the three commensal rodents, i.e., *Mus musculus*, *Rattus norvegicus*, and *Rattus rattus*. These rodents have been identified as the most common and extensively-distributed rodent species in the Middle Eastern countries by a previous study [26], and the current study as well. However, rodents are essential components of an ecosystem [140,177], with undeniable benefits for their environment. Therefore, multidisciplinary teams working under the One Health umbrella are necessary to control rodents and rodent-borne ectoparasites with public health importance.

## 5. Conclusions

Rodent ectoparasites, including rodent fleas, lice, mites, and ticks, in Middle Eastern countries, including Cyprus, Egypt, Iran, Israel, Kuwait, Lebanon, Palestine, Qatar, KSA, Turkey, and Yemen, have been reported. In total, 104 flea species, 28 louse species, 134 mite species, and 27 tick species have been detected on 87 rodent species in these countries. Some rodent ectoparasites have substantial public health importance as they are known to carry a broad spectrum of zoonotic pathogens. Besides the One Health approach for rodent control, some other factors such as rodent abundance, season of the year, and trapping location should be considered during the rodent ectoparasite control program. Our systematic review reveals knowledge gaps on rodent ectoparasites in this region, suggesting that it is essential to conduct countrywide in-depth studies on rodent ectoparasites and their public health importance. As the threats of zoonotic diseases increase, including rodent-borne diseases, it is crucial to expand all efforts from all angles to mitigate these threats.

## Supplementary Materials

The following are available online at https://www.mdpi.com/2076-0817/10/2/139/s1, Figure S1: Funnel plots of overall rodent ectoparasite prevalence and subgroup analysis, Table S1: Prisma 2009 checklist, Table S2: Quality assessment of the 113 studied articles, Table S3: Extracted data from the selected 113 studies, Table S4: Rodents, fleas, lice, mites, and ticks on prevailing rodents in the Middle East.
