# Supplementary material for: Rodent Ectoparasites in the Middle East: A Systematic Review and Meta-Analysis
Source: Pathogens. 2021 Jan 31;10(2):139. doi: 10.3390/pathogens10020139 (PMC7911898; doi:10.3390/pathogens10020139)
Supplement: Supplementary file 1 [file pathogens-10-00139-s001.zip › Supplementary documents/Supplementary figure S1.docx]

Rodent Ectoparasites in the Middle East: A Systematic Review and Meta-Analysis

Supplementary figures S4: Funnel plots of overall rodent ectoparasite prevalence and subgroup analysis


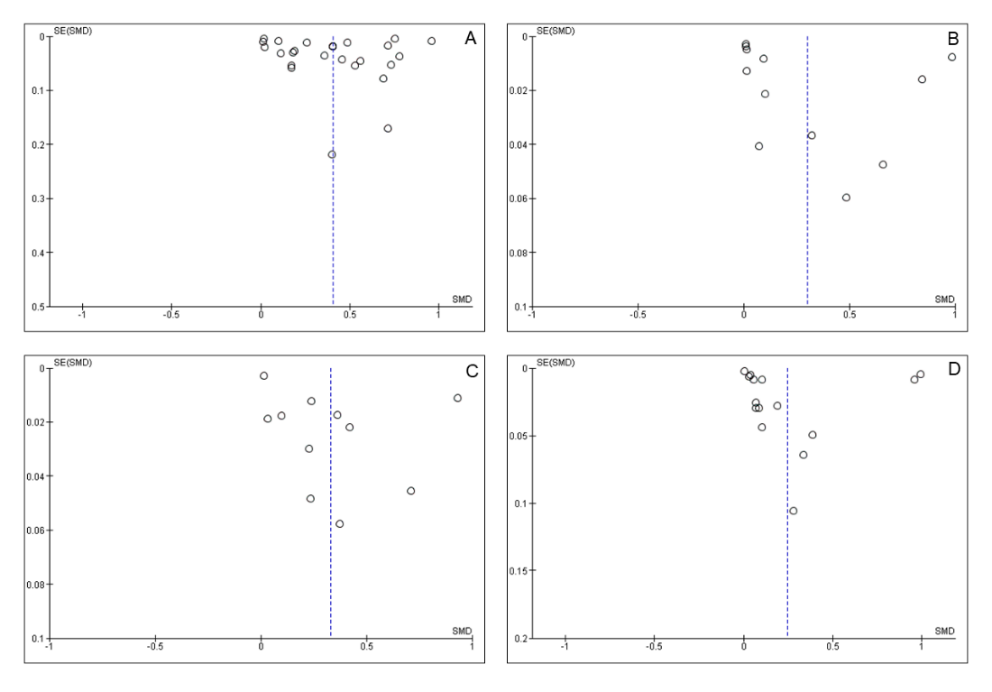


**Supplementary figure S4a:** Funnel plots of overall rodent ectoparasite prevalence; A: Fleas, B: Lice, C: Mites, and D: Ticks.


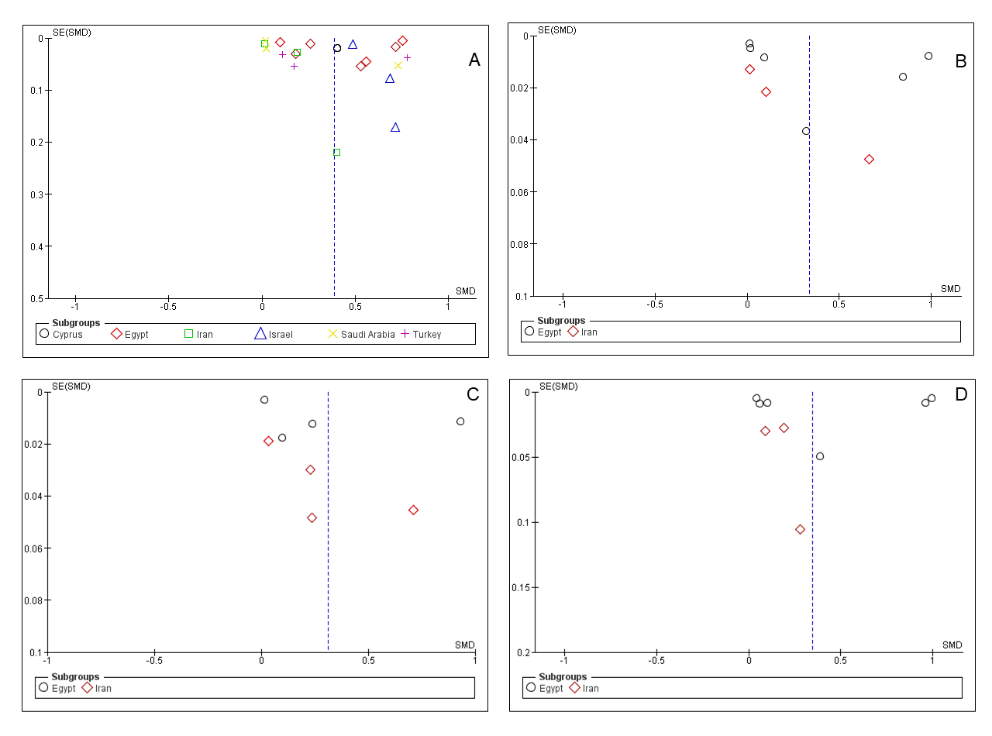


**Supplementary figure S4b:** Funnel plots of country wise overall rodent ectoparasite prevalence; A: Fleas, B: Lice, C: Mites, and D: Ticks


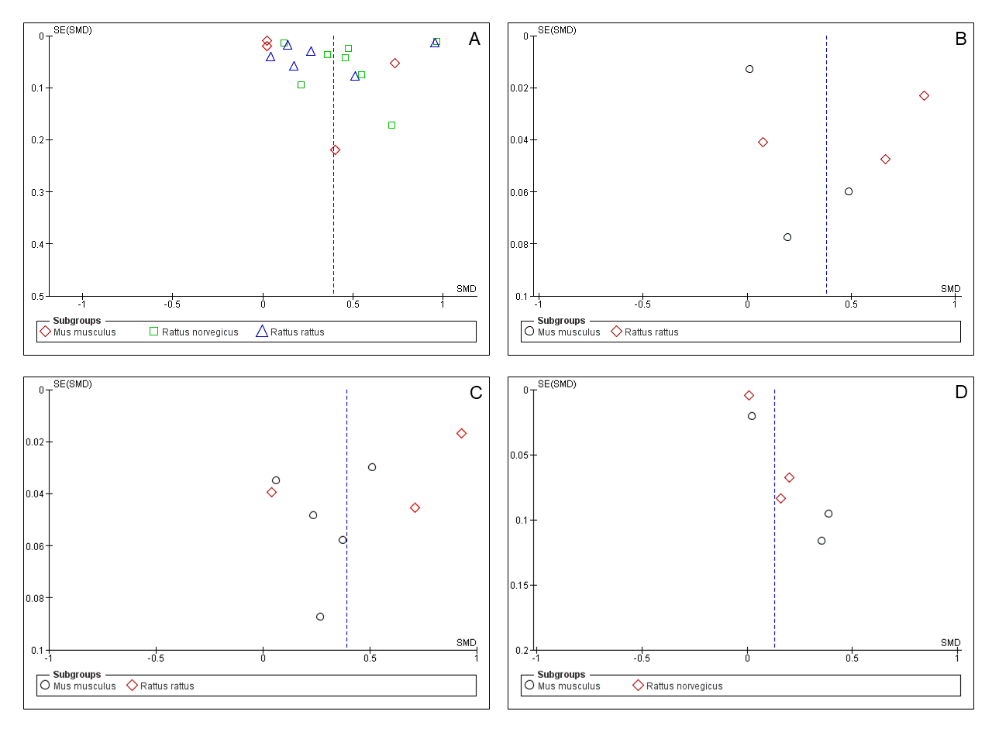


**Supplementary figure S4c:** Funnel plots of rodent host species wise overall ectoparasite prevalence; A: Fleas, B: Lice, C: Mites, and D: Ticks
