# Supplementary material for: Rodent Ectoparasites in the Middle East: A Systematic Review and Meta-Analysis
Source: Pathogens. 2021 Jan 31;10(2):139. doi: 10.3390/pathogens10020139 (PMC7911898; doi:10.3390/pathogens10020139)
Supplement: Supplementary file 1 [file pathogens-10-00139-s001.zip › Supplementary documents/Supplementary table S2.docx]

Rodent Ectoparasites in the Middle East: A Systematic Review and Meta-Analysis

Supplementary Table 2: Quality assessment of the 113 studied articles.

| **Authors** | **Was the sample representative of the target population?** | **Were study participants recruited in an appropriate way?** | **Was the sample size adequate?** | **Were the study subjects and the setting described in detail?** | **Was the data analysis conducted with sufficient coverage of the identified sample?** | **Were objective, standard criteria used for the measurement of the condition?** | **Was the condition measured reliably?** | **Was there appropriate statistical analysis?** | **Are all important confounding factors/subgroups/differences identified and accounted for?** | **Were subpopulations identified using objective criteria?** | **Marks** | **Grades** |
| --- | --- | --- | --- | --- | --- | --- | --- | --- | --- | --- | --- | --- |
|  | 1 | 2 | 3 | 4 | 5 | 6 | 7 | 8 | 9 | 10 |  |  |
| Abd El-Halim et al. [1] | Yes | Yes | Yes | Yes | No | Yes | Unclear | No | Yes | Yes | 7 | High |
| Abdel-Rahman et al. [2] | Yes | Yes | Yes | Yes | Yes | Yes | Unclear | Yes | Yes | Yes | 9 | High |
| Abo-Elmaged and Desoky [3] | Yes | Yes | Unclear | Yes | No | Yes | No | No | No | No | 4 | Low |
| Abu-Madi et al. [4] | Yes | Yes | Yes | Yes | Yes | Yes | Unclear | Yes | Yes | Not applicable | 8 | High |
| Abu-Madi et al. [5] | Yes | Yes | Yes | Yes | Yes | Yes | Unclear | Yes | Yes | Not applicable | 8 | High |
| Acici et al. [6] | Yes | No | No | No | No | Unclear | Unclear | No | No | No | 1 | Low |
| Aktaş [7] | Yes | Yes | Unclear | Yes | Not applicable | Yes | Yes | Not applicable | Not applicable | Not applicable | 5 | Intermediate |
| Al Hindi and Abu-Haddaf [8] | Yes | Yes | No | Yes | No | Yes | Unclear | No | Yes | Yes | 6 | Intermediate |
| Alahmed and Al-Dawood [9] | Yes | Yes | No | Yes | No | Yes | No | No | Unclear | Yes | 5 | Intermediate |
| Al-Awadi et al. [10] | Yes | Unclear | Yes | Yes | No | Yes | Unclear | No | Unclear | Yes | 5 | Intermediate |
| Allam et al. [11] | Yes | Unclear | Unclear | Yes | No | Yes | No | No | No | No | 3 | Low |
| Allymehr et al. [12] | Yes | Yes | Yes | Yes | No | Yes | Yes | No | Yes | Yes | 8 | High |
| Al-Mohammed [13] | Yes | Unclear | No | No | No | Yes | Unclear | No | Unclear | Yes | 3 | Low |
| Alsarraf et al. [14] | Yes | Yes | Unclear | Yes | No | Yes | Yes | No | Yes | Yes | 7 | High |
| Antoniou et al. [15] | Yes | Yes | Yes | Yes | Yes | Yes | Yes | Yes | Yes | Yes | 10 | High |
| Arafa et al. [16] | Yes | Yes | Unclear | Yes | No | Yes | Unclear | No | Yes | Yes | 6 | Intermediate |
| Asiry and Fetoh [17] | Yes | Yes | Yes | Yes | Yes | Yes | Yes | Yes | Yes | Yes | 10 | High |
| Bacot et al. [18] | Yes | Yes | Unclear | Yes | No | Yes | Yes | No | Yes | Yes | 7 | High |
| Bahgat [19] | Yes | Yes | Unclear | Yes | No | Yes | Unclear | No | Yes | Yes | 6 | Intermediate |
| Bajer et al. [20] | Yes | Unclear | Yes | Unclear | Yes | Yes | Yes | Yes | Yes | Yes | 8 | High |
| Bakr et al. [21] | Yes | Yes | No | No | No | Unclear | Unclear | No | Yes | Yes | 4 | Low |
| Bakr et al. [22] | Yes | Unclear | No | No | No | No | Unclear | No | No | No | 1 | Low |
| Bochkov et al. [23] | Yes | Yes | Unclear | No | Not applicable | Yes | Yes | Not applicable | Yes | Not applicable | 5 | Intermediate |
| Bochkov et al. [24] | Yes | Yes | Unclear | Yes | Not applicable | Yes | Yes | Not applicable | Yes | Yes | 7 | High |
| Chegeni et al. [25] | Yes | Yes | No | Yes | Yes | Yes | Yes | Yes | Yes | Yes | 9 | High |
| Christou et al. [26] | Yes | Yes | Yes | Yes | Yes | Yes | Yes | Yes | Yes | Yes | 10 | High |
| Cicek et al. [27] | Yes | Yes | No | Yes | No | Yes | Unclear | No | Yes | Yes | 6 | Intermediate |
| Dahesh and Mikhail [28] | Yes | Yes | No | Yes | Yes | Yes | Yes | No | Yes | Yes | 8 | High |
| Darvishi et al. [29] | Yes | Yes | Not applicable | Yes | Not applicable | Yes | Yes | Not applicable | Yes | Not applicable | 6 | Intermediate |
| el Bahrawy and al Dakhil [30] | Yes | No | No | No | No | Yes | Unclear | No | No | Yes | 3 | Low |
| el Kady et al. [31] | Yes | No | No | Yes | No | Yes | Unclear | No | No | Yes | 4 | Low |
| El Kady et al. [32] | Yes | Unclear | Yes | No | No | Yes | Unclear | No | Yes | Yes | 5 | Intermediate |
| el-Bahrawy and al-Dakhil [33] | Yes | Yes | Unclear | Unclear | No | Yes | Unclear | No | No | Yes | 4 | Low |
| el-Kady et al. [34] | Yes | Yes | No | Yes | No | Yes | Unclear | No | Yes | Yes | 6 | Intermediate |
| el-Kammah et al. [35] | Yes | Unclear | Unclear | No | Not applicable | No | Yes | Not applicable | No | No | 2 | Low |
| Eslami et al. [36] | Yes | Yes | Yes | Yes | Yes | Yes | Unclear | No | Yes | Yes | 8 | High |
| Farhang-Azad and Neronov [37] | Yes | Unclear | Unclear | Unclear | Unclear | Unclear | Unclear | No | Yes | Yes | 3 | Low |
| Gaaboub et al. [38] | Yes | Yes | Yes | Yes | No | Unclear | Unclear | No | Yes | Yes | 6 | Intermediate |
| Gaaboub et al. [39] | Yes | Yes | Yes | No | Yes | Yes | Yes | No | Yes | Yes | 8 | High |
| Garrett and Allred [40] | Yes | Yes | Yes | No | No | Yes | Unclear | No | Unclear | Yes | 5 | Intermediate |
| Gholipoury et al. [41] | Yes | Unclear | No | No | No | No | Yes | No | Yes | Yes | 4 | Low |
| Hamidi and Nassirkhani [42] | Yes | Yes | Yes | Yes | Not applicable | Yes | Unclear | Not applicable | Yes | Yes | 7 | High |
| Hanafi-Bojd et al. [43] | Yes | Unclear | Yes | No | Yes | Yes | Yes | Yes | Yes | Yes | 8 | High |
| Harrison et al. [44] | Yes | Yes | Yes | Yes | Yes | Yes | Yes | Yes | Yes | Yes | 10 | High |
| Hawlena et al. [45] | Yes | Yes | Yes | Yes | Yes | Yes | Yes | Yes | Yes | Yes | 10 | High |
| Hoogstraal and Traub [46] | Yes | Unclear | Unclear | No | No | No | Unclear | No | Yes | Yes | 3 | Low |
| Hoogstraal et al. [47] | Yes | Unclear | Unclear | No | No | No | Yes | No | Yes | Yes | 4 | Low |
| Imam and Salah [48] | Yes | Yes | No | Yes | No | Yes | Unclear | No | No | Yes | 5 | Intermediate |
| Karaer et al. [49] | Yes | Yes | Not applicable | Yes | Not applicable | Yes | Yes | Not applicable | Not applicable | Not applicable | 5 | Intermediate |
| Keskin and Beaucournu [50] | Yes | Yes | Not applicable | Yes | Not applicable | Yes | Yes | Not applicable | Yes | Yes | 7 | High |
| Keskin et al. [51] | Yes | Yes | No | Yes | Not applicable | Yes | Yes | Not applicable | Yes | Yes | 7 | High |
| Keskin et al. [52] | Yes | Yes | No | Yes | No | Yes | Yes | No | No | Yes | 6 | Intermediate |
| Keskin et al. [53] | Yes | No | No | Yes | No | Yes | Yes | No | Yes | Yes | 6 | Intermediate |
| Khajeh et al. [54] | Yes | Yes | Yes | Yes | Yes | Yes | Yes | Yes | Yes | Yes | 10 | High |
| Kia et al. [55] | Yes | Yes | No | Yes | No | Yes | Yes | No | Yes | Yes | 7 | High |
| Kim and Emerson [56] | Yes | Unclear | Unclear | Yes | Unclear | Yes | Unclear | Unclear | Yes | Yes | 5 | Intermediate |
| Krasnov et al. [57] | Yes | Yes | Yes | Yes | Yes | Yes | Yes | Yes | Yes | Yes | 10 | High |
| Krasnov et al. [58] | Yes | Yes | Yes | Yes | Yes | Yes | Yes | Yes | Yes | Yes | 10 | High |
| Krasnov et al. [59] | Yes | Yes | Yes | Yes | Yes | Yes | Yes | Yes | Yes | Yes | 10 | High |
| Krasnov et al. [60] | Yes | Yes | Yes | Yes | No | Yes | Unclear | No | Yes | Yes | 7 | High |
| Krasnov et al. [61] | Yes | Yes | Yes | Yes | Yes | Yes | Yes | Yes | Yes | Yes | 10 | High |
| Krasnov et al. [62] | Yes | Yes | Yes | Yes | Yes | Yes | Yes | Yes | Yes | Yes | 10 | High |
| Krasnov et al. [63] | Yes | Yes | Yes | Yes | Yes | Yes | Yes | Yes | Yes | Yes | 10 | High |
| Krasnov et al. [64] | Yes | Yes | Yes | Yes | Yes | Yes | Yes | Yes | Yes | Yes | 10 | High |
| Lehmann-a [65] | Yes | Yes | Unclear | Yes | Yes | Yes | Yes | Yes | Yes | Yes | 9 | High |
| Lehmann-b [66] | Yes | Yes | Unclear | Yes | Yes | Yes | Yes | Yes | Yes | Yes | 9 | High |
| Lewis [67] | Yes | Unclear | Unclear | Yes | Not applicable | Yes | Unclear | Not applicable | Yes | Yes | 5 | Intermediate |
| Lewis [68] | Yes | Yes | Unclear | Yes | No | Yes | Yes | No | No | Yes | 6 | Intermediate |
| Loftis et al. [69] | Yes | Yes | Yes | Yes | No | Yes | Yes | No | No | Yes | 7 | High |
| Mahdi and Arafa [70] | Yes | Yes | Yes | No | No | Yes | Unclear | No | No | Yes | 5 | Intermediate |
| Mikhail et al. [71] | Yes | Yes | Yes | Yes | No | Yes | No | No | No | Yes | 6 | Intermediate |
| Mohammadi et al. [72] | Yes | Yes | Yes | Yes | Yes | Yes | Yes | Yes | Yes | Yes | 10 | High |
| Moravvej et al. [73] | Yes | Yes | Yes | Yes | No | Yes | Yes | No | Yes | Yes | 8 | High |
| Morick et al. [74] | Yes | Yes | No | Yes | Not applicable | Yes | Yes | Not applicable | Yes | Yes | 7 | High |
| Morick et al. [75] | Yes | Yes | Yes | Yes | Yes | Yes | Yes | Yes | Yes | Yes | 10 | High |
| Morsy et al. [76] | Yes | Yes | Yes | Yes | No | Yes | Unclear | No | Yes | Yes | 7 | High |
| Morsy et al. [77] | Yes | Unclear | Yes | No | No | No | Yes | No | Yes | Yes | 5 | Intermediate |
| Morsy et al. [78] | Yes | Yes | Yes | Yes | No | Yes | Yes | No | Yes | Yes | 8 | High |
| Morsy et al. [79] | Yes | No | No | No | No | Yes | Yes | No | No | Yes | 4 | Low |
| Mostafavi et al. [80] | Yes | Yes | Yes | Yes | Yes | Yes | Yes | No | Yes | Yes | 9 | High |
| Mumcuoglu et al. [81] | Yes | No | No | Yes | No | Yes | Yes | No | Yes | Yes | 6 | Intermediate |
| Mumcuoglu et al. [82] | Yes | No | No | Yes | No | Yes | Yes | No | Yes | Yes | 6 | Intermediate |
| Nasereddin et al. [83] | Yes | Unclear | Unclear | Unclear | Unclear | Yes | Yes | Not applicable | Yes | Yes | 5 | Intermediate |
| Nateghpour et al. [84] | Yes | Yes | No | Yes | No | Yes | No | No | Yes | Yes | 6 | Intermediate |
| Oyoun et al. [85] | Yes | Unclear | Unclear | No | No | Yes | Yes | No | Not applicable | Not applicable | 3 | Low |
| Pourhossein et al. [86] | Yes | Yes | No | Yes | No | Yes | Yes | No | Yes | Yes | 7 | High |
| Psaroulaki et al. [87] | Yes | Yes | No | Yes | No | Yes | Yes | No | Yes | Yes | 7 | High |
| Psaroulaki et al. [88] | Yes | Yes | Yes | Yes | Yes | Yes | Yes | Yes | Yes | Yes | 10 | High |
| Psaroulaki et al. [89] | Yes | Yes | Yes | Yes | No | Yes | Yes | No | Yes | Yes | 8 | High |
| Rahdar et al. [90] | Yes | Yes | No | Yes | No | Yes | Yes | No | Yes | Yes | 7 | High |
| Reeves et al. [91] | Yes | Yes | Unclear | No | No | Yes | Yes | Yes | Yes | Yes | 7 | High |
| Reeves et al. [92] | Yes | Yes | Unclear | No | No | Yes | Yes | Yes | Yes | Yes | 7 | High |
| Rifaat et al. [93] | Yes | Yes | Yes | Yes | No | Yes | Yes | No | Yes | Yes | 8 | High |
| Rzotkiewicz et al. [94] | Yes | Yes | No | No | No | Yes | Yes | No | Yes | Yes | 6 | Intermediate |
| Sanborn and Hoogstraal [95] | Yes | Unclear | Unclear | No | N | Yes | Unclear | No | Yes | Yes | 4 | Low |
| Shamsi et al. [96] | Yes | Unclear | Unclear | Unclear | No | No | Unclear | No | Yes | Yes | 3 | Low |
| Shayan and Rafinejad [97] | Yes | Yes | Yes | Yes | No | Yes | Unclear | No | Yes | Yes | 7 | High |
| Shirazi et al. [98] | Yes | No | Not applicable | Yes | Not applicable | Yes | Yes | Not applicable | No | No | 4 | Low |
| Soliman-a et al. [99] | Yes | Yes | Yes | Yes | Yes | Yes | Yes | Yes | Yes | Yes | 10 | High |
| Soliman-b [100] | Yes | Yes | Yes | Yes | Yes | Yes | Yes | Yes | Yes | Yes | 10 | High |
| Soliman et al. [101] | Yes | Yes | Yes | Unclear | No | Unclear | No | No | Yes | Yes | 5 | Intermediate |
| Stekol'nikov [102] | Yes | Yes | Unclear | No | No | No | Yes | No | No | No | 3 | Low |
| Stekolnikov et al. [103] | Yes | Yes | Unclear | No | No | No | Yes | No | No | No | 3 | Low |
| Tajedin et al. [104] | Yes | Yes | No | Yes | Yes | Yes | Yes | No | Yes | Yes | 8 | High |
| Telmadarraiy et al. [105] | Yes | Yes | No | Yes | Yes | Yes | Yes | Yes | Yes | Yes | 9 | High |
| Uslu et al. [106] | Yes | Yes | Yes | Yes | No | Yes | No | No | Yes | Yes | 7 | High |
| Yeruham et al. [107] | Yes | Yes | Yes | Yes | No | Yes | Yes | No | Yes | Yes | 8 | High |
| Younis et al. [108] | Yes | Yes | Yes | Yes | No | Yes | Yes | No | Yes | Yes | 8 | High |
| Yousefi et al. [109] | Yes | Yes | No | Yes | No | No | Unclear | No | No | Yes | 4 | Low |
| Yousefi et al. [110] | Yes | Unclear | No | No | No | Yes | Yes | No | No | No | 3 | Low |
| Zarei et al. [111] | Yes | Yes | No | Yes | No | Yes | Yes | No | Yes | Yes | 7 | High |
| Zeese et al. [112] | Yes | Yes | Yes | No | No | Yes | Yes | No | Yes | Yes | 7 | High |
| Zendehfili et al. [113] | Yes | Yes | No | Yes | No | Yes | Yes | No | Yes | Yes | 7 | High |

# References

1. Abd El-Halim, A.S.; Allam, K.A.; Metwally, A.M.; El Boraey, A.M. Seasonal variation of infestation rate with lice, tick and mite among rodents in certain Egyptian regions. *Journal of the Egyptian Society of Parasitology* **2009**, *39*, 617-624.

2. Abdel-Rahman, E.H.; Abdelgadir, M.; AlRashidi, M. Ectoparasites burden of House mouse (Mus musculus linnaeus, 1758) from Hai'l of Saudi Arabia. *Saudi Journal of Biological Sciences* **2020**, *27*, 2238-2244.

3. Abo-Elmaged, T.M.; Desoky, A.E.A.S.S. Parasitological survey of rodent in cultivated and reclaimed land at Assiut, Egypt. *Asian Journal of Applied Sciences* **2014**, *7*, 96-101.

4. Abu-Madi, M.A.; Lewis, J.W.; Mikhail, M.; El-Nagger, M.E.; Behnke, J.M. Monospecific helminth and arthropod infections in an urban population of brown rats from Doha, Qatar. *Journal of Helminthology* **2001**, *75*, 313-320.

5. Abu-Madi, M.A.; Behnke, J.M.; Mikhail, M.; Lewis, J.W.; Al-Kaabi, M.L. Parasite populations in the brown rat Rattus norvegicus from Doha, Qatar between years: The effect of host age, sex and density. *Journal of Helminthology* **2005**, *79*, 105-111, doi:10.1079/JOH2005274.

6. Acici, M.; Demirtas, S.; Umur, S.; Gurler, A.T.; Bolukbas, C.S. Infestations of flea species on small, wild mammals in the provinces of Aydin and Manisa in the Aegean Region, Turkey. *Turkish Journal of Veterinary & Animal Sciences* **2017**, *41*, 449-452, doi:10.3906/vet-1610-68.

7. Aktaş, M. Ctenophthalmus harputus, a new Spalax flea from Turkey. *Med Vet Entomol* **1989**, *3*, 23-27, doi:10.1111/j.1365-2915.1989.tb00470.x. RAYYAN-INCLUSION: {"Md Mazharul"=>"Included"}.

8. Al Hindi, A.I.; Abu-Haddaf, E. Gastrointestinal parasites and ectoparasites biodiversity of Rattus rattus trapped from Khan Younis and Jabalia in Gaza strip, Palestine. *Journal of the Egyptian Society of Parasitology* **2013**, *43*, 259-268.

9. Alahmed, A.M.; Al-Dawood, A.S. Rodents and their ectoparasites in Wadi Hanifah, Riyadh City, Saudi Arabia. *Journal of the Egyptian Society of Parasitology* **2001**, *31*, 737-743.

10. Al-Awadi, A.R.; Al-Kazemi, N.; Ezzat, G.; Saah, A.J.; Shepard, C.; Zaghloul, T.; Gherdian, B. Murine typhus in Kuwait in 1978. *Bulletin of the World Health Organization* **1982**, *60*, 283-289.

11. Allam, K.A.; Shalaby, A.A.; Ashour, M.A. Seasonal distribution of fleas infesting rodents in various Egyptian eco-geographical areas and their susceptibility to malathion. *Journal of the Egyptian Society of Parasitology* **2002**, *32*, 405-414.

12. Allymehr, M.; Tavassoli, M.; Manoochehri, M.H.; Ardavan, D. Ectoparasites and gastrointestinal helminths of house mice (mus musculus) from poultry houses in northwest Iran. *Comparative Parasitology* **2012**, *79*, 283-287.

13. Al-Mohammed, H.I. Taxonomical studies of ticks infesting wild rodents from Asir Province in Saudi Arabia. *Journal of the Egyptian Society of Parasitology* **2008**, *38*, 1-8.

14. Alsarraf, M.; Mierzejewska, E.J.; Mohallal, E.M.E.; Behnke, J.M.; Bajer, A. Genetic and phylogenetic analysis of the ticks from the Sinai Massif, Egypt, and their possible role in the transmission of Babesia behnkei. *Exp Appl Acarol* **2017**, *72*, 415-427, doi:10.1007/s10493-017-0164-4. Epub 2017 Aug 28. RAYYAN-INCLUSION: {"Md Mazharul"=>"Included"}.

15. Antoniou, M.; Psaroulaki, A.; Toumazos, P.; Mazeris, A.; Ioannou, I.; Papaprodromou, M.; Georgiou, K.; Hristofi, N.; Patsias, A.; Loucaides, F., et al. Rats as indicators of the presence and dispersal of pathogens in cyprus: Ectoparasites, parasitic helminths, enteric bacteria, and encephalomyocarditis virus. *Vector-Borne and Zoonotic Diseases* **2010**, *10*, 867-873.

16. Arafa, M.S.; Mahdi, A.H.; Khalil, M.S. Seasonal observations on the Cairo spiny mouse, Acomys cahirinus (E. Geoffroy, St. Hilaire, 1803) and its fleas in Egypt. *The Journal of the Egyptian Public Health Association* **1973**, *48*, 60-71.

17. Asiry, K.A.; Fetoh, B.E.A. Occurrence of ectoparasitic arthropods associated with rodents in Hail region northern Saudi Arabia. *Environmental Science and Pollution Research* **2014**, *21*, 10120-10128, doi:10.1007/s11356-014-3016-3.

18. Bacot, A.; Petrie, G.F.; Todd, R.E. The fleas found on rats and other rodents, living in association with man, and trapped in the towns, villages and nile boats of upper Egypt. *Journal of Hygiene* **1914**, *14*, 498-508.

19. Bahgat, I.M. Monthly abundance of rodent and their ectoparasites in newly settled areas, east of lakes, Ismailia Governorate, Egypt. *J Egypt Soc Parasitol* **2013**, *43*, 387-398, doi:10.12816/0006394. RAYYAN-INCLUSION: {"Md Mazharul"=>"Included"}.

20. Bajer, A.; Harris, P.D.; Behnke, J.M.; Bednarska, M.; Barnard, C.J.; Sherif, N.; Clifford, S.; Gilbert, F.S.; Siński, E.; Zalat, S. Local variation of haemoparasites and arthropod vectors, and intestinal protozoans in spiny mice (Acomys dimidiatus) from four montane wadis in the St Katherine Protectorate, Sinai, Egypt. *Journal of Zoology* **2006**, *270*, 9-24.

21. Bakr, M.E.; Morsy, T.A.; Nassef, N.E.; el Meligi, M.A. Mites infesting commensal rodents in Shebin El Kom, Menoufia G., Egypt. *J Egypt Soc Parasitol* **1995**, *25*, 853-859.

22. Bakr, M.E.; Morsy, T.A.; Nassef, N.E.; El Meligi, M.A. Flea ectoparasites of commensal rodents in Shebin El Kom, Menoufia Governorate, Egypt. *Journal of the Egyptian Society of Parasitology* **1996**, *26*, 39-52.

23. Bochkov, A.; Malikov, V.; Arbobi, M. Trichoecius calomysci sp. n. (Acari: Myocoptidae), a new mite species from Iran. *Folia Parasitologica* **1999**, *46*, 316-318.

24. Bochkov, A.; Arbobi, M.; Malikov, V. Notes on mites of the family Myobiidae (Acari: Prostigmata) parasitising rodents (Mammalia: Rodentia) in Iran. *Folia Parasitol (Praha)* **2000**, *47*, 73-77, doi:10.14411/fp.2000.015. RAYYAN-INCLUSION: {"Md Mazharul"=>"Included"}.

25. Chegeni, A.H.; Mostafavi, E.; Mohammadi, A.; Mahmoudi, A.; Kayedi, M.H. The parasitism of Persian jird by immature stages of Hyalomma asiaticum (Acari: Ixodidae) and its identification using molecular approaches in Iran. *Persian Journal of Acarology* **2018**, *7*, 381-392.

26. Christou, C.; Psaroulaki, A.; Antoniou, M.; Toumazos, P.; Ioannou, I.; Mazeris, A.; Chochlakis, D.; Tselentis, Y. Rickettsia typhi and Rickettsia felis in Xenopsylla cheopis and Leptopsylla segnis parasitizing rats in Cyprus. *Am J Trop Med Hyg* **2010**, *83*, 1301-1304, doi:10.4269/ajtmh.2010.10-0118. RAYYAN-INCLUSION: {"Md Mazharul"=>"Included"}.

27. Cicek, H.; Stanyukovich, M.; Yağci, S.; Aktaş, M.; Karaer, Z. Gamasine mite (Parasitiformes: Mesostigmata) infestations of small mammals (Mammalia: Rodentia, Insectivora) in Turkey. *Turkiye Parazitol Derg* **2008**, *32*, 65-70.

28. Dahesh, S.M.; Mikhail, M.W. SURVEILLANCE OF TRYPANOSOMA SPP OF RODENTS AND STUDIES IN THEIR TRANSMISSION PROBABILITY BY FLEAS IN SOME RURAL EGYPTIAN AREAS. *Journal of the Egyptian Society of Parasitology* **2016**, *46*, 157-166.

29. Darvishi, M.M.; Youssefi, M.R.; Changizi, E.; Lima, R.R.; Rahimi, M.T. A new flea from Iran. *Asian Pacific Journal of Tropical Disease* **2014**, *4*, 85-87, doi:<https://doi.org/10.1016/S2222-1808(14)60321-2>.

30. el Bahrawy, A.A.; al Dakhil, M.A. Studies on the ectoparasites (fleas and lice) on rodents in Riyadh and its surroundings, Saudi Arabia. *Journal of the Egyptian Society of Parasitology* **1993**, *23*, 723-735.

31. El Kady, G.A.; Shoukry, A.; Ragheb, D.A.; El Said, A.M.; Habib, K.S.; Morsy, T.A. Mites (acari) infesting commensal rats in Suez Canal zone, Egypt. *J Egypt Soc Parasitol* **1995**, *25*, 417-425.

32. El Kady, G.A.; El Shazly, A.M.; Mikhail, M.W.; Bahgat, I.M. Ectoparasites of commensal rodents in Talkha Center, Dakahlia Governorate, Egypt. *Journal of the Egyptian Society of Parasitology* **2007**, *37*, 825-833.

33. el-Bahrawy, A.A.; al-Dakhil, M.A. Studies on the interrelation between rodents and their ectoparasitic acarines in Riyadh region, Saudi Arabia. *Journal of the Egyptian Society of Parasitology* **1993**, *23*, 675-685.

34. El-Kady, G.A.; Makled, K.M.; Morsy, T.A.; Morsy, Z.S. Rodents, their seasonal activity, ecto- and blood-parasites in Saint Catherine area, South Sinai Governorate, Egypt. *J Egypt Soc Parasitol* **1998**, *28*, 815-826.

35. El-Kammah, K.M.; Oyoun, L.M.; El Kady, G.A. Laelaps sinai sp. nov. (Laelapinae, Laelapidae), a parasite of Gerbillus pyramium in El Arish, North Sinai, Egypt. *J Egypt Soc Parasitol* **1994**, *24*, 167-171.

36. Eslami, A.; Yousefi, A.; Dowling, A.P.G. Prevalence of ectoparasites in black rat (Rattus rattus) from Mangrove forests of Qeshm Island, Iran. *Comparative Clinical Pathology* **2018**, *27*, 1583-1586, doi:10.1007/s00580-018-2777-3.

37. Farhang-Azad, A.; Neronov, V. The flea fauna of the great gerbil (Rhombomys opimus Licht.) in Iran. *Folia Parasitol (Praha)* **1973**, *20*, 343-351.

38. Gaaboub, I.A.; Widaatalla, A.E.E.; Kelada, N.L. Survey of Rats and Mice and Their Ectoparasites in Relation to Cultivated Areas in the Vicinity of Alexandria Governorate, Egypt. *The Journal of Agricultural Science* **1981**, *97*, 551-555.

39. Gaaboub, I.A.; Donia, A.H.; Kelada, N.L.; Abdelkarim, M.E.H. Ectoparasites of some rodents from the edge of the western desert near Alexandria, Egypt. *Insect Science and Its Application* **1982**, *3*, 145-150.

40. Garrett, D.A.; Allred, D.M. Mesostigmatid mites from Turkey, with keys to genera and species. *J Med Entomol* **1971**, *8*, 292-298, doi:10.1093/jmedent/8.3.292. RAYYAN-INCLUSION: {"Md Mazharul"=>"Included"}.

41. Gholipoury, M.; Rezai, H.R.; Namroodi, S.; Arab Khazaeli, F. Zoonotic and non-zoonotic parasites of wild rodents in Turkman Sahra, northeastern Iran. *Iranian Journal of Parasitology* **2016**, *11*, 350-357.

42. Hamidi, K.; Nassirkhani, M. Annotated checklist of fleas (Insecta: Siphonaptera) and lice (Insecta: Anoplura) associated with rodents in Iran, with new reports of fleas and lice. *J Vector Borne Dis* **2019**, *56*, 134-145, doi:10.4103/0972-9062.263715. RAYYAN-INCLUSION: {"Md Mazharul"=>"Included"}.

43. Hanafi-Bojd, A.A.; Shahi, M.; Baghaii, M.; Shayeghi, M.; Razmand, N.; Pakari, A. A study on rodent ectoparasites in Bandar Abbas: The main economic southern seaport of Iran. *Iranian Journal of Environmental Health Science and Engineering* **2007**, *4*, 173-176.

44. Harrison, A.; Robb, G.N.; Alagaili, A.N.; Hastriter, M.W.; Apanaskevich, D.A.; Ueckermann, E.A.; Bennett, N.C. Ectoparasite fauna of rodents collected from two wildlife research centres in Saudi Arabia with discussion on the implications for disease transmission. *Acta Trop* **2015**, *147*, 1-5, doi:10.1016/j.actatropica.2015.03.022. Epub 2015 Mar 27. RAYYAN-INCLUSION: {"Md Mazharul"=>"Included"}.

45. Hawlena, H.; Abramsky, Z.; Krasnov, B.R. Ectoparasites and age-dependent survival in a desert rodent. *Oecologia* **2006**, *148*, 30-39.

46. Hoogstraal, H.; Traub, R. The fleas (Siphonaptera) of Egypt. Host-parasite relationships of rodents of the families Spalacidae, Muridae, Gliridae, Dipodidae, and Hystricidae. *The Journal of the Egyptian Public Health Association* **1965**, *40*, 343-379.

47. Hoogstraal, H.; Kaiser, M.N.; Ormsbee, R.A.; Osborn, D.J.; Hemly, I.; Gaber, S. Hyalomma (Hyalommina) rhipicephaloides Neumann (Ixodoidea: Ixodidae): its identity, hosts, and ecology, and Rickettsia conori, R. prowazeki, and Coxiella burneti infections in rodent hosts in Egypt. *Journal of medical entomology* **1967**, *4*, 391-400.

48. Imam, Z.I.; Salah, A.M. Preliminary notes on typhus amon rodents in U.A.R. *The Journal of the Egyptian Public Health Association* **1966**, *41*, 133-143.

49. Karaer, Z.; Kurtdede, A.; Ural, K.; Sari, B.; Cingi, C.C.; Karakurum, M.C.; Haydardedeoglu, A.E. Demodicosis in a Golden (Syrian) hamster (Mesocricetus auratus). *Ankara Universitesi Veteriner Fakultesi Dergisi* **2009**, *56*, 227-229.

50. Keskin, A.; Beaucournu, J.C. Descriptions of Two New Species and a New Subspecies of the Genus Ctenophthalmus (Insecta: Siphonaptera: Ctenophthalmidae) from Turkey. *J Med Entomol* **2019**, *56*, 1275-1282, doi:10.1093/jme/tjz096. RAYYAN-INCLUSION: {"Md Mazharul"=>"Included"}.

51. Keskin, A.; Selçuk, A.Y.; Kefelioğlu, H. Ticks (Acari: Ixodidae) infesting some small mammals from Northern Turkey with new tick–host associations and locality records. *Experimental and Applied Acarology* **2017**, *73*, 521-526.

52. Keskin, A.; Selçuk, A.; Kefelioğlu, H. Ticks (Acari: Ixodidae) infesting some wild animals and humans in Turkey: notes on a small collection. **2019**, *1*, xx-xx.

53. Keskin, A.; Selçuk, A.Y.; Kefelioğlu, H.; Beaucournu, J.C. Fleas (Insecta: Siphonaptera) collected from some small mammals (Mammalia: Rodentia, Eulipotyphla) in Turkey, with new records and new host associations. *Acta Trop* **2020**, *208*, 105522, doi:10.1016/j.actatropica.2020.105522. RAYYAN-INCLUSION: {"Md Mazharul"=>"Included"}.

54. Khajeh, A.; Razmi, G.; Darvish, J. A study of ectoparasites in wild rodents of the Jaz Murian area in the southeast of Iran. *Asian Pacific Journal of Tropical Disease* **2017**, *7*, 418-421.

55. Kia, E.; Moghddas-Sani, H.; Hassanpoor, H.; Vatandoost, H.; Zahabiun, F.; Akhavan, A.; Hanafi-Bojd, A.; Telmadarraiy, Z. Ectoparasites of rodents captured in bandar abbas, southern iran. *Iran J Arthropod Borne Dis* **2009**, *3*, 44-49.

56. Kim, K.C.; Emerson, K.C. Sucking lice (Anoplura) from Iranian mammals. *Journal of medical entomology* **1971**, *8*, 7-16.

57. Krasnov, B.R.; Shenbrot, G.I.; Khokhlova, I.S.; Degen, A.A.; Rogovin, K.A. On the biology of Sundevall's jird (Meriones crassus Sundevall, 1842) (Rodentia : Gerbillidae) in the Negev Highlands, Israel. In *Mammalia*, 1996; Vol. 60, p 375.

58. Krasnov, B.R.; Shenbrot, G.I.; Medvedev, S.G.; Vatschenok, V.S.; Khokhlova, I.S. Host-habitat relations as an important determinant of spatial distribution of flea assemblages (Siphonaptera) on rodents in the Negev Desert. *Parasitology* **1997**, *114*, 159-173.

59. Krasnov, B.; Shenbrot, G.; Khokhlova, I.; Medvedev, S.; Vatschenok, V. Habitat dependence of a parasite-host relationship: flea (Siphonaptera) assemblages in two gerbil species of the Negev Desert. *J Med Entomol* **1998**, *35*, 303-313, doi:10.1093/jmedent/35.3.303. RAYYAN-INCLUSION: {"Md Mazharul"=>"Included"}.

60. Krasnov, B.R.; Hastriter, M.W.; Medvedev, S.G.; Shenbrot, G.I.; Khokhlova, I.S.; Vatschenok, V.S. Additional records of fleas (siphonaptera) on wild rodents in the southern part of Israel. *Israel Journal of Zoology* **1999**, *45*, 333-340.

61. Krasnov, B.R.; Burdelova, N.V.; Shenbrot, G.I.; Khokhlova, I.S. Annual cycles of four flea species in the central Negev desert. *Med Vet Entomol* **2002**, *16*, 266-276, doi:10.1046/j.1365-2915.2002.00374.x. RAYYAN-INCLUSION: {"Md Mazharul"=>"Included"}.

62. Krasnov, B.R.; Khokhlova, I.S.; Shenbrot, G.I. Density-dependent host selection in ectoparasites: An application of isodar theory to fleas parasitizing rodents. *Oecologia* **2003**, *134*, 365-372.

63. Krasnov, B.R.; Morand, S.; Khokhlova, I.S.; Shenbrot, G.I.; Hawlena, H. Abundance and distribution of fleas on desert rodents: Linking Taylor's power law to ecological specialization and epidemiology. *Parasitology* **2005**, *131*, 825-837.

64. Krasnov, B.R.; Shenbrot, G.I.; Khokhlova, I.S.; Hawlena, H.; Degen, A.A. Sex ratio in flea infrapopulations: number of fleas, host gender and host age do not have an effect. *Parasitology* **2008**, *135*, 1133-1141, doi:10.1017/s0031182008004551.

65. Lehmann, T. Ectoparasite impacts on Gerbillus andersoni allenbyi under natural conditions. *Parasitology* **1992**, *104 ( Pt 3)*, 479-488, doi:10.1017/s0031182000063745.

66. Lehmann, T. Reproductive activity of Synosternus cleopatrae (Siphonaptera: Pulicidae) in relation to host factors. *J Med Entomol* **1992**, *29*, 946-952, doi:10.1093/jmedent/29.6.946. RAYYAN-INCLUSION: {"Md Mazharul"=>"Included"}.

67. Lewis, R.E. A preliminary list of the fleas of Lebanon. *Proceedings of the Royal Entomological Society of London. Series A, General Entomology* **1962**, *37*, 49-60, doi:<https://doi.org/10.1111/j.1365-3032.1962.tb00287.x>.

68. Lewis, R.E. The fleas (Siphonaptera) of Egypt. New records. *The Journal of parasitology* **1966**, *52*, 1167-1171.

69. Loftis, A.D.; Reeves, W.K.; Szumlas, D.E.; Abbassy, M.M.; Helmy, I.M.; Moriarity, J.R.; Dasch, G.A. Surveillance of Egyptian fleas for agents of public health significance: Anaplasma, bartonella, coxiella, ehrlichia, rickettsia, and Yersinia pestis. *American Journal of Tropical Medicine and Hygiene* **2006**, *75*, 41-48.

70. Mahdi, A.H.; Arafa, M.S. Seasonal observations on the house mouse, Mus musculus (Cretzeschmar, 1826), and its fleas in Alexandria, U.A.R. *The Journal of the Egyptian Public Health Association* **1971**, *46*, 106-113.

71. Mikhail, M.W.; Soliman, M.I.; Abd el, H.A. Infestation rate of tick, mite and lice among rodent species in Menoufia governorate, Egypt. *J Egypt Soc Parasitol* **2010**, *40*, 425-438.

72. Mohammadi, A.; Sedaghat, M.M.; Abai, M.R.; Darvish, J.; Mobedi, I.; Mahmoudi, A.; Mostafavi, E. Wild Rodents and Their Ectoparasites in an Enzootic Plague Focus, Western Iran. *Vector Borne Zoonotic Dis* **2020**, *20*, 334-347, doi:10.1089/vbz.2019.2524. Epub 2020 Feb 20. RAYYAN-INCLUSION: {"Md Mazharul"=>"Included"}.

73. Moravvej, G.; Hamidi, K.; Nourani, L.; Bannazade, H. Occurrence of ectoparasitic arthropods (Siphonaptera, Acarina, and Anoplura) on rodents of Khorasan Razavi Province, northeast of Iran. *Asian Pacific Journal of Tropical Disease* **2015**, *5*, 716-720.

74. Morick, D.; Baneth, G.; Avidor, B.; Kosoy, M.Y.; Mumcuoglu, K.Y.; Mintz, D.; Eyal, O.; Goethe, R.; Mietze, A.; Shpigel, N., et al. Detection of Bartonella spp. in wild rodents in Israel using HRM real-time PCR. *Veterinary microbiology* **2009**, *139*, 293-297, doi:10.1016/j.vetmic.2009.06.019.

75. Morick, D.; Krasnov, B.R.; Khokhlova, I.S.; Shenbrot, G.I.; Kosoy, M.Y.; Harrus, S. Bartonella Genotypes in Fleas (Insecta: Siphonaptera) Collected from Rodents in the Negev Desert, Israel. *Applied and Environmental Microbiology* **2010**, *76*, 6864-6869, doi:10.1128/aem.00879-10.

76. Morsy, T.A.; Michael, S.A.; Bassili, W.R.; Saleh, M.S. Studies on rodents and their zoonotic parasites, particularly leishmania, in Ismailiya Governorate, A.R. Egypt. *Journal of the Egyptian Society of Parasitology* **1982**, *12*, 565-585.

77. Morsy, T.A.; Fayad, M.E.; Abou Shady, M.K.; Yousef, N.S. Ectoparasites of rodents in Suez governorate with special reference to fleas. *Journal of the Egyptian Society of Parasitology* **1986**, *16*, 457-468.

78. Morsy, T.A.; el-Ela, R.G.; el Gozamy, B.M. The commensal rodents and their flea fauna in Alexandria City, Egypt. *Journal of the Egyptian Society of Parasitology* **1988**, *18*, 11-28.

79. Morsy, T.A.; El Bahrawy, A.F.; El Dakhil, M.A. Ecto- and blood parasites affecting Meriones rex trapped in Najran, Saudi Arabia. *J Egypt Soc Parasitol* **2001**, *31*, 399-405.

80. Mostafavi, E.; Shahraki, A.H.; Japoni-Nejad, A.; Esmaeili, S.; Darvish, J.; Sedaghat, M.M.; Mohammadi, A.; Mohammadi, Z.; Mahmoudi, A.; Pourhossein, B., et al. A Field Study of Plague and Tularemia in Rodents, Western Iran. *Vector-Borne and Zoonotic Diseases* **2017**, *17*, 247-253, doi:10.1089/vbz.2016.2053.

81. Mumcuoglu, K.Y.; Frish, K.; Sarov, B.; Manor, E.; Gross, E.; Gat, Z.; Galun, R. Ecological studies on the brown dog tick Rhipicephalus sanguineus (Acari: Ixodidae) in southern Israel and its relationship to spotted fever group rickettsiae. *Journal of medical entomology* **1993**, *30*, 114-121.

82. Mumcuoglu, K.Y.; Ioffe-Uspensky, I.; Alkrinawi, S.; Sarov, B.; Manor, E.; Galun, R. Prevalence of vectors of the spotted fever group Rickettsiae and murine typhus in a Bedouin town in Israel. *Journal of Medical Entomology* **2001**, *38*, 458-461, doi:10.1603/0022-2585-38.3.458.

83. Nasereddin, A.; Risheq, A.; Harrus, S.; Azmi, K.; Ereqat, S.; Baneth, G.; Salant, H.; Mumcuoglu, K.Y.; Abdeen, Z. Bartonella species in fleas from Palestinian territories: Prevalence and genetic diversity. *Journal of Vector Ecology* **2014**, *39*, 261-270.

84. Nateghpour, M.; Akhavan, A.A.; Hanafi-Bojd, A.A.; Telmadarraiy, Z.; Ayazian Mavi, S.; Hosseini-Vasoukolaei, N.; Motevalli-Haghi, A.; Akbarzadeh, K. Wild rodents and their ectoparasites in Baluchistan area, southeast of Iran. *Tropical Biomedicine* **2013**, *30*, 72-77.

85. Oyoun, L.M.; el Kammah, K.M.; el Kady, G.A. The fur mite Listrophorus arishi: sp. nov. (Listrophorinae, Listrophoridae) of jerboes in North Sinai, Egypt. *J Egypt Soc Parasitol* **1994**, *24*, 173-176.

86. Pourhossein, B.; Esmaeili, S.; Gyuranecz, M.; Mostafavi, E. Tularemia and plague survey in rodents in an earthquake zone in southeastern Iran. *Epidemiology and Health* **2015**, *37*.

87. Psaroulaki, A.; Antoniou, M.; Papaeustathiou, A.; Toumazos, P.; Loukaides, F.; Tselentis, Y. First detection of Rickettsia felis in Ctenocephalides felis fleas parasitizing rats in Cyprus. *Am J Trop Med Hyg* **2006**, *74*, 120-122.

88. Psaroulaki, A.; Antoniou, M.; Toumazos, P.; Mazeris, A.; Ioannou, I.; Chochlakis, D.; Christophi, N.; Loukaides, P.; Patsias, A.; Moschandrea, I., et al. Rats as indicators of the presence and dispersal of six zoonotic microbial agents in Cyprus, an island ecosystem: a seroepidemiological study. *Transactions of the Royal Society of Tropical Medicine and Hygiene* **2010**, *104*, 733-739, doi:10.1016/j.trstmh.2010.08.005.

89. Psaroulaki, A.; Chochlakis, D.; Ioannou, I.; Angelakis, E.; Tselentis, Y. Presence of Coxiella burnetii in Fleas in Cyprus. *Vector-Borne and Zoonotic Diseases* **2014**, *14*, 685-687, doi:10.1089/vbz.2013.1399.

90. Rahdar, M.; Vazirianzadeh, B.; Rointan, E.S.; Amraei, K. Identification of collected ectoparasites of rodents in the west of Khuzestan Province (Ahvaz and Hovizeh), southwest of Iran. *Asian Pacific Journal of Tropical Disease* **2015**, *5*, 627-631.

91. Reeves, W.K.; Szumlas, D.E.; Moriarity, J.R.; Loftis, A.D.; Abbassy, M.M.; Helmy, I.M.; Dasch, G.A. Louse-borne bacterial pathogens in lice (Phthiraptera) of rodents and cattle from Egypt. *Journal of Parasitology* **2006**, *92*, 312-318, doi:10.1645/0022-3395(2006)92[312:BR]2.0.CO;2.

92. Reeves, W.K.; Loftis, A.D.; Szumlas, D.E.; Abbassy, M.M.; Helmy, I.M.; Hanafi, H.A.; Dasch, G.A. Rickettsial pathogens in the tropical rat mite Ornithonyssus bacoti (Acari: Macronyssidae) from Egyptian rats (Rattus spp.). *Experimental and Applied Acarology* **2007**, *41*, 101-107, doi:10.1007/s10493-006-9040-3.

93. Rifaat, M.A.; Morsy, T.A.; Abdel Mawla, M.M. Seasonal activity of Rattus norvegicus and flea index in Port Said Governorate, Egypt. *J Egypt Soc Parasitol* **1981**, *11*, 525-532.

94. Rzotkiewicz, S.; Gutiérrez, R.; Krasnov, B.R.; Morick, D.; Khokhlova, I.S.; Nachum-Biala, Y.; Baneth, G.; Harrus, S. Novel evidence suggests that a 'Rickettsia felis-like' organism is an endosymbiont of the desert flea, Xenopsylla ramesis. *Mol Ecol* **2015**, *24*, 1364-1373, doi:10.1111/mec.13106. Epub 2015 Mar 6. RAYYAN-INCLUSION: {"Md Mazharul"=>"Included"}.

95. Sanborn, C.C.; Hoogstraal, H. *Some mammals of Yemen and their ectoparasites*; Chicago Natural History Museum: Chicago, 1953.

96. Shamsi, M.; Stekolnikov, A.A.; Saboori, A.; Hakimitabar, M.; Golpayegani, A.Z. Contributions to the fauna of chigger mites (Acariformes: Trombiculidae) of Iran. *Zootaxa* **2020**, *4834*, 301-355.

97. Shayan, A.; Rafinejad, J. Arthropod parasites of rodents in Khorram Abbad district, Lorestan Provincen of Iran. *Iranian Journal of Public Health* **2006**, *35*, 70-76.

98. Shirazi, S.; Bahadori, F.; Mostafaei, T.S.; Ronaghi, H. First report of Polyplax sp. in a Persian squirrel (Scuirus anomalus) in Tabriz, Northwest of Iran. *Türkiye parazitolojii dergisi / Türkiye Parazitoloji Derneǧi = Acta parasitologica Turcica / Turkish Society for Parasitology* **2013**, *37*, 299-301, doi:10.5152/tpd.2013.3085.

99. Soliman, S.; Main, A.J.; Marzouk, A.S.; Montasser, A.A. Seasonal studies on commensal rats and their ectoparasites in a rural area of Egypt: The relationship of ectoparasites to the species, locality, and relative abundance of the host. *Journal of Parasitology* **2001**, *87*, 545-553.

100. Soliman, S.; Marzouk, A.S.; Main, A.J.; Montasser, A.A. Effect of sex, size, and age of commensal rat hosts on the infestation parameters of their ectoparasites in a rural area of Egypt. *J Parasitol* **2001**, *87*, 1308-1316, doi:10.1645/0022-3395(2001)087[1308:EOSSAA]2.0.CO;2. RAYYAN-INCLUSION: {"Md Mazharul"=>"Included"}.

101. Soliman, M.I.; Abd El-Halim, A.S.; Mikhail, M.W. Rodent borne diseases and their fleas in Menoufia Governorate, Egypt. *Journal of the Egyptian Society of Parasitology* **2010**, *40*, 107-117.

102. Stekol'nikov, A.A. A new subgenus and species of the chigger mite genus Neotrombicula (Acari: Trombiculidae). *Acarologia* **1999**, *40*, 407-412.

103. Stekolnikov, A.A.; Al-Ghamdi, S.Q.; Alagaili, A.N.; Makepeace, B.L. First data on chigger mites (Acariformes: Trombiculidae) of Saudi Arabia, with a description of four new species. *Systematic and Applied Acarology* **2019**, *24*, 1937-1963.

104. Tajedin, L.; Rassi, Y.; Oshaghi, M.; Telmadarraiy, Z.; Akhavan, A.; Abai, M.; Arandian, M. Study on Ectoparasites of Rhombomys opimus, the Main Reservoir of Zoonotic Cutaneous Leishmaniasis in Endemic Foci in Iran. *Iran J Arthropod Borne Dis* **2009**, *3*, 41-45.

105. Telmadarraiy, Z.; Vatandoost, H.; Mohammadi, S.; Akhavan, A.A.; Abai, M.R.; Rafinejad, J.; Kia, E.B.; Naini, F.F.; Jedari, M.; Aboulhasani, M. Determination of Rodent Ectoparasite Fauna in Sarpole-Zahab District, Kermanshah Province, Iran, 2004-2005. *Iranian Journal of Arthropod-Borne Disease* **2007**, *1*, 5.

106. Uslu, U.; Dik, B.; Gökçen, A. Ectoparasites of the ground squirrel (Citellus citellus (L.)) in Turkey. *Turkiye Parazitol Derg* **2008**, *32*, 142-145.

107. Yeruham, I.; Hadani, A.; Galker, F.; Rosen, S. The occurrence of Ixodes-Eldaricus (Dzhaparidze, 1950) (Acarina, Ixodidae) in Israel. *Acarologia* **1995**, *36*, 191-193.

108. Younis, T.A.; Fayad, M.E.; el Hariry, M.A.; Morsy, T.A. Interaction between acari ectoparasites and rodents in Suez Governorate, Egypt. *Journal of the Egyptian Society of Parasitology* **1995**, *25*, 377-394.

109. Yousefi, A.; Nosrati, M.R.C.; Karimi, A.; Naisi, S. Leptopsylla taschenbergi taschenbergi (Siphonaptera: Leptopsyllidae), new flea from Iran. *Asian Pacific Journal of Tropical Disease* **2015**, *5*, 606-607.

110. Yousefi, A.; Rahbari, S.; Eslami, A. Ectoparasites associated with small mammals (orders Insectivora, Eulipotyphla, and Rodentia) in Razan plain, western region of Iran. *Comparative Clinical Pathology* **2018**, *27*, 667-671.

111. Zarei, Z.; Mohebali, M.; Heidari, Z.; Kia, E.B.; Azarm, A.; Bakhshi, H.; Davoodi, J.; Hassanpour, H.; Roohnavaz, M.; Khodabakhsh, M., et al. Wild Rodent Ectoparasites Collected from Northwestern Iran. *Journal of Arthropod-Borne Diseases* **2017**, *11*, 36-41.

112. Zeese, W.; Khalaf, S.A.; Abou el-Ela, R.G.; Morsy, T.A. Rodents and their ectoparasites in Sharkia Governorate, Egypt. *Journal of the Egyptian Society of Parasitology* **1990**, *20*, 827-835.

113. Zendehfili, H.; Zahirnia, A.H.; Maghsood, A.H.; Khanjani, M.; Fallah, M. Ectoparasites of rodents captured in Hamedan, Western Iran. *Journal of Arthropod-Borne Diseases* **2015**, *9*, 267-273.
