# Supplementary material for: Rodent Ectoparasites in the Middle East: A Systematic Review and Meta-Analysis
Source: Pathogens. 2021 Jan 31;10(2):139. doi: 10.3390/pathogens10020139 (PMC7911898; doi:10.3390/pathogens10020139)
Supplement: Supplementary file 1 [file pathogens-10-00139-s001.zip › Supplementary documents/Supplementary table S3.docx]

Rodent Ectoparasites in the Middle East: A Systematic Review and Meta-Analysis

# Supplementary table S3: Extracted data from the selected studies

| **Authors** | **Countries and Years of study** | **Rodents detail** | **Ectoparasites detail** | **Reported determinants of ectoparasite abundance** | **Remarks** |
| --- | --- | --- | --- | --- | --- |
| Abd El-Halim et al. [1] | Egypt | *Acomys cahirinus* (N=40)  *Mus musculus* (N=207)  *Rattus norvegicus* (N=464)  *Rattus rattus alexandrinus* (N=285)  *Rattus rattus frugivorous* (N=184)  R=1180, Rm=282, Rt=121, Rl=110 | **Lice** (L=110, LiR=9.32%, LI=0.09)  *Polyplax spinulosa*  **Mites** (M=496, MiR=23.9%, MI=0.42)  *Dermanyssus gallinae*  *Echinolaelaps echidninus*  *Haemolaelaps glasgowi*  *Ornithonyssus bacoti*  **Ticks** (T=143, TiR=10.25%, TI=0.12)  *Hyalomma dromedarii*  *Rhipicephalus sanguineus* | Ectoparasite abundance can vary with geographical area and rodent species | Spelling is corrected from *spinulose* to *spinulosa*, and from *dromedarrii* to *dromedarii.* The correct name of *Echinolaelaps echinolelaps* is *Echinolaelaps echidninus* |
| Abdel-Rahman et al. [2] | Saudi Arabia; 2012-13 | *Mus musculus* (f=29, m=41)  R=70, Rf=51, Rl=34, Rm=26 | **Fleas** (F=339, FiR=72.86%, FI=4.84)  *Xenopsylla cheopis* (N=339)  **Lice** (L=76, LiR=48.57%, LI=1.09)  *Polyplax serrata* (N=37)  *Polyplax spinulosa* (N=39)  **Mites** (M=99, MiR=37.14%, MI=1.41)  *Echinolaelaps echidninus* (N=99) | Rodent sex is has no effect on ectoparasite infestation rate. However, rodent trapping location has, as it is higher in wild habitat than residential areas. | *Laelaps echidninus* is a synonyme of *Echinolaelaps echidninus* |
| Abo-Elmaged and Desoky [3] | Egypt; 2012 | *Arvicanthis niloticus*  *Rattus rattus alexandrinus*  *Rattus rattus frugivorous* | **Fleas**  *Leptopsylla segnis*  *Xenopsylla cheopis*  **Lice**  *Polyplax spinulosa*  **Mites**  *Cheyletus zaheri*  *Hypoaspis koseii*  **Ticks**  *Amblyomma* sp. | There is no specific relationship between the rodent species and ectoparasites species. The ectoparasite population is positively correlated with rodent populations and different land types. | Spelling is corrected from *spinulosia* to *spinulosa* |
| Abu-Madi et al. [4] | Qatar; 1998-99 | *Rattus norvegicus* (f*=*84, m=52)  R=136, Rf=62 | **Fleas** (FiR=45.59%)  *Xenopsylla astia* | The abundance of rodents was higher in summer. The abundance of fleas is higher among juveniles than among adults in summer. In winter, age and sex of rodents did not affect flea abundance. |  |
| Abu-Madi et al. [5] | Qatar; 2002-03 | *Rattus norvegicus* (f=96, m=83)  R=179, Rf=64 | **Fleas** (FiR=35.75%)  *Xenopsylla astia* | The abundance of the fleas differed between years with an abundance of rodents, higher on juvenile rodents compared with adults, and in females compared to male rodents. |  |
| Acici et al. [6] | Turkey; 2015-16 | *Apodemus flavicollis* (N=5)  *Apodemus witherbyi* (N=1)  *Microtus levis* (N=2)  *Mus macedonicus* (N=39)  R=47, Rf=7 | **Fleas** (FiR=14.89%)  *Nosopsyllus fasciatus*  *Stenoponia tripectinata* | There may be some association between the rodent species and ectoparasites species. Fleas were detected only on *Mus macedonicus* and *Microtus levis*. |  |
| Aktaş [7] | Turkey | *Spalax leucodon* | **Fleas**  *Ctenophthalmus harputus* |  |  |
| Al Hindi and Abu-Haddaf [8] | Palestine; 2008-09 | *Rattus rattus* (f=15, m=26)  R=41, Rf=7, Rl=3 | **Fleas** (FiR=17.07%)  *Xenopsylla cheopis*  **Lice** (LiR=7.32%)  *Polyplax spinulosa* | A considerable amount of garbage in residential areas can increase the rodent population, and with it, the ecotprasites abundance. |  |
| Alahmed and Al-Dawood [9] | KSA; 2000-01 | *Acomys dimidiatus* (N=13)  *Meriones libycus* (N=9)  *Mus musculus* (N=2)  *Rattus rattus alexandrinus* (N=4)  *Rattus rattus frugivorous* (N=6)  *Rattus rattus rattus* (N=14)  R=48 (f=20, m=28, Rf=1, Rt=4 | **Fleas** (F=3, FiR=2.08%, FI=0.06%)  *Xenopsylla* sp*.*  **Ticks** (T=12, TiR=10.42%, TI=0.25)  *Rhipicephalus turanicus* | Rodent infestation as well as its ectoparasite infestation may decrease during summer due to severe weather conditions in the deserts. |  |
| Al-Awadi et al. [10] | Kuwait; 1978 | *Rattus norvegicus* | **Fleas**  *Xenopsylla astia*  **Lice**  *Polyplax cannomydis*  *Polyplax spinulosa*  **Mites**  *Laelaps nuttalli*  *Ornithonyssus bacoti* | Rodents most commonly infest lower socioeconomic grade houses. Rodent and its ectoparasite infestion is seasonally influenced; it begins to increase during April, reaches a peak in May, and decreases again by September. | Spelling is corrected from *Ornythonyssus* to *Ornithonyssus* and *nutalli* to *nuttalli* and *cenomudis* to *cannomydis* |
| Allam et al. [11] | Egypt | *Acomys cahirinus*  *Arvicanthis niloticus*  *Mus musculus*  *Rattus norvegicus*  *Rattus rattus alexandrinus*  *Rattus rattus frugivorous* | **Fleas**  *Ctenocephalides canis*  *Leptopsylla segnis*  *Xenopsylla cheopis* | Rodent flea infestation can vary according to rodent host species, rodent trapping location, and season of the year. |  |
| Allymehr et al. [12] | Iran; 2010 | *Mus musculus* (f=52, m=25)  R=77, Rm=18, Rl=1 | **Lice** (L=1, LiR=1.3%, LI=0.01)  *Polyplax serrata*  **Mites** (M=27, MiR=23.38%, MI=0.4)  *Dermanyssus gallinae* (N=24)  *Myocoptes musculinus* (N=1)  *Ornithonyssus bacoti* (N=2v | Presence of rodents in the poultry farms may directly contribute to ectoparasite abundance among the poultry population. |  |
| Al-Mohammed [13] | KSA; 2006 | *Acomys dimidiatus* (N=20)  *Gerbillus cheesmani* (N=1)  *Meriones rex* (N=19)  R=40 | **Ticks** (T=106, TI=2.65)  *Rhipicephalus sanguineus*  *Rhipicephalus turanicus* |  |  |
| Alsarraf et al. [14] | Egypt; 2012 | *Acomys dimidiatus*  *Acomys russatus*  *Dipodillus dasyurus*  *Sekeetamys calurus* | **Ticks**  *Hyalomma dromedarii*  *Hyalomma* sp.  *Rhipicephalus* sp. |  |  |
| Antoniou et al. [15] | Cyprus; 2000-03 | *Mus musculus* (N=3)  *Rattus norvegicus* (N=402)  *Rattus rattus frugivorous* (N=220)  R=625, Rf=252, Rt=3 | **Fleas** (F=1035, FiR=40.32%, FI=1.66)  *Ctenocephalides canis* (N=5)  *Ctenocephalides felis* (N=250)  *Leptopsylla segnis* (N=45)  *Nosopsyllus fasciatus* (N=7)  *Xenopsylla cheopis* (N=728)  **Ticks** (T=3, TiR=0.48%, TI=0.01)  *Non identified ticks* | Rodent abundance differs with the season of the year and the trapping site. It is higher in the summer. *Xenopsylla cheopis* was the most abundant flea (70%) , followed by *Ctenocephalides felis* (24%. Ectoparasite infestation also varies according to the rodent species; *Rattus norvegicus* was the most infested with ectoparasites. |  |
| Arafa et al. [16] | Egypt | *Acomys cahirinus* | **Fleas**  *Leptopsylla segnis*  *Parapulex chephrenis*  *Xenopsylla cheopis* | Rodent and ectoparasite abundance can be influenced by rodent trapping location, season of the year, and rodent gender. |  |
| Asiry and Fetoh [17] | KSA; 2012-13 | *Acomys dimidiatus* (N=22)  *Rattus rattus alexandrinus* (N=45)  *Rattus rattus frugivorous* (N=55)  *Rattus rattus rattus* (N=628)  (f=315, m=435)  R=750, Rf=13, Rm=272, Rt=23, Rl=8 | **Fleas** (F=40, FiR=1.73%, FI=0.05)  *Xenopsylla cheopis*  *Xenopsylla conformis mycerini*  **Lice** (L=152, LiR=1.07%, LI=0.20)  *Polyplax serrata*  **Mites** (M=299, MiR=36.27%, MI=0.40)  *Echinolaelaps echidninus*  *Laelaps nuttalli*  **Ticks** (T=796, TiR=3.07%, TI=1.06)  *Rhipicephalus sanguineus*  *Rhipicephalus turanicus* | The abundance of rodents is associated with trapping location, but it is not true for ectoparasites abundance. | Spelling is corrected from *nuttali* to *nuttalli; Laelaps echidninus* is a synonyme of *Echinolaelaps echidninus* |
| Bacot et al. [18] | Egypt; 1912-13 | *Acomys cahirinus*  *Arvicanthis niloticus*  *Rattus norvegicus*  *Rattus rattus* | **Fleas**  *Ctenocephalides felis*  *Echidnophaga gallinacea*  *Leptopsylla segnis*  *Pulex irritans*  *Xenopsylla cheopis*  *Xenopsylla cleopatrae* | *Xenopsylla cheopis* was the most commonly identified species of fleas (90%). | *Rattus rattus is a* synonym of *Mus rattus, Rattus norvegicus is a synonym of Mus norvegicus*, and *Leptopsylla segnis* is a synonym of *Leptopsylla musculi.* Spelling is corrected from *Ctenocephalus* to *Ctenocephalides* |
| Bahgat [19] | Egypt; 2009 | *Mus musculus* (N=115)  *Rattus norvegicus* (N=12)  *Rattus rattus alexandrinus* (N=40)  *Rattus rattus frugivarous* (N=54)  R=221 | **Flae** (F=874, FI=3.94)  *Leptopsylla segnis* (N=14)  *Nosopsyllus sinaiensis* (N=7)  *Pulex irritans* (N=44)  *Stenoponia tripectinata* (N=11)  *Xenopsylla cheopis* (N=742)  *Xenopsylla ramesis* (N=56)  **Lice** (L=250, LI=1.13)  *Polyplax spinulosa* (N=250)  **Mites** (M=549, MI=2.48)  *Dermanyssus gallinae* **(**N=70)  *Eulaelaps stabularis* (N=22)  *Haemogamasus pontiger* (N=14)  *Hirstionyssus isabellinus* (N=18)  *Laelaps nuttalli* (N=241)  *Ornithonyssus bacoti* (N=184)  **Ticks** (T=74, TI=0.33)  *Hyalomma* sp. (N=47)  *Rhipicephalus* sp. (N=27) | Rodent abundance is seasonally-influenced; highest in the summer and lowest in winter. Ectoparasite abundance is influenced by rodent species (highest in *Rattus norvegicus* and lowest in *Mus musculus)* and trapping location (higher in outdoor than indoor). | *Leptopsylla segnis* is the scientific alternative name of *Ctenopsyllus segnis.*  The spelling *Nosopsylla* is corrected to  *Nosopsyllus* |
| Bajer et al. [20] | Egypt | *Acomys dimidiatus*  R=162, Rf=29, Rl=52 | **Fleas** (FiR=17.9%)  *Parapulex chephrenis*  *Xenopsylla dipodill*  **Mites**  *Dermanyssus sanguineus*  *Haemolaelaps* sp*.*  **Lice** (LiR=32.1%)  *Polyplax brachyrrhyncha*  *Polyplax oxyrrhyncha* | Ectoparasite abundance is affected by rodent trapping location. *Parapulex chephrenis* fleas and *Polyplax brachyrrhynchai* lice are the most abundant ectoparasites. The lice infestation rate can differ according to the gender and age of rodents. | *Dermanyssus sanguineus,* synonymous with *Liponyssoides sanguineus,* was formerly known as *Allodermanyssus* *sanguineus.* The spelling *chephrensis* is corrected to *chephrenis* and *brachyrrhycha* to *brachyrrhyncha* |
| Bakr et al. [21] | Egypt | *Mus musculus*  *Rattus norvegicus*  *Rattus rattus alexandrinus* | **Mites**  Dermanyssus gallinae  *Dermanyssus sanguineus*  Laelaps nuttalli  Ornithonyssus bacoti | Mite infestation is affected by rodent species and gender. Higher infestation was recorded in *Rattus rattus* and in female rodents. | *Dermanyssus sanguineus,* synonymous with *Liponyssoides sanguineus* is the modern synonym of *Allodermanyssus sanguineus* |
| Bakr et al. [22] | Egypt | *Mus musculus* (N=29)  *Rattus norvegicus* (N=37)  *Rattus rattus* (N=54)  R=120 (f=67, m=53, Rf=67 | **Fleas** (F=71; FiR=55.83%, FI=0.59)  *Ctenocephalides felis* (N=4)  *Leptopsylla segnis* (N=8)  *Pulex irritans* (N=9)  *Xenopsylla cheopis* (N=41)  *Xenopsylla ramesis* (N=9) | The abundance of captured rodents and fleas was the highest in spring, gradually decreases in summer and autumn. In winter, no fleas were detected on rodents. |  |
| Bochkov et al. [23] | Iran | *Calomyscus* sp. | **Mites**  *Trichoecius calomysci* |  |  |
| Bochkov et al. [24] | Iran; 1997-98 | *Acomys cahirinus*  *Dryomys nitedula*  *Gerbillus cheesmani*  *Meriones libycus*  *Mus musculus*  R=79 | **Mites**  *Myobia murismusculi*  *Radfordia acomys*  *Radfordia affinis*  *Radfordia dyromys*  *Radfordia merioni*  *Radfordia persica* |  |  |
| Chegeni et al. [25] | Iran; 2016-18 | *Meriones persicus*  R=23 | **Ticks** (T=60, TI=2.61)  *Hyalomma asiaticum*  *Hyalomma* Koch |  |  |
| Christou et al. [26] | Cyprus; 2000-03 | *Rattus norvegicus* (N=402)  *Rattus rattus frugivorous* (N=220)  R=622 | **Fleas** (F=1035, FI=1.66)  *Ctenocephalides canis* (N=5)  *Ctenocephalides felis* (N=250)  *Leptopsylla segnis* (N=45)  *Nosopsyllus fasciatus* (N=7)  *Xenopsylla cheopis* (N=728) | Fleas distribution differ with geographical difference. |  |
| Cicek et al. [27] | Turkey; 1996-97 | *Apodemus sylvaticus (*N=2)  *Cricetulus migratorius* (N=34)  *Meriones persicus* (N=1)  *Meriones tristrami* (N=4)  *Mesocricetus auratus* (N=4)  *Mesocricetus brandti* (N=2)  *Microtus arvalis* (N=7)  *Microtus guentheri* (N=2)  *Mus musculus (*N=56)  *Spermophilus citellus* (N=9)  R=121, Rm=94 | **Mites** (M=119, MiR=77.69%, MI=0.98)  *Eulaelaps stabularis* (N=7)  *Haemogamasus horridus* (N=1)  *Haemogamasus nidiformis* (N=6)  *Haemogamasus zachvatkini* (N=1)  *Haemolaelaps androgynus* (N=1)  *Haemolaelap glasgowi* (N=2)  *Haemolaelaps* (N=4)  *Hirstionyssus eversmani* (N=1)  *Hirstionyssus isabellinus* (N=8)  *Hirstionyssus* sp. (N=3)  *Laelaps algericus* (N=2)  *Laelaps hilaris* (N=2)  *Laelaps jettmari* (N=72)  *Laelaps kochi* (N=1)  *Macrocheles muscaedomestica* (N=3)  *Ornithonyssus bacoti* (N=5) |  | *Citellus citellus* is changed to homotypic synonym *Spermophilus citellus.* Similarly, *Haemogamasus nidiformes* to *Haemogamasus nidiformis,* |
| Dahesh and Mikhail [28] | Egypt; 2016 | *Rattus norvegicus* (N=41)  *Rattus rattus* (N=44)  R=85 (f=41, m=44), Rf=40 | **Fleas** (FiR=52.94%)  *Leptopsylla segnis*  *Xenopsylla cheopis* | Most of the fleas were *Xenopsylla cheopis* |  |
| Darvishi et al. [29] | Iran; 2011 | *Mus musculus* (F=3, M=2)  R=5, Rf=2 | **Fleas** (F=15, FiR=40%, FI=3)  *Leptopsylla aethiopicus aethiopicus* |  | Spelling is corrected from *muscuuls* to *musculus* |
| El Bahrawy and al Dakhil [30] | KSA | *Acomys dimidiatus*  *Gerbillus* spp.  *Jaculus jaculus*  *Meriones* sp*.*  *Mus musculus*  *Rattus norvegicus*  *Rattus rattus*  R=88 | **Fleas** (F=140, FI=1.59)  *Ctenocephalides felis* (N=12)  *Xenopsylla cheopis* (N=128)  **Lice** (L=73, LI=0.9)  *Polyplax spinulosa* | The highest flea infestation was in *Rattus rattus* (68.1%), followed by *Rattus norvegicus* (26.9%), and *Mus musculus* (11.1%). Female rodents were more infested than males. |  |
| El Kady et al. [31] | Egypt | *Rattus norvegicus* (N=9)  *Rattus rattus alexandrines* (N=9)  *Rattus rattus frugivorous* (N=13)  R=31 | **Mites** (M=118, MI=3.81%)  *Dermanyssus gallinae* (N=15)  *Eulaelaps stabularis* (N=4)  *Laelaps nuttalli* (N=84)  *Ornithonyssus bacoti* (N=15) |  |  |
| El Kady et al. [32] | Egypt; 2006-07 | *Mus musculus* (N=15)  *Rattus norvegicus* (N=33)  *Rattus rattus alexandrines* (N=48)  *Rattus rattus frugivorous* (N=39)  R=135 (f=66, m=69) | **Fleas** (F=114, FI=0.84)  *Ctenocephalides canis* (N=16)  *Pulex irritans* (N=2)  *Xenopsylla cheopis* (N=96)  **Mites** (M=165, MI=1.2)  *Dermanyssus gallinae* (N=18)  *Laelaps nuttalli* (N=96)  *Ornithonyssus bacoti* (N=51)  **Lice** (L=93, LI=0.69)  *Polyplax spinulosa* (N=93)  **Ticks** (T=16, TI=0.12)  *Hyalomma* sp (N=2)  *Rhipicephalus* sp (N=14) | Rat index was maximal in spring, followed by summer, autumn, and winter. The ectoparasite abundance depends on the host species. The highest index was on *Rattus norvegicus,* followed by *Rattus rattus*, and *Mus musculus.* |  |
| El-Bahrawy and al-Dakhil [33] | KSA | *Acomys dimidiatus*  *Gerbillus* spp.  *Jaculus jaculus*  *Meriones* sp.  *Mus musculus*  *Rattus norvegicus*  *Rattus rattus* | **Mites** (M=321)  *Articholaelaps glasgowi* (N=3)  *Cheyletus eruditus* (N=2)  *Laelaps nuttalli* (N=234)  *Ornithonyssus bacoti* (N=82)  **Ticks** (T=57)  *Hyalomma* spp. (N=2)  *Rhipicephalus* spp. (N=55) | Abundance of rodents differs in urban and semi-aired regions. Ectoparasites infestation varies with rodent species and their abundance. | Spelling is corrected from *Orithonysus* to *Ornithonyssus* |
| El-Kady et al. [34] | Egypt; 1997-98 | *Acomys cahirinus dimidiatus* (N=56)  *Acomys russatus russatus* (N=6)  *Dipodillus dasyurus dasyurus* (N=21)  *Eliomys quercinus melanurus (*N=3)  *Sekeetamys calurus calurus (*N=2)  R=88 | **Fleas** (F=49, FI=0.56)  *Nosopsyllus penicus geneatus* (N=2)  *Nosopsyllus gerbillophilus theodori* (N=9)  *Parapulex chephrenis (*N=5)  *Xenopsylla cleopatrae (*N=4)  *Xenopsylla dipodill* (N=16)  *Xenopsylla compformis* (N=13)  **Lice** (L=72, LI=0.82)  *Polyplax spinulosa* (N=72)  **Mites** (M=112, MI=1.27)  *Haemolaelaps glasgowi* (N=13)  *Cheyletiella* spp. (N=2)  *Laelaps nuttalli* (N=15)  *Dermanyssus sanguineus (*N=54)  *Ornithonyssus bacoti* (N=14)  *Ornithonyssus sylviarum* (N=14) |  | *Dermanyssus sanguineus* (synonymous with *Liponyssoides sanguineus*) and *Xenopsylla cleopatrae* were formerly known as *Allodermanyssus* *sanguineus* and *Synosternus cleopatrae* respectively. The spelling *chephrensis* is corrected to *chephrenis* |
| El-Kammah et al. [35] | Egypt | *Gerbillus pyramidum*  R=95 ; (f=55, m=10), uk=30 | **Mites**  *Laelaps sinai* |  | Spelling is corrected from *Laesaps* to *Laelaps* |
| Eslami et al. [36] | Iran | *Rattus rattus*  R=100 (F=52, M=48), Rm=71, Rl=66 | **Lice** (LiR=66%)  *Polyplax spinulosa*  **Mites** (MiR=71%)  *Laelaps nuttalli*  *Dermanyssus muris* | There is no significant relationship between the gender of rodents and ectoparasite species. Geographical location is important for ectoparasite infestation; mangrove forests and villages of islands were highly infested with ectoparasites. | *Dermanyssus muris* is synonymous with *Liponyssoides muris* |
| Farhang-Azad and Neronov [37] | Iran | *Gerbillus cheesmani* (N=5)  *Gerbillus nanus* (N=10)  *Meriones crassus* (N=12)  *Meriones hurrianae* (N=1)  *Meriones libycus* (N=24)  *Meriones meridianus* (N=4)  *Meriones persicus* (N=35)  *Meriones tristrami* (N=7)  *Meriones vinogradovi* (N=13)  *Rhombomys opimus* (N=18)  *Tatera indica* (N=13)  R=142 | **Fleas**  *Coptopsylla bairamalienis*  *Coptopsylla iranica*  *Coptopsylla lamellifer*  *Coptopsylla mesghalii*  *Coptopsylla mofidii*  *Coptopsylla neronovi*  *Ctenophthalmus dolichus*  *Echidnophaga oschanini*  *Nosopsyllus baltazardi*  *Nosopsyllus iranus*  *Nosopsyllus laeviceps*  *Nosopsyllus pringlei*  *Nosopsyllus* sp.  *Nosopsyllus turkmenicus*  *Nosopsyllus vlasovi*  *Nosopsyllus ziarus*  *Paradoxopsyllus grenieri*  *Paradoxopsyllus microphthalmus*  *Rhadinopsylla bivirgis*  *Rhadinopsylla syriaca*  *Rhadinopsylla ucrainica*  *Stenoponia tripectinata*  *Stenoponia vlasovi*  *Xenopsylla buxtoni*  *Xenopsylla cleopatrae*  *Xenopsylla conformis*  *Xenopsylla gerbilli*  *Xenopsylla hussaini*  *Xenopsylla nuttalli*  *Xenopsylla persica* | The presence of a flea depends on the ecology of an area, such as sandy areas and firm soil. | *Xenopsylla cleopatrae* was formerly known as *Synosternus cleopatrae* |
| Gaaboub et al. [38] | Egypt; 1967-77 | *Arvicanthis niloticus* (N=223)  *Mus musculus* (N=88)  *Rattus norvegicus* (N=127)  *Rattus rattus alexandrinus* (N=22)  *Rattus rattus frugivorus* (N=54)  *Rattus rattus rattus* (N=14)  R=528 (f=311, m=217) | **Fleas** (F=2679, FI=5.27)  *Ctenocephalides felis*  *Echidnophaga gallinacea*  *Leptopsylla segnis*  *Nosopsyllus fasciatus*  *Pulex irritans*  *Xenopsylla cheopis*  **Lice**  *Hoplopleura captiosa*  *Polyplax abyssinica*  *Polyplax spinulosa*  **Mites**  *Echinolaelaps echidninus*  *Haemolaelaps* sp.  *Haemolaelaps zulu*  *Laelaps keegani*  *Ornithonyssus bacoti* | There is a close relationship between the abundance of fleas and lice, the abundance of rodent hosts and season of the year. However, no seasonal variation was recorded for mite abundance. | Spelling is corrected from capitosa to captiosa, *echidnina* to *echidninus; Laelaps echidninus* is a synonyme of *Echinolaelaps echidninus* |
| Gaaboub et al. [39] | Egypt; 1979-80 | *Arvicanthis niloticus* (N=6)  *Mus musculus* (N=125)  *Rattus norvegicus* (N=105)  *Rattus rattus frugivorus* (N=2)  R=238 | **Fleas (**F=394, 1.66)  *Ctenocephalides felis felis* (N=5)  *Pulex irritans (*N=15)  *Xenopsylla cheopis (*N=374)  **Lice (**L=637, LI=2.68)  *Hoplopleura captiosa* (N=156)  *Polyplax abyssinica* (N=167)  *Polyplax spinulosa* (N=314)  **Mites (**M=601, MI=2.53)  *Dermanyssus* spp. (N=109)  *Echinolaelaps echidninus (*N=54)  *Haemolaelaps* spp. (N=3)  *Laelaps keegani* (N=186)  *Ornithonyssus bacoti (*N=249) | Lice and fleas showed a certain degree of host-specificity. However, it was not true in the case of mites. | Spelling is corrected from *capitosa* to *captiosa; Androlaelaps* is the modern synonym of *Haemolaelaps* |
| Garrett and Allred [40] | Turkey; 1965-68 | *Apodemus mystacinus* (N=32)  *Apodemus* sp. (N=10)  *Apodemus sylvaticus* (N=117)  *Cricetulus migratorius* (N=3)  *Meriones* sp. (N=7)  *Microtus* sp. (N=24)  *Mus musculus* (N=281)  *Rattus rattus* (N=25)  R=499, Rm=209, | **Mites (**MiR=41.88%)  *Eulaelaps stabularis*  *Haemogamasus kusumotoi*  *Haemogamasus nidiformis*  *Haemolaelaps fahrenholzi*  *Hirstionyssus arcuatus*  *Hypoaspis miles*  *Laelaps agilis*  *Laelaps algericus*  *Laelaps kochi*  *Laelaps longisetosus*  *Laelaps pavlovskyi*  *Myonyssus decumani*  *Myonyssus gigas*  *Ornithonyssus bacoti* | Mites infestation may be host-specific. | Spelling is changed from *nidiformes* to *nidiformis* |
| Gholipoury et al. [41] | Iran; 2012 | *Meriones libycus* (N=4)  *Mus musculus* (N=48)  *Rattus norvegicus* (N=35)  *Rhombomys opimus* (N=4)  R=91, Rf=1, Rm=3, Rt=8 | **Fleas** (FiR=1.1%)  *Nosopsyllus fasciatus*  **Mites** (MiR=3.3%)  *Laelaps nuttalli*  **Ticks** (TiR=8.79%)  *Rhipicephalus* spp*.* | Higher infestation of ectoparasites in males than in females may be explained by the increased physical movement of males or the higher number of males compared to females. | Spelling corrected from *Nosophylla* to *Nosopsyllus* |
| Hamidi and Nassirkhani [42] | Iran; 2017-18 | *Apodemus witherbyi (*N=23)  *Calomyscus elburzensis* (N=14)  *Calomyscus hotsoni* (N=7)  *Cricetulus migratorius (*N=15)  *Ellobius fuscocapillus (*N=6)  *Meriones libycus (*N=21)  *Meriones persicus (*N=27)  *Microtus paradoxus (*N=8)  *Mus musculus (*N=37)  *Nesokia indica (*N=16)  *Rattus norvegicus (*N=11)  *Rattus pyctoris (*N=1)  *Rhombomys opimus (*N=3)  *Scarturus elater (*N=13)  *Spermophilus fulvus (*N=6)  *Tatera indica (*N=9)  R=217 | **Fleas** (F=192, FI=0.91)  Nosopsyllus fasciatus (N=86)  Nosopsyllus iranus (N=36)  Xenopsylla buxtoni (N=9)  Xenopsylla cheopis (N=61)  **Lice** (L=94, LI=0.43)  *Hoplopleura captiosa (*N=43)  *Polyplax asiatica (*N=23)  *Polyplax gerbilli (*N=6)  *Polyplax paradoxa (*N=13)  *Polyplax spinulosa (*N=9) |  | Spelling is corrected from *Hoplorpleura* to *Hoplopleura* |
| Hanafi-Bojd et al. [43] | Iran; 2003-04 | *Mus musculus* (N=3)  *Rattus norvegicus* (N=100)  *Rattus rattus* (N=16)  *Tatera indica* (N=20)  R=139 | **Fleas** (F=97, FI=0.7)  *Xenopsylla buxtoni* (N=97)  **Lice** (L=161, LI=1.16)  *Hoplopleura captiosa* (N=70)  *Polyplax gerbilli* (N=91)  **Mites** (M=253, MI=1.82)  *Dermanyssus americanus* (N=5)  *Dermanyssus sanguineus* (N=2)  *Echinolaelaps echidninus* (N=28)  *Haemolaelaps glasgowi* (N=9)  *Laelaps nuttalli* (N=203)  *Ornithonyssus bacoti* (N=6)  **Ticks** (T=20, TI=0.14)  *Rhipicephalus* spp*.* (N=20) |  | Spelling corrected from *Haemolaeps* to *Haemolaelaps* and *Haplopleura* to *Hoplopleura* |
| Harrison et al. [44] | KSA | *Acomys dimidiatus* (N=37)  *Gerbillus nanus* (N=70)  *Meriones libycus* (N=41)  *Meriones rex* (N=13)  R=161 | **Fleas** (F=413, FI=2.57)  *Nosopsyllus iranus* (N=50)  *Parapulex chephrenis* (N=16)  *Xenopsylla cleopatrae cleopatrae* (N=128)  *Xenopsylla cleopatrae* spp. (N=22)  *Xenopsylla conformis mycerini* (N=168)  *Xenopsylla nubica* (N=25)  *Xenopsylla* spp*.* (N=4)  **Mites** (M=151, MI=0.94)  *Androlaelaps tateronis* (N=148)  *Ornithonyssus* spp (N=3)  **Ticks** (T=207, TI=1.29)  *Hyalomma impeltatum* (N=9)  *Rhipicephalus camicasi* (N=45)  *Rhipicephalus* spp. (N=153) | The abundance of rodents and their ectoparasites varied among localities. | *Xenopsylla cleopatrae* was formerly known as *Synosternus cleopatrae* |
| Hawlena et al. [45] | Israel | *Gerbillus andersoni*  R=147 | **Fleas** (F=62, FI=0.42)  *Xenopsylla cleopatrae*  **Mites** (M=58, MI=0.39)  *Androlaelaps androgynus*  *Androlaelaps centrocarpus*  *Androlaelaps hirsuta*  *Androlaelaps insculptus*  *Androlaelaps marshalli*  **Ticks** (T=53, TI=0.36)  *Hyalomma impeltatum* | There is no age-related difference in the ectoparasites infestation of rodents. | *Xenopsylla cleopatrae* was formerly known as *Synosternus cleopatrae*; *Androlaelaps hirsti* is corrected to *Androlaelaps hirsuta* |
| Hoogstraal and Traub [46] | Egypt; 1963-65 | *Acomys cahirinus*  *Acomys dimidiatus dimidiatus*  *Acomys dimidiatus megalodus*  *Acomys russatus russatus*  *Arvicanthis niloticus niloticus*  *Eliomys melanurus*  *Eliomys quercinus cyrenaica*  *Jaculus jaculus elbaensis*  *Jaculus jaculus jaculus*  *Jaculus jaculus scrluteri*  *Jaculus orientalis orientali*  *Mus musculus praetextus*  *Rattus norvegicus*  *Rattus rattus*  *Scarturus tetradactyla* | **Fleas** (N=3938)  *Ctenocephalides felis* (N=36  *Echidnophaga gallinacea* (N=208  *Hopkinsipsylla occulta* (N=117)  *Leptopsylla segnis* (N=27)  *Mesopsylla tuschkan* (N=575)  *Myoxopsylla laverani* (N=10)  *Nosopsyllus geneatus* (N=22)  *Nosopsyllus henleyi* (N=8)  *Nosopsyllus londiniensis* (N=191)  *Nosopsyllus* sp. (N=7)  *Parapulex chephrenis* (N=315)  *Pulex irritans* (N=1)  *Pulex irritans* (N=23)  *Rhadinopsylla masculana* (N=1)  *Stenoponia tripectinata* (N=21)  *Synosternus pallidus* (N=169)  *Xenopsylla cheopis* (N=1926)  *Xenopsylla cleopatrae* (N=116)  *Xenopsylla nubica* (N=157)  *Xenopsylla ramesis* (N=4)  *Xenopsylla taractes* (N=4) |  | *Scarturus tetradactyla* is synonymus with *Allactaga tetradactyla; Xenopsylla cleopatrae* was formerly known as *Synosternus cleopatrae* |
| Hoogstraal et al. [47] | Egypt; 1965-66 | *Acomys dimidiatus megalodus*  *Acomys russatus aegyptiacus*  R=96, Rt=37 | **Ticks** (T=373, TiR=37.54%)  *Hyalomma rhipicephaloides* |  |  |
| Imam and Salah [48] | Egypt; 1964-65 | *Acomys cahirinus (*N=30)  *Mus musculus (*N=25)  *Rattus norvegicus (*N=23)  *Rattus rattus* (N=51)  R=129 | **Fleas**  *Xenopsylla cheopis* (N=357) | Rodent abundance can change according to the location of trapping Rattus norvegicus were found to be more infested with rodent fleas. Flea abundance may vary according to season of the year. |  |
| Karaer et al. [49] | Turkey; 2009 | *Mesocricetus auratus*  R=1, Rm=1 | **Mites**  *Demodex aurati*  *Demodex criceti* |  |  |
| Keskin and Beaucournu [50] | Turkey | *Apodemus uralensis*  *Microtus guentheri*  *Spalax leucodon* | **Fleas**  *Ctenophthalmus beyzanurae*  *Ctenophthalmus kefelioglui*  *Ctenophthalmus teres* |  | *Spalax leucodon* is the synonym of *Nannospalax leucodon* |
| Keskin et al. [51] | Turkey; 2015-16 | *Apodemus flavicollis* (N=17)  *Cricetulus migratorius* (N=1)  R=18, Rt=5 | **Ticks** (T=6, TiR=27.78%, TI=0.33)  *Ixodes redikorzevi* (N=5)  *Rhipicephalus turanicus* (N=1) |  |  |
| Keskin et al. [52] | Turkey; 2017-18 | *Apodemus agrarius* (N=1)  *Apodemus flavicollis* (N=9)  *Apodemus* sp*.* (N=3)  *Apodemus uralensis* (N=9)  *Apodemus witherbyi* (N=18)  *Chionomys nivalis* (N=4)  *Microtus arvalis* (N=3)  *Microtus guentheri* (N=3)  *Microtus levis* (N=1)  *Microtus subterraneus* (N=4)  *Mus macedonicus* (N=6)  *Mus musculus* (N=1)  *Myodes glareolus* (N=5)  *Spalax leucodon* (N=3)  *Prometheomys schaposchnikowi* (N=2)  *Spalax leucodon* (N=1)  R=73, Rt=5 | **Ticks** (TiR=6.85%)  *Dermacentor marginatus*  *Ixodes laguri*  *Ixodes redikorzevi* |  | *Mus musculus* is the main species of *Mus domesticus* (*Mus musculus domesticus)*;  *Spalax leucodon* is the synonym of *Nannospalax leucodon.* |
| Keskin et al. [53] | Turkey; 2017-19 | *Apodemus agrarius* (N=2)  *Apodemus flavicollis* (N=17)  *Apodemus* sp. (N=2)  *Apodemus sylvaticus* (N=1)  *Apodemus uralensis* (N=7)  *Apodemus witherbyi* (N=16)  *Chionomys nivalis* (N=3)  *Microtus guentheri* (N=3)  *Microtus levis* (N=1)  *Microtus subterraneus* (N=1)  *Mus macedonicus* (N=12)  *Mus musculus* (N=1)  *Myodes glareolus* (N=5)  *Nannospalax xanthodon* (N=3)  *Prometheomys schaposchnikowi* (N=2)  R=76 (f=16, m=41), UK=19 | **Fleas** (F=222, FI=2.92)  *Amphipsylla rossica* (N=3)  *Ctenophthalmus agyrtes* (N=19)  *Ctenophthalmus bifidatus* (N=9)  *Ctenophthalmus coniunctus* (N=6)  *Ctenophthalmus contiger* (N=1)  *Ctenophthalmus contiger* (N=2)  *Ctenophthalmus euxinicus* (N=2)  *Ctenophthalmus fissurus* (N=8)  *Ctenophthalmus fransmiti* (N=1)  *Ctenophthalmus golovi* (N=1)  *Ctenophthalmus hypanis* (N=1)  *Ctenophthalmus inornatus* (N=6)  *Ctenophthalmus proximus* (N=17)  *Ctenophthalmus reconditus* (N=10)  *Ctenophthalmus secundus* (N=64)  *Ctenophthalmus stirps* (N=3)  *Ctenophthalmus teres* (N=1)  *Frontopsylla elata* (N=2)  *Hystrichopsylla orientalis* (N=2)  *Leptopsylla taschenbergi* (N=29)  *Megabothris turbidus* (N=19)  *Nosopsyllus consimilis* (N=1)  *Nosopsyllus durii* (N=7)  *Nosopsyllus sarinus* (N=2)  *Palaeopsylla incisa* (N=1)  *Rhadinopsylla pentacantha* (N=1)  *Stenoponia tripectinata* (N=4) |  |  |
| Khajeh et al. [54] | Iran; 2014-15 | *Acomys dimidiatus* (N=21)  *Apodemus witherbyi* (N=6)  *Calomyscus hotsoni* (N=6)  *Cricetulus migratorius* (N=1)  *Gerbillus nanus* (N=13)  *Golunda ellioti* (N=2)  *Jaculus blanfordi* (N=7)  *Meriones libycus* (N=13)  *Microtus mystacinus kermanesis* (N=1)  *Mus musculus* (N=36)  *Nesokia indica* (N=8)  *Rattus rattus* (N=2)  *Tatera indica* (N=30)  R=146 | **Fleas** (F=236, FI=1.62)  *Amphipsylla* spp*.* (N=1)  *Nosopsyllus medus* (N=20)  *Xenopsylla buxtoni* (N=30)  *Xenopsylla cheopis* (N=74)  *Xenopsylla conformis* (N=26)  *Xenopsylla gerbilli* (N=85)  **Lice** (L=343, LI=2.35)  *Hoplopleura* spp*.* (N=181)  *Polyplax* spp*.* (N=162)  **Mites** (M=70, MI=0.48)  *Ornithonyssus bacoti* (N=70)  **Ticks** (T=32, TI=0.22)  *Hyalomma* spp*.* (N=8)  *Rhipicephalus* spp*.* (N=24) | Ectoparasites infestation differs with rodent species. | Spelling is corrected from *Amphipsylia* to *Amphipsylla* and *Haplopleura* to *Hoplopleura* |
| Kia et al. [55] | Iran; 2007 | Hamster (N=1)  *Mus musculus* (N=6)  *Rattus norvegicus* (N=57)  *Rattus rattus* (N=13)  R=77 | **Fleas** (F=60, FI=0.78)  *Xenopsylla astia*  *Xenopsylla cheopis*  **Lice** (L=1, LI=0.01)  *Polyplax spinulosa*  **Mites** (M=6, MI=0.08)  *Laelaps nuttalli*  **Ticks** (T=2, TI=0.03)  *Hyalomma* sp*.*  *Rhipicephalus* sp*.* | Different types of ectoparasites had different frequency of infestation on rodents. Fleas were more frequent than lice and ticks. The most abundant ectoparasite was *Xenopsylla* on *Rattus norvegicus.* |  |
| Kim and Emerson [56] | Iran | *Acomys dimidiatus*  *Apodemus sp.*  *Apodemus sylvaticus*  *Calomyscus bailwardi*  *Clethrionomys sp.*  *Gerbillus cheesmani*  *Gerbillus nanus*  *Jaculus blanfordi*  *Meriones crassus*  *Meriones libycus*  *Meriones persicus*  *Microtus sp.*  *Chionomys nivalis*  *Mus musculus*  *Mus spicilegus*  *Nesokia indica*  *Tatera indica* | **Lice**  *Eulinognathus aculeatus*  *Eulinognathus aculeatus*  *Hoplopleura acanthopus*  *Hoplopleura acanthopus*  *Hoplopleura acanthopus*  *Hoplopleura acanthopus*  *Hoplopleura affinis*  *Hoplopleura affinis*  *Hoplopleura captiosa*  *Hoplopleura captiosa*  *Hoplopleura longula*  *Hoplopleura longula*  *Hoplopleura meridionidis*  *Hoplopleura meridionidis*  *Polyplax asiatica*  *Polyplax asiatica*  *Polyplax brachyrrhyncha*  *Polyplax brachyrrhyncha*  *Polyplax calomysci*  *Polyplax gerbilli*  *Polyplax kaiseri*  *Polyplax paradoxa*  *Polyplax reclinata*  *Polyplax serrata*  *Polyplax stephensi* |  | Spelling is corrected from *specilegus* to *spicilegus, blandfordi* to *blanfordi, merionidis* to *meridionidis.*  *Chionomys nivalis* was previously known as *Microtus nivalis* |
| Krasnov et al. [57] | Israel; 1992-95 | *Meriones crassus*  R=392 | **Fleas**  *Nosopsyllus theodori*  *Xenopsylla conformis*  *Xenopsylla dipodill*  **Ticks**  *Rhipicephalus sanguineus*  *Hyalomma savignyi* | Rodent habits changed with seasonal and environmental variation. Rodent changes their burrow that may be a cause of getting infested with ectoparasites. Fleas are the most frequent ectoparasites; *Xenopsylla conformis* was the most frequent flea on *Meriones crassus.* |  |
| Krasnov et al. [58] | Israel; 1992-95 | *Acomys cahirinus* (N=60)  *Acomys russatus* (N=35)  *Eliomys melanurus* (N=16)  *Gerbillus dasyurus* (N=423)  *Gerbillus gerbillus* (N=36)  *Gerbillus henleyi* (N=155)  *Gerbillus nanus* (N=1)  *Jaculus jaculus* (N=26)  *Meriones crassus* (N=151)  *Mus musculus* (N=92)  *Psammomys obesus* (N=6)  *Sekeetamys calurus* (N=32)  R=1033 | **Fleas** (F=3702, FI=3.58)  *Coptopsylla africana* (N=6)  *Leptopsylla algira costai* (N=1)  *Myoxopsylla laverani traubi* (N=68)  *Nosopsyllus theodori* (N=477)  *Parapulex chephrenis* (N=288)  *Rhadinopsylla masculana* (N=20)  *Stenoponia tripectinata medialis* (N=100)  *Xenopsylla cheopis* (N=3)  *Xenopsylla conformis mycerini* (N=1655)  *Xenopsylla dipodill* (N=727)  *Xenopsylla ramesis* (N=357) | No significant sex and age differences were found, neither for rodent nor flea abundance. No significant variation between seasons was found in the prevalence or intensity of overall infestation in any host species, except the winter fleas. The intensity of winter fleas was relatively high in *M. crassus* and *G. dasyurus*. Flea abundance can vary with rodent host habitat abundance and distribution. | Spelling is corrected from *chephrensis* to *chephrenis* |
| Krasnov et al. [59] | Israel; 1992-95 | *Gerbillus dasyurus*  *Meriones crassus*  R=574 | **Fleas** (N=3075, FI=5.36)  *Coptopsylla africana*  *Nosopsyllus theodori*  *Parapulex chephrenis*  *Rhadinopsylla masculana*  *Stenoponia tripectinata medialis*  *Xenopsylla conformis*  *Xenopsylla dipodilli*  *Xenopsylla ramesis* | Flea abundance is influenced affected by host species. Flea abundance can also be affected by the environment and location of sampling. |  |
| Krasnov et al. [60] | Israel; 1992-98 | *Acomys cahirinus*  *Acomys russatus*  *Eliomys melanurus*  *Gerbillus andersoni*  *Gerbillus dasyurus*  *Gerbillus gerbillus*  *Gerbillus henleyi*  *Gerbillus nanus*  *Gerbillus pyramidum*  *Jaculus jaculus*  *Meriones crassus*  *Mus musculus*  *Psammomys obesus*  *Sekeetamys calurus*  R=1767, Rf=858 | **Fleas** (F=5722, FiR=48.56%, FI=3.24)  *Coptopsylla africana*  *Leptopsylla algira costai*  *Myoxopsylla laverani*  *Nosopsyllus iranus theodori*  *Ophthalmopsylla volgensis palestinica*  *Parapulex chephrenis*  *Rhadinopsylla masculana*  *Stenoponia tripectinata acmaea*  *Stenoponia tripectinata medialis*  *Xenopsylla cheopis*  *Xenopsylla cleopatrae*  *Xenopsylla conformis mycerini*  *Xenopsylla dipodilli*  *Xenopsylla ramesis* | *Nosopsyllus iranus, Rhadinopsylla masculine,* and *Stenoponia tripectinata* are winter fleas, active 3-4 months in cool-season ; Others are active all around the year. No characteristic frequency distribution was detected for fleas on rodents. | Spelling is corrected from *Masculina and chephrensis* to *Masculana* and *chephrenis* respectively*; Xenopsylla cleopatrae* was formerly known as *Synosternus cleopatrae.* |
| Krasnov et al. [61] | Israel; 2000-01 | *Acomys cahirinus* (N=12)  *Eliomys melanurus* (N=2)  *Gerbillus dasyurus* (N=182)  *Jaculus jaculus* (N=2)  *Meriones crassus* (N=41)  *Mus musculus* (N=15)  R=254 | **Fleas** (F=1055, FI=4.15)  *Coptopsylla africana (*N=4)  *Myoxopsylla laverani (*N=2)  *Nosopsyllus iranus theodori* (N=186)  *Ophthalmopsylla volgensis palestinica (*N=2)  *Parapulex chephrenis* (N=46)  *Rhadinopsylla masculana (*N=7)  *Stenoponia tripectinata medialis* (N=161)  *Xenopsylla dipodilli* (N=356)  *Xenopsylla ramesis* (N=291) | Flea reproduction is influenced by seasons of the year. | The spelling *chephrensis* is corrected to *chephrenis* |
| Krasnov et al. [62] | Israel; 1998-2002 | *Gerbillus dasyurus*  *Meriones crassus* | **Fleas**  *Nosopsyllus iranus theodori*  *Stenoponia tripectinata medialis*  *Stenoponia tripectinata pyramidis*  *Xenopsylla conformis*  *Xenopsylla ramesis* |  |  |
| Krasnov et al. [63] | Israel; 1992-2004 | *Acomys cahirinus*  *Acomys russatus*  *Eliomys melanurus*  *Gerbillus andersoni allenbyi*  *Gerbillus dasyurus*  *Gerbillus gerbillus*  *Gerbillus henleyi*  *Gerbillus nanus*  *Gerbillus pyramidum*  *Jaculus jaculus*  *Meriones crassus*  *Mus musculus*  *Psammomys obesus*  *Sekeetamys calurus* | **Fleas**  *Coptopsylla africana*  *Leptopsylla algira costai*  *Myoxopsylla laverani traubi*  *Nosopsyllus iranus theodori*  *Nosopsyllus pumilionis*  *Ophthalmopsylla volgensis*  *Parapulex chephrenis*  *Rhadinopsylla masculana*  *Stenoponia tripectinata medialis*  *Xenopsylla cheopis*  *Xenopsylla cleopatrae pyramidis*  *Xenopsylla conformis mycerini*  *Xenopsylla dipodilli*  *Xenopsylla ramesis* | Some fleas were host-specific (e.g., *Parapulex chephrenis*, and others are host opportunistic (e.g., *Xenopsylla dipodilli*. Some are seasonally active only (e.g., *Rhadinopsylla masculana* | *Xenopsylla cleopatrae* was formerly known as *Synosternus cleopatrae* |
| Krasnov et al. [64] | Israel | *Acomys cahirinus* (N=55)  *Dipodillus dasyurus* (N=73)  *Gerbillus andersoni* (N=579)  *Meriones crassus* (N=180)  R=887 | **Fleas** (F=18269, FI=20.6)  *Nosopsyllus iranus* (N=1037)  *Parapulex chephrenis* (N=784)  *Stenoponia tripectinata* (N=472)  *Xenopsylla cleopatrae* (N=9684)  *Xenopsylla conformis* (N=3106)  *Xenopsylla dipodilli* (N=1623)  *Xenopsylla ramesis* (N=1563) | Rodent gender and age did not correspond with the number of fleas. It is sometimes female-biased and sometimes with males. | *Xenopsylla cleopatrae* was formerly known as *Synosternus cleopatrae*; The spelling *chephrensis* is corrected to *chephrenis* |
| Lehmann-a [65] | Israel | *Gerbillus andersoni allenbyi* | **Fleas**  *Stenoponia tripectinata*  *Xenopsylla cleopatrae*  **Lice**  *Polyplax gerbilli*  **Mites**  *Androlaelaps centrocarpus*  *Androlaelaps hirsuta*  *Androlaelaps insculptus*  *Androlaelaps marshalli*  *Hirstionyssus carticulatus*  **Ticks**  *Rhipicephalus sanguineus* | Fleas were more common than the other ectoparasites. *Rhipicephalus sanguineus* ticks were available in all stages of gerbils but the immature stage of the tick was more in adult gerbils. The presence of ectoparasites can cause anemia to the rodent hosts. Ectoparasites can impact the Gerbils population. | *Xenopsylla cleopatrae* was formerly known as *Synosternus cleopatrae;* The spelling is corrected from *hirsti* to *hirsuta* |
| Lehmann-b [66] | Israel | *Gerbillus andersoni allenbyi* | **Fleas**  *Xenopsylla cleopatrae* | Flea abundance was influenced by the gender of rodent hosts, male rats contain more fleas than females. | *Xenopsylla cleopatrae* was formerly known as *Synosternus cleopatrae* |
| Lewis [67] | Lebanon | *Apodemus mystacinus mystacinus*  *Apodemus sylvaticus*  *Cricetulus migratorius cinerascens*  *Meriones nivalis*  *Meriones tristrami*  *Microtus guentheri philistinus*  *Rattus norvegicus*  *Rattus rattus alexandrinus*  *Sciurus anomalus syriacus* | **Fleas**  *Callopsylla caspia*  *Ceratophyllus hirundinis*  *Ctenocephalides felis felis*  *Ctenophthalmus congener*  *Echidnophaga murina*  *Leptopsylla segnis*  *Leptopsylla taschenbergi taschenbergi*  *Nosopsyllus durii*  *Nosopsyllus iranus*  *Stenoponia tripectinata spinellosa*  *Xenopsylla cheopis*  *Xenopsylla ramesis* |  | *Pulex segnis* is the basionym of *Leptopsylla segnis* |
| Lewis [68] | Egypt | *Eliomys quercinus cyrenaicus*  *Jaculus orientalis*  *Meriones crassus* | **Fleas**  *Echidnophaga murina*  *Myoxopsylla laverani traubi* |  | Spelling is corrected from *murin* to *murina* |
| Loftis et al. [69] | Egypt; 2002-03 | *Mus musculus* (N=1)  *Rattus norvegicus* (N=98)  *Rattus rattus* (N=114)  R=213, Rf=213 | **Fleas** (F=953, FiR=100%, FI=4.47)  *Ctenocephalides felis* (N=11)  *Echidnophaga gallinacea* (N=12)  *Leptopsylla segnis* (N=37)  *Xenopsylla cheopis* (N=853) | *Xenopsylla cheopis* were the most frequent rodent fleas. However, *Rickettsia* sp. was detected in *Echidnophaga gallinacea,* *Leptopsylla segnis,* and *Xenopsylla cheopis.* *Bartonella* sp. was identified in *Xenopsylla cheopis* and *Leptopsylla segnis*. *Coxiella burnetii* was found in *Ctenocephalides felis* and *Xenopsylla cheopis.* |  |
| Mahdi and Arafa [70] | Egypt | *Mus musculus* (R=165 ; f=80, m=85) | **Fleas**  *Xenopsylla cheopis* | Rodent infestation with fleas varies according to the season of the year; it is the highest in summer. |  |
| Mikhail et al. [71] | Egypt; 2009 | *Mus musculus* (N=147)  *Rattus norvegicus*  *Rattus rattus alexandrines* (N=137)  *Rattus rattus frugivorous*  R=286. Rm=281, Rt=284, Rl=281 | **Lice** (L=2999, LiR=98.25%, LI=10.49)  *Polyplax spinulosa*  **Mites** (M=1573, MiR=98.25%, MI=5.5)  *Dermanyssus sanguineus* (N=710)  *Haemolaelaps glasgowi (*N=362)  *Laelaps nuttalli (*N=9)  *Myobia* sp*.* (N=35)  *Ornithonyssus bacoti (*N=424)  *Radfordia* sp*.* (N=33)  **Ticks** (T=365, TiR=99.3%, TI=1.28)  *Hyalomma* sp*.* (N=122)  *Rhipicephalus* sp*.* (N=243) | Flea richness depended on rodent species and the body mass of rodent hosts. Flea richness was not related to rodent habits, movement, size or geographical range. | Spelling corrected from *Orithonysus* to *Ornithonyssus* |
| Mohammadi et al. [72] | Iran; 2012-13 | *Apodemus flavicollis* (N=8)  *Apodemus mystacinus (*N=3)  *Apodemus ponticus* (N=18)  *Apodemus witherbyi (*N=71)  *Dryomys nitedula* (N=12)  *Meriones libycus* (N=2)  *Meriones persicus* (N=18)  *Meriones tristrami* (N=1)  *Meriones vinogradovi (*N=2)  *Microtus qazvinensis (*N=36)  *Microtus socialis* (N=19)  *Mus macedonicus* (N=15)  *Mus musculus* (N=1)  *Scarturus migratorius* (N=1)  *Sciurus anomalus* (N=1)  R=208 | **Fleas** (N=198, FI=1.5)  *Amphipsylla rossica rossica* (N=9)  *Ctenophthalmus iranus persicus* (N=14)  *Ctenophthalmus rettigi smiti* (N=1)  *Ctenophthalmus sp.* (N=2)  *Ctenophyllus rufescens* (N=2)  *Leptopsylla segnis* (N=.7)  *Nosopsyllus iranus iranus* (N=2)  *Paraceras melis melis* (N=20)  *Paraceras sp.* (N=6)  *Paradoxopsyllus microphthalmus* (N=3)  *Xenopsylla buxtoni* (N=123)  *Xenopsylla* sp. (N=9)  **Lice** (N=2, LI=0.1)  *Neohaematopinus* spp.  **Mites** (M=61, MI=0.29)  *Dermanyssus* spp.  *Echinolaelaps echidninus*  *Eulaelaps stabularis*  *Haemaphysalis* spp.  *Haemolaelaps glasgowi*  *Hyalomma* spp.  *Laelaps nuttalli*  *Macrocheles* spp.  *Rhipicephalus* spp.  **Ticks** (T=51, TI=0.25) | Seasonal variation did not have a significant impact on flea abundance. | The spelling *melio* is corrected to *melis* |
| Moravvej et al. [73] | Iran; 2013-14 | *Apodemus witherbyi* (N=13)  *Cricetulus migratorius* (N=17)  *Ellobius fuscocapillus* (N=16)  *Meriones libycus* (N=14)  *Meriones persicus* (N=19)  *Microtus transcaspicus* (N=20)  *Mus musculus* (N=26)  *Nesokia indica* (N=16)  *Rattus norvegicus* (N=19)  *Spermophilus fulvus* (N=23)  *Tatera indica* (N=14)  R=197, Rf=37, Rm=45, Rt=37, Rl=20 | **Fleas** (FiR=18.78%)  *Nosopsyllus fasciatus*  *Nosopsyllus* spp.  *Xenopsylla cheopis*  *Xenopsylla* spp*.*  **Lice** (LiR=10.15%)  *Hoplopleura captiosa*  *Polyplax asiatica*  **Mites** (MiR=22.84%)  *Haemolaelaps* spp*.*  *Hirstionyssus* spp.  *Laelaps* spp.  **Ticks** (TiR=18.78%)  *Haemaphysalis punctata*  *Haemaphysalis* spp.  *Ixodes* spp.  *Ixodes trianguliceps* | The prevalence of ectoparasites could be influenced by rodent host species. | Spelling is corrected from *punctate* to *punctate* |
| Morick et al. [74] | Israel; 2002-04 | *Acomys cahirinus* (N=4)  *Apodemus sylvaticus* (N=1)  *Microtus socialis* (N=3)  *Mus musculus* (N=25)  *Rattus rattus* (N=79)  R=112 | **Fleas** (F=135, FI=1.21)  *Pulex irritans (*N=1)  *Xenopsylla cheopis* (N=134)  **Lice** (L=3, LI=0.03)  *Anoplura*  **Mites** (M=9, MI=0.08)  *Gamasina*  **Ticks** (T=9, TI=0.08)  *Haemaphysalis* sp*.* (N=5)  Non identified *ixodid* (N=4) |  |  |
| Morick et al. [75] | Israel; 2007 | *Dipodillus dasyurus* (N=3)  *Gerbillus andersoni allenbyi* (N=63)  *Gerbillus pyramidum* (N=7)  *Meriones sacramenti* (N=5)  *Meriones tristrami* (N=33)  *Mus musculus* (N=11)  R=122 | **Fleas** (F=1148, FI=9.41)  *Leptopsylla algira*  *Xenopsylla cleopatrae*  *Xenopsylla ramesis* |  | *Xenopsylla cleopatrae* was formerly known as *Synosternus cleopatrae* |
| Morsy et al. [76] | Egypt; 1981 | *Mus musculus* (N=50)  *Rattus norvegicus* (N=980)  *Rattus rattus* (N=344)  R=1374, Rf=343, Rm=18, Rt=55, Rl=17 | **Fleas** (F=594, FiR=24.96%, FI=0.43)  *Ctenocephalides felis*  *Leptopsylla segnis*  *Pulex irritans linnaeus*  *Xenopsylla cheopis*  **Lice** (L=30, LiR=1.24%, LI=0.02)  *Mallophaga*  **Mites** (M=43, MiR=1.31%, MI=0.03)  *Dermanyssus sanguineus*  *Ornithonyssus* spp.  **Ticks** (T=80, TiR=4%, TI=0.06)  *Hyalomma excavatum*  *Rhipicephalus sanguineus* |  | *Liponyssoides sanguineus* (synonymus with *Dermanyssus sanguineus*) is the modern synonym of *Allodermanyssus sanguineus; Leptopsylla segnis* is the alternative scientific name of *Ctenopsyllus segnis* |
| Morsy et al. [77] | Egypt | *Acomys cahirinus* (N=34)  *Mus musculus praetextus* (N=169)  *Rattus norvegicus* (N=240)  *Rattus rattus* (N=229)  *Sekeetamys calurus* (N=4)  R=676, Rf=482, Rt=39, Rl=11 | **Fleas** (F=510, FiR=71.3%, FI=0.75)  *Ctenocephalides felis* (N=60)  *Echidnophaga gallinacea* (N=21)  *Leptopsylla segnis* (N=4)  *Pulex irritans* (N=26)  *Xenopsylla cheopis* (N=399)  **Lice** (L=30, LiR=1.63%, LI=0.04)  *Polyplax spinulosa*  **Ticks** (T=41, TiR=5.77%, TI=0.06)  *Hyalomma* spp.  *Rhipicephalus sanguineus* | Flea index varied with the season, highest in April and lowest in January and February. | *Leptopsylla segnis* is the scientific alternative name of *Ctenopsyllus segnis* |
| Morsy et al. [78] | Egypt; 1984-85 | *Mus musculus* (N=230)  *Rattus norvegicus* (N=562)  *Rattus rattus alexandrines* (N=325)  R=1117, Rf=109 | **Fleas** (F=151, FiR=9.76%, FI=0.14)  *Ctenocephalides felis* (N=7)  *Leptopsylla segnis* (N=17)  *Pulex irritans* (N=4)  *Xenopsylla cheopis* (N=123) | Flea index and infestation rates were directly related to rodent host’s body size and rodent species. Female fleas were more numerous than males. Season affects rodent abundance; rodents increased in summer and autumn and decreased in winter and spring. |  |
| Morsy et al. [79] | Egypt, 2001 | *Meriones rex* (R=25 ; f=16, m=9 | **Fleas** (F=55, FI=2.2)  *Ctenocephalides arabicus* (N=40)  *Xenopsylla astia* (N=15)  **Mites** (M=16, MI=0.64)  *Ornithonyssus bacoti* (N=16)  **Ticks** (T=6, TI=0.24)  Tick nymphs (N=6) |  |  |
| Mostafavi et al. [80] | Iran | *Apodemus flavicollis* (N=8)  *Apodemus sp.* (N=22)  *Apodemus witherbyi* (N=69)  *Calomyscus sp.* (N=10)  *Cricetulus migratorius* (N=2)  *Dryomys nitedula* (N=15)  *Meriones persicus* (N=30)  *Meriones sp.* (N=29)  *Mus macedonicus* (N=59)  *Mus musculus* (N=1)  R=245 | **Fleas** (F=153, FI=0.62)  *Ctenophthalmus iranus persicus* (N=11)  *Ctenophthalmus rettigi smiti* (N=1)  *Leptopsylla segnis* (N=6)  *Paraceras melis melis* (N=11)  *Paradoxopsyllus microphthalmus* (N=1)  *Xenopsylla buxtoni* (N=123)  **Mites** (M=37, MI=0.15)  *Dermanyssus sanguineous* (N=1)  *Echinolaelaps echidninus (N=2*)  *Eulaelaps stabularis* (N=15)  *Haemolaelaps glasgowi* (N=13)  *Laelaps nuttalli* (N=6)  **Ticks** (T=54, TI=0.22)  *Haemaphysalis* spp*.* (N=35)  *Hyalomma* spp*.* (N=19) | The density of fleas on the body of *Meriones persicus* was much higher than on other rodents; | Spelling is corrected from *microphtalmus* to *microphthalmos* and *sanguineous to sanguineus* |
| Mumcuoglu et al. [81] | Israel; 1989-91 | *Crocidura russula* (N=2)  *Meriones crassus* (N=21)  *Mus musculus* (N=115)  *Rattus rattus* (N=13)  R=111 | **Ticks**  *Rhipicephalus sanguineus*  *Rhipicephalus turanicus* | Ticks abundance varied with the season of the year (higher during April to October and positively correlated with environmental temperature, and location of sampling. |  |
| Mumcuoglu et al. [82] | Israel | *Rattus norvegicus*  R=35, Rf=24 | **Fleas** (F=323, FiR=68.57%, FI=9.23)  *Echidnophaga murina* (N=191)  *Xenopsylla cheopis* (N=132) | There was a correlation between the density of animals, and their ectoparasites |  |
| Nasereddin et al. [83] | Israel; 2011-12 | Rat  R=7, Rf=5 | **Fleas** (F=7, FiR=71.43%, FI=1)  *Xenopsylla* sp*.* |  |  |
| Nateghpour et al. [84] | Iran; 2008-09 | *Gerbillus nanus* (N=3)  *Meriones hurrianae* (N=25)  *Meriones libycus* (N=2)  *Tatera indica* (N=37)  R=67 | **Fleas** (F=127, FI=1.9)  *Xenopsylla astia* (N=104)  *Xenopsylla conformis* (N=2)  *Xenopsylla hutoni* (N=4)  *Xenopsylla nubica* (N=17)  **Ticks** (T=24, TI=0.36)  *Boophilus* sp.(N=3)  *Hyalomma* spp. (N=14)  *Rhipicephalus* spp. *(*N=7)  **Mites** (M=299, MI=4.46)  *Androlaelaps hermaphrodita* (N=168)  *Eulaelaps* spp*.* (N=39)  *Haemogamasus* (N=19)  *Laelaps ciccuminata* (N=56)  *Paracheylaelaps pyriformis* (N=17)  **Lice** (L=972, LI=14.51)  *Polyplax spinulosa* | Migration habits of rodents may affect the spatial distribution of ectoparasites and their transmitted pathogens. |  |
| Oyoun et al., [85] | Egypt | *Gerbillus pyramidum* | **Mites**  *Listrophoridus arishi* (N=1119) |  |  |
| Pourhossein et al. [86] | Iran; 2013 | *Tatera indica*  R=9 | **Fleas** (F=48, FI=5.33)  *Xenopsylla* spp.  **Ticks** (T=10, TI=1.11)  *Hyalomma* spp. | Migration of rodents increases the risk of spreading pathogens in different populations. |  |
| Psaroulaki et al. [87] | Cyprus; 2001-03 | *Mus musculus* (N=3)  *Rattus norvegicus* (N=402)  *Rattus rattus frugivorous* (N=220)  R=625, Rf=243 | **Fleas** (F=1035, FiR=38.88%, FI=1.66)  *Ctenocephalides felis* |  |  |
| Psaroulaki et al. [88] | Cyprus; 2000-03 | *Rattus norvegicus* (N=402)  *Rattus rattus frugivorous* (N=220)  R=622 (f=322, m=293), uk=7, Rf=252 | **Fleas** (F=287, FiR=40.51%, FI=0.46)  *Ctenocephalides canis* (N=2)  *Ctenocephalides felis* (N=75)  *Leptopsylla segnis* (N=17)  *Nosopsyllus fasciatus* (N=4)  *Xenopsylla cheopis* (N=189)  **Ticks** (T=3, TI=0.01)  Unspecified ticks | Rodent capture varied according to season, mostly caught during the summer. Rodents were mostly parasitized by fleas. |  |
| Psaroulaki et al. [89] | Cyprus; 2000-06 | *Rattus norvegicus*  *Rattus rattus*  R=622, Rf=252 | **Fleas** (F=983, FiR=40.51%, FI=1.58)  *Ctenocephalides canis* (N=5)  *Ctenocephalides felis* (N=250)  *Xenopsylla cheopis* (N=728) |  |  |
| Rahdar et al. [90] | Iran; 2009-10 | *Mus musculus* (N=4)  *Rattus norvegicus* (N=20)  *Scarturus* sp*.* (N=1)  *Tatera indica* (N=5)  R=30 | **Fleas**  *Xenopsylla* sp*.*  **Ticks**  *Haemaphysalis* sp*.*  **Mites**  *Dermanyssus sanguineus*  *Ornithonyssus* sp*.*  **Lice**  *Eulinognathus* sp*.*  *Polyplax spinulosa*  *Polyplax stephensi* |  | *Scarturus* is synonymous to *Alactaga* (correct spelling is *Allactaga)*. *Dermanyssus sanguineus*  is synonymous with *Liponyssoides sanguineus.* Spellings has been corrected from *Polypolax* to *Polyplax and Hemaphysalis* to *Haemaphysalis.* |
| Reeves et al. [91] | Egypt; 2002-03 | *Rattus norvegicus*  *Rattus rattus* | **Lice** (L=1023)  *Hoplopleura pacifica*  *Pediculus humanus*  *Polyplax spinulosa* |  |  |
| Reeves et al. [92] | Egypt; 2002-03 | *Rattus norvegicus*  *Rattus rattus* | **Mites** (M=616)  *Ornithonyssus bacoti* |  | Spelling is corrected from *Orithonysus* to *Ornithonyssus* |
| Rifaat et al. [93] | Egypt | *Rattus norvegicus*  R=6788, Rf=5100 | **Fleas** (F=5807, FI=0.86, FiR=75.13%)  *Xenopsylla* spp. | Season of the year did not have any significant effect on rodent flea abundance. |  |
| Rzotkiewicz et al. [94] | Israel; 2007-09 | *Gerbillus andersoni allenbyi*  *Gerbillus dasyurus*  *Gerbillus pyramidum*  *Meriones crassus*  *Meriones sacramenti*  *Meriones tristrami*  *Mus musculus*  R=122 | **Fleas** (F=568, FI=4.66)  *Leptopsylla algira*  *Xenopsylla cleopatrae*  *Xenopsylla ramesis* |  | *Xenopsylla cleopatrae* was formerly known as *Synosternus cleopatrae* |
| Sanborn and Hoogstraal [95] | Yemen; 1951 | *Rattus rattus rattus*  *Meriones rex buryi*  *Arvicanthis niloticus naso*  *Myomys fumatus yemeni*  *Mus musculus bactrianus*  *Acomys dimidiatus homericus*  *Gerbillus cheesmani maritimus*  *Gerbillus famulus* | **Fleas**  *Parapulex chephrenis*  *Xenopsylla* spp.  *Xenopsylla cheopis*  *Xenopsylla cleopatrae*  **Mites**  *Dermanyssus muris*  *Haemolaelaps namrui*  *Laelaps nuttalli*  *Microtrombicula hoogstraali*  *Neoschongastia yemenensis*  *Neotrombicula saperoi*  **Ticks**  *Haemophysalis leachi*  *Hyalomma* sp.  *Ixodes* sp.  *Rhipicephalus simus*  *Rhipiecphalus* sp. |  | Spelling is corrected from *chephrinus* to *chephrenis* and *nutalli to nuttalli. Xenopsylla cleopatrae* was formerly known as *Synosternus cleopatrae* and *Microtrombicula hoogstraali* is synonymous with *Trombicula hoogstraali.* |
| Shamsi et al. [96] | Iran; 2017 | *Apodemus* sp*.*  *Apodemus sylvaticus*  *Arvicola amphibius*  *Calomyscus elburzensis*  *Chionomys nivalis*  *Chionomys* sp.  *Cricetulus migratorius*  *Meriones tamariscinus* | **Mites**  *Brunehaldia iranica*  *Brunehaldia lucida*  *Brunehaldia silvatica*  *Cheladonta afshari*  *Cheladonta firdousii*  *Cheladonta iraniensis*  *Doloisia skljari*  *Euschoengastia meshhedensis*  *Helenicula sparsa*  *Hirsutiella alpina*  *Kepkatrombicula brevis*  *Kepkatrombicula magna*  *Leptotrombidium noxium*  *Leptotrombidium raropinne*  *Leptotrombidium silvaticum*  *Leptrombidium subsilvaticum*  *Microtrombicula traubi*  *Miyatrombicula ramitensis*  *Multisetosa persicus*  *Neotrombicula aideriensis*  *Neotrombicula autumnalis*  *Neotrombicula delijani*  *Neotrombicula elegans*  *Neotrombicula lubrica*  *Neotrombicula monticola*  *Neotrombicula rostrata*  *Neotrombicula talmiensis*  *Neotrombicula tehranensis*  *Neotrombicula turkestanica*  *Neotrombicula vernalis*  *Neotrombicula vulgaris*  *Schoutedenichiaangusta*  *Shunsennia oudemansi*  *Walchia cognata* |  |  |
| Shayan and Rafinejad [97] | Iran; 2002-03 | *Apodemus sylvaticus* (N=28)  *Calomyscus bailwardi* (N=9)  *Cricetulus migratorius* (N=7)  *Ellobius fuscocapillus* (N=15)  *Meriones persicus* (N=64)  *Microtus socialis* (N=16)  *Mus musculus* (N=15)  *Rattus rattus* (N=12)  *Sciurus anomalus* (N=2)  R=168 | **Fleas** (F=16, FI=0.1)  *Nosopsyllus fasciatus* (N=4)  *Nosopsyllus iranus* (N=2)  *Xenopsylla buxtoni* (N=10)  **Lice** (L=7, LI=0.04)  *Neohaematopinus laeviusculus*  **Mites** (M=142, MI=0.85)  *Haemolaelaps* *glasgowi* (N=93)  *Ornithonyssus sylviarum* (N=49)  **Ticks** (T=53, TI=0.32)  *Haemaphysalis sp.* | Unfortunate hygienic circumstances can increase the zoonotic pathogen transmission through rodent ectoparasites. | Spellings were corrected from *foscocapillus* to *fuscocapillus, Ciurus* to *Sciurus,* *Neohaematopins to Neohaematopinus* and *laeviusculus* to *laeviusulus.* |
| Shirazi et al. [98] | Iran; 2011 | *Sciurus anomalus* (R=1) | **Lice**  *Polyplax* spp. |  |  |
| Soliman-a et al. [99] | Egypt; 1990-92 | *Rattus norvegicus* (N=277)  *Rattus rattus* (N=242)  R=519, Rt=498, Rm=482, Rl=438 | **Fleas** (F=19695, FiR=95.95%, FI=37.95)  *Ctenocephalides felis felis* (N=96)  *Echidnophaga gallinacea* (N=10122)  *Leptopsylla segnis* (N=4260)  *Xenopsylla cheopis* (N=5217)  **Lice** (L=23228, LiR=84.39%, LI=44.76)  *Hoplopleura oenomydis* (N=1368)  *Polyplax spinulosa* (N=21860)  **Mites** (M=18717, MiR=92.87%, MI=36.06)  *Cheyletus eruditus* (N=60)  *Echinolaelaps echidninus* (N=42  *Laelaps nuttalli* (N=7279)  *Ornithonyssus bacoti* (N=8685)  *Radfordia ensifera* (N=2515)  *Trichoecius* sp*.* (N=33)  *TyrophagusTryohagus* sp*.* (N=42)  *Unidentified astigmatic sp.* (N=15)  *Unidentified uropodid* sp*.* (N=32)  *Zygoribatula* spp. (N=14) | Ectoparasite index is subject to seasonal variations and differs according to the geographical location. Mites and fleas get more infestation on *Rattus norvegicus* than on *Rattus rattus*. *Xenopsylla cheopis* is the most frequent flea on rodents. | *Tryohagus is corrected to Tyrophagus* |
| Soliman-b [100] | Egypt; 1990-92 | *Rattus norvegicus* (N=277)  *Rattus rattus* (N=242)  R=519 (f=242, m=277) | **Fleas**  *Echidnophaga gallinacea*  *Leptopsylla segnis*  *Xenopsylla cheopis*  **Lice**  *Polyplax spinulosa*  **Mites**  *Laelaps nuttalli*  *Ornithonyssus bacoti*  *Radfordia ensifera* | The abundance is higher in males than females, however, it is not influenced by the rodent’s age or body size |  |
| Soliman et al. [101] | Egypt; 2009 | *Mus musculus* (N=18)  *Rattus norvegicus* (N=147)  *Rattus rattus alexandrines* (N=39)  *Rattus rattus frugivorous* (N=95)  R=299 (f=135, m=164) | **Fleas** (F=2478, FI=8.4)  *Ctenocephalides canis* (N=20)  *Leptopsylla segnis* (N=234)  *Xenopsylla cheopis* (N=2224) | Adult fleas showed higher infestation frequency than juveniles. | Spelling is corrected from *Lyptopsylla* to *Leptopsylla* |
| Stekol'nikov [102] | Turkey; 1998 | *Apodemus sylvaticus*  *Cricetulus migratorius*  *Meriones libycus*  *Meriones persicus*  *Microtus majori*  *Tatera indica* | **Mites**  *Neotrombicula lazistanica*  *Neotrombicula faghihi*  *Neotrombicula sabzavari*  *Neotrombicula subtilis* |  | Spelling is corrected from *lybicus* to *libycus* |
| Stekolnikov et al. [103] | Saudi Arabia; 2017-18 | *Acomys dimidiatus*  *Meriones rex*  *Myomyscus yemeni* | **Mites**  *Ascoschoengastia browni*  *Ericotrombidium caucasicum*  *Ericotrombidium galliardi*  *Ericotrombidium kazeruni*  *Gahrliepia lawrencei*  *Helenicula lukshumiae*  *Microtrombicula centropi*  *Microtrombicula hoogstraali*  *Microtrombicula hyraci*  *Microtrombicula microscuta*  *Microtrombicula muhaylensis*  *Microtrombicula traubi*  *Pentidionis agamae*  *Schoengastiella wansoni*  *Schoutedenichia asirensis*  *Schoutedenichia saudi*  *Schoutedenichia thracica*  *Schoutedenichia zarudnyi*  *Walchia parvula* |  |  |
| Tajedin et al. [104] | Iran; 2008 | *Rhombomys opimus* (R=50) | **Fleas** (F=73, FI=1.46)  *Xenopsylla nuttalli*  **Mites** (M=24, MI=0.48)  *Ornithonyssus bacoti* | Fleas were the most common ectoparasite on *Rhombomys opimus* rodents. |  |
| Telmadarraiy et al. [105] | Iran; 2004-05 | *Meriones persicus* (N=5)  *Microtus socialis* (N=36)  *Mus musculus* (N=19)  *Nesokia indica* (N=5)  *Rattus rattus* (N=35)  *Tatera indica* (N=39)  R=139 | **Fleas** (F=33, FI=0.24)  *Nosopsyllus medus*  *Pulex irritans*  *Xenopsylla buxtoni*  **Lice** (L=584, LI=4.2)  *Polyplax spinulosa*  **Mites** (M=42, MI=0.3)  *Dermanyssus sanguineus*  *Laelaps nuttalli*  *Ornithonyssus bacoti*  **Ticks** (T=93, TI=0.67)  *Rhipicephalus* spp.  *Hyalomma* spp*.* | The ectoparasites on some rodent hosts tend to prefer particular body sites, and the preferred sites by some ectoparasite species may overlap, minly because they are inaccessible to the host. | Spelling is corrected from *spinolosa* to *spinulosa*, *Dermanysus* to *Dermanyssus*  and *Orithonysus* to *Ornithonyssus* |
| Uslu et al. [106] | Turkey | *Spermophilus citellus*  R=100, Rf=11, Rt=7 | **Fleas** (F=22, FiR=11%, FI=0.22)  *Nosopsyllus fasciatus* (N=21)  *Pulex irritans (*N=1)  **Ticks** (T=8, TiR=7%, TI=0.08)  *Haemaphysalis* spp. (N=5)  *Ixodes* spp*.* (N=3) | Ectoparasites abundance does not vary with the gender or age of rodent hosts. | *Citellus citellus* is changed to homotypic synonym *Spermophilus citellus* |
| Yeruham et al. [107] | Israel; 1983-85 | *Acomys cahirinus* (N=37)  *Mus musculus* (N=17)  R=54, Rt=18 | **Ticks** (T=84, TiR=33.33%, TI=1.54)  *Ixodes eldaricus* | Most of the ticks were larval or nymph stage on the rodents. |  |
| Younis et al. [108] | Egypt | *Acomys cahirinus* (N=39)  *Gerbillus gerbillus asyutensis* (N=17)  *Mus musculus praetextus* (N=13)  *Rattus norvegicus* (N=84)  *Rattus rattus alexandrines* (N=54)  *Rattus rattus frugivorous* (N=34)  R=241 | **Mites** (M=168, MI=0.7)  *Laelaps nuttalli* (N=70)  *Ornithonyssus bacoti* (N=98)  **Ticks** (T=35, TI=0.15)  *Hyalomma* spp*.* (N=24)  *Rhipicephalus* spp*.* (N=11) |  |  |
| Yousefi et al. [109] | Iran; 2011 | *Apodemus sylvaticus* | **Fleas** (F=8)  *Leptopsylla taschenbergi taschenbergi* |  |  |
| Yousefi et al. [110] | Iran; 2010-13 | *Apodemus sylvaticus* (N=53)  *Arvicola amphibius* (N=8)  *Mus musculus* (N=52)  R=113 (f=49, m=64) | **Fleas**  *Leptopsylla taschenbergi*  *Nosopsyllus fasciatus tschenbergi*  **Lice**  *Polyplax spinulosa*  *Polyplax* spp.  **Ticks**  *Boophilus annulatus*  *Haemaphysalis* sp*.*  *Hyalomma* sp*.*  *Ixodes* sp*.*  *Ornithodoros* sp*.*  *Rhipicephalus bursa* | There was no significant correlation between ectoparasites infections and neither the gender of rodents nor the altitude. | *Arvicola amphibius* is the synonym of *Arvicola terrestris* (the correct spelling is *terrestris).* |
| Zarei et al. [111] | Iran; 2017 | *Cricetulus migratorius* (N=18)  *Meriones persicus* (N=151)  *Mus musculus* (N=35)  R=204 | **Fleas** (F=510, FI=2.5)  *Ctenocephalides felis* (N=3)  *Ctenophthalmus rettigi smiti* (N=3)  *Nosopsyllus fasciatus* (N=12)  *Nosopsyllus iranus* (N=4)  *Xenopsylla astia* (N=11)  *Xenopsylla buxtoni* (N=2)  *Xenopsylla cheopis* (N=10)  *Xenopsylla nubica* (N=465)  **Lice** (M=8, MI=0.04)  *Polyplax* spp*.*  **Mites** (L=3, LI=0.01)  *Ornithonyssus* sp*.* | The most common species of flea was *Xenopsylla* sp. |  |
| Zeese et al. [112] | Egypt; 1989 | *Gerbillus gerbillus* (N=9)  *Mus musculus* (N=19)  *Rattus norvegicus* (N=110)  *Rattus rattus* (N=32)  R=170 | **Fleas** (F=871, FI=5.12)  *Ctenocephalides felis* (N=42)  *Echidnophaga gallinacea* (N=560)  *Leptopsylla segnis* (N=83)  *Pulex irritans* (N=9)  *Xenopsylla cheopis* (N=177)  **Lice** (L=666, LI=3.92)  *Polyplax spinulosa*  **Mites** (M=23, MI=0.14)  *Ornithonyssus bacoti* |  |  |
| Zendehfili et al. [113] | Iran; 2012-13 | *Mus musculus* (N=5)  *Rattus norvegicus* (N=92)  *Rattus rattus* (N=8)  R=105 | **Lice** (L=9, LI=0.09)  *Polyplax spinulosa*  **Mites** (M=140, MI=1.33)  *Dermanyssus* spp*.* (N=123)  *Hypoaspis astronomica* (N=15)  *Pachylaelapidae* (N=2)  **Ticks** (T=21, TI=0.2)  *Rhipicephalus* sp. |  | Spelling is corrected from *Dermanyssius* to *Dermanyssus* |
| N: Total number per species, R: total number of rodents, Rf: Total rodents infested with fleas, Rm: Total rodents infested with mites, Rt: Total rodents infested with ticks, Rl: Total rodents infested with lice, f: Total females, m: Total males, F: Total fleas, M: Total mites, T: Total ticks, L: Total lice, FI: Flea Index, MI: Mite Index, TI: Tick Index, LI: Louse Index, FiR: Flea Infestation Rate, MiR: Mite Infestation Rate, TiR: Tick Infestation Rate, LiR: Louse Infestation Rate | | | | | |

# References of Supplementary Table S3

1. Abd El-Halim, A.S.; Allam, K.A.; Metwally, A.M.; El Boraey, A.M. Seasonal variation of infestation rate with lice, tick and mite among rodents in certain Egyptian regions. *Journal of the Egyptian Society of Parasitology* **2009**, *39*, 617-624.

2. Abdel-Rahman, E.H.; Abdelgadir, M.; AlRashidi, M. Ectoparasites burden of House mouse (Mus musculus linnaeus, 1758) from Hai'l of Saudi Arabia. *Saudi Journal of Biological Sciences* **2020**, *27*, 2238-2244.

3. Abo-Elmaged, T.M.; Desoky, A.E.A.S.S. Parasitological survey of rodent in cultivated and reclaimed land at Assiut, Egypt. *Asian Journal of Applied Sciences* **2014**, *7*, 96-101.

4. Abu-Madi, M.A.; Lewis, J.W.; Mikhail, M.; El-Nagger, M.E.; Behnke, J.M. Monospecific helminth and arthropod infections in an urban population of brown rats from Doha, Qatar. *Journal of Helminthology* **2001**, *75*, 313-320.

5. Abu-Madi, M.A.; Behnke, J.M.; Mikhail, M.; Lewis, J.W.; Al-Kaabi, M.L. Parasite populations in the brown rat Rattus norvegicus from Doha, Qatar between years: The effect of host age, sex and density. *Journal of Helminthology* **2005**, *79*, 105-111, doi:10.1079/JOH2005274.

6. Acici, M.; Demirtas, S.; Umur, S.; Gurler, A.T.; Bolukbas, C.S. Infestations of flea species on small, wild mammals in the provinces of Aydin and Manisa in the Aegean Region, Turkey. *Turkish Journal of Veterinary & Animal Sciences* **2017**, *41*, 449-452, doi:10.3906/vet-1610-68.

7. Aktaş, M. Ctenophthalmus harputus, a new Spalax flea from Turkey. *Med Vet Entomol* **1989**, *3*, 23-27, doi:10.1111/j.1365-2915.1989.tb00470.x. RAYYAN-INCLUSION: {"Md Mazharul"=>"Included"}.

8. Al Hindi, A.I.; Abu-Haddaf, E. Gastrointestinal parasites and ectoparasites biodiversity of Rattus rattus trapped from Khan Younis and Jabalia in Gaza strip, Palestine. *Journal of the Egyptian Society of Parasitology* **2013**, *43*, 259-268.

9. Alahmed, A.M.; Al-Dawood, A.S. Rodents and their ectoparasites in Wadi Hanifah, Riyadh City, Saudi Arabia. *Journal of the Egyptian Society of Parasitology* **2001**, *31*, 737-743.

10. Al-Awadi, A.R.; Al-Kazemi, N.; Ezzat, G.; Saah, A.J.; Shepard, C.; Zaghloul, T.; Gherdian, B. Murine typhus in Kuwait in 1978. *Bulletin of the World Health Organization* **1982**, *60*, 283-289.

11. Allam, K.A.; Shalaby, A.A.; Ashour, M.A. Seasonal distribution of fleas infesting rodents in various Egyptian eco-geographical areas and their susceptibility to malathion. *Journal of the Egyptian Society of Parasitology* **2002**, *32*, 405-414.

12. Allymehr, M.; Tavassoli, M.; Manoochehri, M.H.; Ardavan, D. Ectoparasites and gastrointestinal helminths of house mice (mus musculus) from poultry houses in northwest Iran. *Comparative Parasitology* **2012**, *79*, 283-287.

13. Al-Mohammed, H.I. Taxonomical studies of ticks infesting wild rodents from Asir Province in Saudi Arabia. *Journal of the Egyptian Society of Parasitology* **2008**, *38*, 1-8.

14. Alsarraf, M.; Mierzejewska, E.J.; Mohallal, E.M.E.; Behnke, J.M.; Bajer, A. Genetic and phylogenetic analysis of the ticks from the Sinai Massif, Egypt, and their possible role in the transmission of Babesia behnkei. *Exp Appl Acarol* **2017**, *72*, 415-427, doi:10.1007/s10493-017-0164-4. Epub 2017 Aug 28. RAYYAN-INCLUSION: {"Md Mazharul"=>"Included"}.

15. Antoniou, M.; Psaroulaki, A.; Toumazos, P.; Mazeris, A.; Ioannou, I.; Papaprodromou, M.; Georgiou, K.; Hristofi, N.; Patsias, A.; Loucaides, F., et al. Rats as indicators of the presence and dispersal of pathogens in cyprus: Ectoparasites, parasitic helminths, enteric bacteria, and encephalomyocarditis virus. *Vector-Borne and Zoonotic Diseases* **2010**, *10*, 867-873.

16. Arafa, M.S.; Mahdi, A.H.; Khalil, M.S. Seasonal observations on the Cairo spiny mouse, Acomys cahirinus (E. Geoffroy, St. Hilaire, 1803) and its fleas in Egypt. *The Journal of the Egyptian Public Health Association* **1973**, *48*, 60-71.

17. Asiry, K.A.; Fetoh, B.E.A. Occurrence of ectoparasitic arthropods associated with rodents in Hail region northern Saudi Arabia. *Environmental Science and Pollution Research* **2014**, *21*, 10120-10128, doi:10.1007/s11356-014-3016-3.

18. Bacot, A.; Petrie, G.F.; Todd, R.E. The fleas found on rats and other rodents, living in association with man, and trapped in the towns, villages and nile boats of upper Egypt. *Journal of Hygiene* **1914**, *14*, 498-508.

19. Bahgat, I.M. Monthly abundance of rodent and their ectoparasites in newly settled areas, east of lakes, Ismailia Governorate, Egypt. *J Egypt Soc Parasitol* **2013**, *43*, 387-398, doi:10.12816/0006394. RAYYAN-INCLUSION: {"Md Mazharul"=>"Included"}.

20. Bajer, A.; Harris, P.D.; Behnke, J.M.; Bednarska, M.; Barnard, C.J.; Sherif, N.; Clifford, S.; Gilbert, F.S.; Siński, E.; Zalat, S. Local variation of haemoparasites and arthropod vectors, and intestinal protozoans in spiny mice (Acomys dimidiatus) from four montane wadis in the St Katherine Protectorate, Sinai, Egypt. *Journal of Zoology* **2006**, *270*, 9-24.

21. Bakr, M.E.; Morsy, T.A.; Nassef, N.E.; el Meligi, M.A. Mites infesting commensal rodents in Shebin El Kom, Menoufia G., Egypt. *J Egypt Soc Parasitol* **1995**, *25*, 853-859.

22. Bakr, M.E.; Morsy, T.A.; Nassef, N.E.; El Meligi, M.A. Flea ectoparasites of commensal rodents in Shebin El Kom, Menoufia Governorate, Egypt. *Journal of the Egyptian Society of Parasitology* **1996**, *26*, 39-52.

23. Bochkov, A.; Malikov, V.; Arbobi, M. Trichoecius calomysci sp. n. (Acari: Myocoptidae), a new mite species from Iran. *Folia Parasitologica* **1999**, *46*, 316-318.

24. Bochkov, A.; Arbobi, M.; Malikov, V. Notes on mites of the family Myobiidae (Acari: Prostigmata) parasitising rodents (Mammalia: Rodentia) in Iran. *Folia Parasitol (Praha)* **2000**, *47*, 73-77, doi:10.14411/fp.2000.015. RAYYAN-INCLUSION: {"Md Mazharul"=>"Included"}.

25. Chegeni, A.H.; Mostafavi, E.; Mohammadi, A.; Mahmoudi, A.; Kayedi, M.H. The parasitism of Persian jird by immature stages of Hyalomma asiaticum (Acari: Ixodidae) and its identification using molecular approaches in Iran. *Persian Journal of Acarology* **2018**, *7*, 381-392.

26. Christou, C.; Psaroulaki, A.; Antoniou, M.; Toumazos, P.; Ioannou, I.; Mazeris, A.; Chochlakis, D.; Tselentis, Y. Rickettsia typhi and Rickettsia felis in Xenopsylla cheopis and Leptopsylla segnis parasitizing rats in Cyprus. *Am J Trop Med Hyg* **2010**, *83*, 1301-1304, doi:10.4269/ajtmh.2010.10-0118. RAYYAN-INCLUSION: {"Md Mazharul"=>"Included"}.

27. Cicek, H.; Stanyukovich, M.; Yağci, S.; Aktaş, M.; Karaer, Z. Gamasine mite (Parasitiformes: Mesostigmata) infestations of small mammals (Mammalia: Rodentia, Insectivora) in Turkey. *Turkiye Parazitol Derg* **2008**, *32*, 65-70.

28. Dahesh, S.M.; Mikhail, M.W. SURVEILLANCE OF TRYPANOSOMA SPP OF RODENTS AND STUDIES IN THEIR TRANSMISSION PROBABILITY BY FLEAS IN SOME RURAL EGYPTIAN AREAS. *Journal of the Egyptian Society of Parasitology* **2016**, *46*, 157-166.

29. Darvishi, M.M.; Youssefi, M.R.; Changizi, E.; Lima, R.R.; Rahimi, M.T. A new flea from Iran. *Asian Pacific Journal of Tropical Disease* **2014**, *4*, 85-87, doi:<https://doi.org/10.1016/S2222-1808(14)60321-2>.

30. el Bahrawy, A.A.; al Dakhil, M.A. Studies on the ectoparasites (fleas and lice) on rodents in Riyadh and its surroundings, Saudi Arabia. *Journal of the Egyptian Society of Parasitology* **1993**, *23*, 723-735.

31. El Kady, G.A.; Shoukry, A.; Ragheb, D.A.; El Said, A.M.; Habib, K.S.; Morsy, T.A. Mites (acari) infesting commensal rats in Suez Canal zone, Egypt. *J Egypt Soc Parasitol* **1995**, *25*, 417-425.

32. El Kady, G.A.; El Shazly, A.M.; Mikhail, M.W.; Bahgat, I.M. Ectoparasites of commensal rodents in Talkha Center, Dakahlia Governorate, Egypt. *Journal of the Egyptian Society of Parasitology* **2007**, *37*, 825-833.

33. el-Bahrawy, A.A.; al-Dakhil, M.A. Studies on the interrelation between rodents and their ectoparasitic acarines in Riyadh region, Saudi Arabia. *Journal of the Egyptian Society of Parasitology* **1993**, *23*, 675-685.

34. El-Kady, G.A.; Makled, K.M.; Morsy, T.A.; Morsy, Z.S. Rodents, their seasonal activity, ecto- and blood-parasites in Saint Catherine area, South Sinai Governorate, Egypt. *J Egypt Soc Parasitol* **1998**, *28*, 815-826.

35. El-Kammah, K.M.; Oyoun, L.M.; El Kady, G.A. Laelaps sinai sp. nov. (Laelapinae, Laelapidae), a parasite of Gerbillus pyramium in El Arish, North Sinai, Egypt. *J Egypt Soc Parasitol* **1994**, *24*, 167-171.

36. Eslami, A.; Yousefi, A.; Dowling, A.P.G. Prevalence of ectoparasites in black rat (Rattus rattus) from Mangrove forests of Qeshm Island, Iran. *Comparative Clinical Pathology* **2018**, *27*, 1583-1586, doi:10.1007/s00580-018-2777-3.

37. Farhang-Azad, A.; Neronov, V. The flea fauna of the great gerbil (Rhombomys opimus Licht.) in Iran. *Folia Parasitol (Praha)* **1973**, *20*, 343-351.

38. Gaaboub, I.A.; Widaatalla, A.E.E.; Kelada, N.L. Survey of Rats and Mice and Their Ectoparasites in Relation to Cultivated Areas in the Vicinity of Alexandria Governorate, Egypt. *The Journal of Agricultural Science* **1981**, *97*, 551-555.

39. Gaaboub, I.A.; Donia, A.H.; Kelada, N.L.; Abdelkarim, M.E.H. Ectoparasites of some rodents from the edge of the western desert near Alexandria, Egypt. *Insect Science and Its Application* **1982**, *3*, 145-150.

40. Garrett, D.A.; Allred, D.M. Mesostigmatid mites from Turkey, with keys to genera and species. *J Med Entomol* **1971**, *8*, 292-298, doi:10.1093/jmedent/8.3.292. RAYYAN-INCLUSION: {"Md Mazharul"=>"Included"}.

41. Gholipoury, M.; Rezai, H.R.; Namroodi, S.; Arab Khazaeli, F. Zoonotic and non-zoonotic parasites of wild rodents in Turkman Sahra, northeastern Iran. *Iranian Journal of Parasitology* **2016**, *11*, 350-357.

42. Hamidi, K.; Nassirkhani, M. Annotated checklist of fleas (Insecta: Siphonaptera) and lice (Insecta: Anoplura) associated with rodents in Iran, with new reports of fleas and lice. *J Vector Borne Dis* **2019**, *56*, 134-145, doi:10.4103/0972-9062.263715. RAYYAN-INCLUSION: {"Md Mazharul"=>"Included"}.

43. Hanafi-Bojd, A.A.; Shahi, M.; Baghaii, M.; Shayeghi, M.; Razmand, N.; Pakari, A. A study on rodent ectoparasites in Bandar Abbas: The main economic southern seaport of Iran. *Iranian Journal of Environmental Health Science and Engineering* **2007**, *4*, 173-176.

44. Harrison, A.; Robb, G.N.; Alagaili, A.N.; Hastriter, M.W.; Apanaskevich, D.A.; Ueckermann, E.A.; Bennett, N.C. Ectoparasite fauna of rodents collected from two wildlife research centres in Saudi Arabia with discussion on the implications for disease transmission. *Acta Trop* **2015**, *147*, 1-5, doi:10.1016/j.actatropica.2015.03.022. Epub 2015 Mar 27. RAYYAN-INCLUSION: {"Md Mazharul"=>"Included"}.

45. Hawlena, H.; Abramsky, Z.; Krasnov, B.R. Ectoparasites and age-dependent survival in a desert rodent. *Oecologia* **2006**, *148*, 30-39.

46. Hoogstraal, H.; Traub, R. The fleas (Siphonaptera) of Egypt. Host-parasite relationships of rodents of the families Spalacidae, Muridae, Gliridae, Dipodidae, and Hystricidae. *The Journal of the Egyptian Public Health Association* **1965**, *40*, 343-379.

47. Hoogstraal, H.; Kaiser, M.N.; Ormsbee, R.A.; Osborn, D.J.; Hemly, I.; Gaber, S. Hyalomma (Hyalommina) rhipicephaloides Neumann (Ixodoidea: Ixodidae): its identity, hosts, and ecology, and Rickettsia conori, R. prowazeki, and Coxiella burneti infections in rodent hosts in Egypt. *Journal of medical entomology* **1967**, *4*, 391-400.

48. Imam, Z.I.; Salah, A.M. Preliminary notes on typhus amon rodents in U.A.R. *The Journal of the Egyptian Public Health Association* **1966**, *41*, 133-143.

49. Karaer, Z.; Kurtdede, A.; Ural, K.; Sari, B.; Cingi, C.C.; Karakurum, M.C.; Haydardedeoglu, A.E. Demodicosis in a Golden (Syrian) hamster (Mesocricetus auratus). *Ankara Universitesi Veteriner Fakultesi Dergisi* **2009**, *56*, 227-229.

50. Keskin, A.; Beaucournu, J.C. Descriptions of Two New Species and a New Subspecies of the Genus Ctenophthalmus (Insecta: Siphonaptera: Ctenophthalmidae) from Turkey. *J Med Entomol* **2019**, *56*, 1275-1282, doi:10.1093/jme/tjz096. RAYYAN-INCLUSION: {"Md Mazharul"=>"Included"}.

51. Keskin, A.; Selçuk, A.Y.; Kefelioğlu, H. Ticks (Acari: Ixodidae) infesting some small mammals from Northern Turkey with new tick–host associations and locality records. *Experimental and Applied Acarology* **2017**, *73*, 521-526.

52. Keskin, A.; Selçuk, A.; Kefelioğlu, H. Ticks (Acari: Ixodidae) infesting some wild animals and humans in Turkey: notes on a small collection. **2019**, *1*, xx-xx.

53. Keskin, A.; Selçuk, A.Y.; Kefelioğlu, H.; Beaucournu, J.C. Fleas (Insecta: Siphonaptera) collected from some small mammals (Mammalia: Rodentia, Eulipotyphla) in Turkey, with new records and new host associations. *Acta Trop* **2020**, *208*, 105522, doi:10.1016/j.actatropica.2020.105522. RAYYAN-INCLUSION: {"Md Mazharul"=>"Included"}.

54. Khajeh, A.; Razmi, G.; Darvish, J. A study of ectoparasites in wild rodents of the Jaz Murian area in the southeast of Iran. *Asian Pacific Journal of Tropical Disease* **2017**, *7*, 418-421.

55. Kia, E.; Moghddas-Sani, H.; Hassanpoor, H.; Vatandoost, H.; Zahabiun, F.; Akhavan, A.; Hanafi-Bojd, A.; Telmadarraiy, Z. Ectoparasites of rodents captured in bandar abbas, southern iran. *Iran J Arthropod Borne Dis* **2009**, *3*, 44-49.

56. Kim, K.C.; Emerson, K.C. Sucking lice (Anoplura) from Iranian mammals. *Journal of medical entomology* **1971**, *8*, 7-16.

57. Krasnov, B.R.; Shenbrot, G.I.; Khokhlova, I.S.; Degen, A.A.; Rogovin, K.A. On the biology of Sundevall's jird (Meriones crassus Sundevall, 1842) (Rodentia : Gerbillidae) in the Negev Highlands, Israel. In *Mammalia*, 1996; Vol. 60, p 375.

58. Krasnov, B.R.; Shenbrot, G.I.; Medvedev, S.G.; Vatschenok, V.S.; Khokhlova, I.S. Host-habitat relations as an important determinant of spatial distribution of flea assemblages (Siphonaptera) on rodents in the Negev Desert. *Parasitology* **1997**, *114*, 159-173.

59. Krasnov, B.; Shenbrot, G.; Khokhlova, I.; Medvedev, S.; Vatschenok, V. Habitat dependence of a parasite-host relationship: flea (Siphonaptera) assemblages in two gerbil species of the Negev Desert. *J Med Entomol* **1998**, *35*, 303-313, doi:10.1093/jmedent/35.3.303. RAYYAN-INCLUSION: {"Md Mazharul"=>"Included"}.

60. Krasnov, B.R.; Hastriter, M.W.; Medvedev, S.G.; Shenbrot, G.I.; Khokhlova, I.S.; Vatschenok, V.S. Additional records of fleas (siphonaptera) on wild rodents in the southern part of Israel. *Israel Journal of Zoology* **1999**, *45*, 333-340.

61. Krasnov, B.R.; Burdelova, N.V.; Shenbrot, G.I.; Khokhlova, I.S. Annual cycles of four flea species in the central Negev desert. *Med Vet Entomol* **2002**, *16*, 266-276, doi:10.1046/j.1365-2915.2002.00374.x. RAYYAN-INCLUSION: {"Md Mazharul"=>"Included"}.

62. Krasnov, B.R.; Khokhlova, I.S.; Shenbrot, G.I. Density-dependent host selection in ectoparasites: An application of isodar theory to fleas parasitizing rodents. *Oecologia* **2003**, *134*, 365-372.

63. Krasnov, B.R.; Morand, S.; Khokhlova, I.S.; Shenbrot, G.I.; Hawlena, H. Abundance and distribution of fleas on desert rodents: Linking Taylor's power law to ecological specialization and epidemiology. *Parasitology* **2005**, *131*, 825-837.

64. Krasnov, B.R.; Shenbrot, G.I.; Khokhlova, I.S.; Hawlena, H.; Degen, A.A. Sex ratio in flea infrapopulations: number of fleas, host gender and host age do not have an effect. *Parasitology* **2008**, *135*, 1133-1141, doi:10.1017/s0031182008004551.

65. Lehmann, T. Ectoparasite impacts on Gerbillus andersoni allenbyi under natural conditions. *Parasitology* **1992**, *104 ( Pt 3)*, 479-488, doi:10.1017/s0031182000063745.

66. Lehmann, T. Reproductive activity of Synosternus cleopatrae (Siphonaptera: Pulicidae) in relation to host factors. *J Med Entomol* **1992**, *29*, 946-952, doi:10.1093/jmedent/29.6.946. RAYYAN-INCLUSION: {"Md Mazharul"=>"Included"}.

67. Lewis, R.E. A preliminary list of the fleas of Lebanon. *Proceedings of the Royal Entomological Society of London. Series A, General Entomology* **1962**, *37*, 49-60, doi:<https://doi.org/10.1111/j.1365-3032.1962.tb00287.x>.

68. Lewis, R.E. The fleas (Siphonaptera) of Egypt. New records. *The Journal of parasitology* **1966**, *52*, 1167-1171.

69. Loftis, A.D.; Reeves, W.K.; Szumlas, D.E.; Abbassy, M.M.; Helmy, I.M.; Moriarity, J.R.; Dasch, G.A. Surveillance of Egyptian fleas for agents of public health significance: Anaplasma, bartonella, coxiella, ehrlichia, rickettsia, and Yersinia pestis. *American Journal of Tropical Medicine and Hygiene* **2006**, *75*, 41-48.

70. Mahdi, A.H.; Arafa, M.S. Seasonal observations on the house mouse, Mus musculus (Cretzeschmar, 1826), and its fleas in Alexandria, U.A.R. *The Journal of the Egyptian Public Health Association* **1971**, *46*, 106-113.

71. Mikhail, M.W.; Soliman, M.I.; Abd el, H.A. Infestation rate of tick, mite and lice among rodent species in Menoufia governorate, Egypt. *J Egypt Soc Parasitol* **2010**, *40*, 425-438.

72. Mohammadi, A.; Sedaghat, M.M.; Abai, M.R.; Darvish, J.; Mobedi, I.; Mahmoudi, A.; Mostafavi, E. Wild Rodents and Their Ectoparasites in an Enzootic Plague Focus, Western Iran. *Vector Borne Zoonotic Dis* **2020**, *20*, 334-347, doi:10.1089/vbz.2019.2524. Epub 2020 Feb 20. RAYYAN-INCLUSION: {"Md Mazharul"=>"Included"}.

73. Moravvej, G.; Hamidi, K.; Nourani, L.; Bannazade, H. Occurrence of ectoparasitic arthropods (Siphonaptera, Acarina, and Anoplura) on rodents of Khorasan Razavi Province, northeast of Iran. *Asian Pacific Journal of Tropical Disease* **2015**, *5*, 716-720.

74. Morick, D.; Baneth, G.; Avidor, B.; Kosoy, M.Y.; Mumcuoglu, K.Y.; Mintz, D.; Eyal, O.; Goethe, R.; Mietze, A.; Shpigel, N., et al. Detection of Bartonella spp. in wild rodents in Israel using HRM real-time PCR. *Veterinary microbiology* **2009**, *139*, 293-297, doi:10.1016/j.vetmic.2009.06.019.

75. Morick, D.; Krasnov, B.R.; Khokhlova, I.S.; Shenbrot, G.I.; Kosoy, M.Y.; Harrus, S. Bartonella Genotypes in Fleas (Insecta: Siphonaptera) Collected from Rodents in the Negev Desert, Israel. *Applied and Environmental Microbiology* **2010**, *76*, 6864-6869, doi:10.1128/aem.00879-10.

76. Morsy, T.A.; Michael, S.A.; Bassili, W.R.; Saleh, M.S. Studies on rodents and their zoonotic parasites, particularly leishmania, in Ismailiya Governorate, A.R. Egypt. *Journal of the Egyptian Society of Parasitology* **1982**, *12*, 565-585.

77. Morsy, T.A.; Fayad, M.E.; Abou Shady, M.K.; Yousef, N.S. Ectoparasites of rodents in Suez governorate with special reference to fleas. *Journal of the Egyptian Society of Parasitology* **1986**, *16*, 457-468.

78. Morsy, T.A.; el-Ela, R.G.; el Gozamy, B.M. The commensal rodents and their flea fauna in Alexandria City, Egypt. *Journal of the Egyptian Society of Parasitology* **1988**, *18*, 11-28.

79. Morsy, T.A.; El Bahrawy, A.F.; El Dakhil, M.A. Ecto- and blood parasites affecting Meriones rex trapped in Najran, Saudi Arabia. *J Egypt Soc Parasitol* **2001**, *31*, 399-405.

80. Mostafavi, E.; Shahraki, A.H.; Japoni-Nejad, A.; Esmaeili, S.; Darvish, J.; Sedaghat, M.M.; Mohammadi, A.; Mohammadi, Z.; Mahmoudi, A.; Pourhossein, B., et al. A Field Study of Plague and Tularemia in Rodents, Western Iran. *Vector-Borne and Zoonotic Diseases* **2017**, *17*, 247-253, doi:10.1089/vbz.2016.2053.

81. Mumcuoglu, K.Y.; Frish, K.; Sarov, B.; Manor, E.; Gross, E.; Gat, Z.; Galun, R. Ecological studies on the brown dog tick Rhipicephalus sanguineus (Acari: Ixodidae) in southern Israel and its relationship to spotted fever group rickettsiae. *Journal of medical entomology* **1993**, *30*, 114-121.

82. Mumcuoglu, K.Y.; Ioffe-Uspensky, I.; Alkrinawi, S.; Sarov, B.; Manor, E.; Galun, R. Prevalence of vectors of the spotted fever group Rickettsiae and murine typhus in a Bedouin town in Israel. *Journal of Medical Entomology* **2001**, *38*, 458-461, doi:10.1603/0022-2585-38.3.458.

83. Nasereddin, A.; Risheq, A.; Harrus, S.; Azmi, K.; Ereqat, S.; Baneth, G.; Salant, H.; Mumcuoglu, K.Y.; Abdeen, Z. Bartonella species in fleas from Palestinian territories: Prevalence and genetic diversity. *Journal of Vector Ecology* **2014**, *39*, 261-270.

84. Nateghpour, M.; Akhavan, A.A.; Hanafi-Bojd, A.A.; Telmadarraiy, Z.; Ayazian Mavi, S.; Hosseini-Vasoukolaei, N.; Motevalli-Haghi, A.; Akbarzadeh, K. Wild rodents and their ectoparasites in Baluchistan area, southeast of Iran. *Tropical Biomedicine* **2013**, *30*, 72-77.

85. Oyoun, L.M.; el Kammah, K.M.; el Kady, G.A. The fur mite Listrophorus arishi: sp. nov. (Listrophorinae, Listrophoridae) of jerboes in North Sinai, Egypt. *J Egypt Soc Parasitol* **1994**, *24*, 173-176.

86. Pourhossein, B.; Esmaeili, S.; Gyuranecz, M.; Mostafavi, E. Tularemia and plague survey in rodents in an earthquake zone in southeastern Iran. *Epidemiology and Health* **2015**, *37*.

87. Psaroulaki, A.; Antoniou, M.; Papaeustathiou, A.; Toumazos, P.; Loukaides, F.; Tselentis, Y. First detection of Rickettsia felis in Ctenocephalides felis fleas parasitizing rats in Cyprus. *Am J Trop Med Hyg* **2006**, *74*, 120-122.

88. Psaroulaki, A.; Antoniou, M.; Toumazos, P.; Mazeris, A.; Ioannou, I.; Chochlakis, D.; Christophi, N.; Loukaides, P.; Patsias, A.; Moschandrea, I., et al. Rats as indicators of the presence and dispersal of six zoonotic microbial agents in Cyprus, an island ecosystem: a seroepidemiological study. *Transactions of the Royal Society of Tropical Medicine and Hygiene* **2010**, *104*, 733-739, doi:10.1016/j.trstmh.2010.08.005.

89. Psaroulaki, A.; Chochlakis, D.; Ioannou, I.; Angelakis, E.; Tselentis, Y. Presence of Coxiella burnetii in Fleas in Cyprus. *Vector-Borne and Zoonotic Diseases* **2014**, *14*, 685-687, doi:10.1089/vbz.2013.1399.

90. Rahdar, M.; Vazirianzadeh, B.; Rointan, E.S.; Amraei, K. Identification of collected ectoparasites of rodents in the west of Khuzestan Province (Ahvaz and Hovizeh), southwest of Iran. *Asian Pacific Journal of Tropical Disease* **2015**, *5*, 627-631.

91. Reeves, W.K.; Szumlas, D.E.; Moriarity, J.R.; Loftis, A.D.; Abbassy, M.M.; Helmy, I.M.; Dasch, G.A. Louse-borne bacterial pathogens in lice (Phthiraptera) of rodents and cattle from Egypt. *Journal of Parasitology* **2006**, *92*, 312-318, doi:10.1645/0022-3395(2006)92[312:BR]2.0.CO;2.

92. Reeves, W.K.; Loftis, A.D.; Szumlas, D.E.; Abbassy, M.M.; Helmy, I.M.; Hanafi, H.A.; Dasch, G.A. Rickettsial pathogens in the tropical rat mite Ornithonyssus bacoti (Acari: Macronyssidae) from Egyptian rats (Rattus spp.). *Experimental and Applied Acarology* **2007**, *41*, 101-107, doi:10.1007/s10493-006-9040-3.

93. Rifaat, M.A.; Morsy, T.A.; Abdel Mawla, M.M. Seasonal activity of Rattus norvegicus and flea index in Port Said Governorate, Egypt. *J Egypt Soc Parasitol* **1981**, *11*, 525-532.

94. Rzotkiewicz, S.; Gutiérrez, R.; Krasnov, B.R.; Morick, D.; Khokhlova, I.S.; Nachum-Biala, Y.; Baneth, G.; Harrus, S. Novel evidence suggests that a 'Rickettsia felis-like' organism is an endosymbiont of the desert flea, Xenopsylla ramesis. *Mol Ecol* **2015**, *24*, 1364-1373, doi:10.1111/mec.13106. Epub 2015 Mar 6. RAYYAN-INCLUSION: {"Md Mazharul"=>"Included"}.

95. Sanborn, C.C.; Hoogstraal, H. *Some mammals of Yemen and their ectoparasites*; Chicago Natural History Museum: Chicago, 1953.

96. Shamsi, M.; Stekolnikov, A.A.; Saboori, A.; Hakimitabar, M.; Golpayegani, A.Z. Contributions to the fauna of chigger mites (Acariformes: Trombiculidae) of Iran. *Zootaxa* **2020**, *4834*, 301-355.

97. Shayan, A.; Rafinejad, J. Arthropod parasites of rodents in Khorram Abbad district, Lorestan Provincen of Iran. *Iranian Journal of Public Health* **2006**, *35*, 70-76.

98. Shirazi, S.; Bahadori, F.; Mostafaei, T.S.; Ronaghi, H. First report of Polyplax sp. in a Persian squirrel (Scuirus anomalus) in Tabriz, Northwest of Iran. *Türkiye parazitolojii dergisi / Türkiye Parazitoloji Derneǧi = Acta parasitologica Turcica / Turkish Society for Parasitology* **2013**, *37*, 299-301, doi:10.5152/tpd.2013.3085.

99. Soliman, S.; Main, A.J.; Marzouk, A.S.; Montasser, A.A. Seasonal studies on commensal rats and their ectoparasites in a rural area of Egypt: The relationship of ectoparasites to the species, locality, and relative abundance of the host. *Journal of Parasitology* **2001**, *87*, 545-553.

100. Soliman, S.; Marzouk, A.S.; Main, A.J.; Montasser, A.A. Effect of sex, size, and age of commensal rat hosts on the infestation parameters of their ectoparasites in a rural area of Egypt. *J Parasitol* **2001**, *87*, 1308-1316, doi:10.1645/0022-3395(2001)087[1308:EOSSAA]2.0.CO;2. RAYYAN-INCLUSION: {"Md Mazharul"=>"Included"}.

101. Soliman, M.I.; Abd El-Halim, A.S.; Mikhail, M.W. Rodent borne diseases and their fleas in Menoufia Governorate, Egypt. *Journal of the Egyptian Society of Parasitology* **2010**, *40*, 107-117.

102. Stekol'nikov, A.A. A new subgenus and species of the chigger mite genus Neotrombicula (Acari: Trombiculidae). *Acarologia* **1999**, *40*, 407-412.

103. Stekolnikov, A.A.; Al-Ghamdi, S.Q.; Alagaili, A.N.; Makepeace, B.L. First data on chigger mites (Acariformes: Trombiculidae) of Saudi Arabia, with a description of four new species. *Systematic and Applied Acarology* **2019**, *24*, 1937-1963.

104. Tajedin, L.; Rassi, Y.; Oshaghi, M.; Telmadarraiy, Z.; Akhavan, A.; Abai, M.; Arandian, M. Study on Ectoparasites of Rhombomys opimus, the Main Reservoir of Zoonotic Cutaneous Leishmaniasis in Endemic Foci in Iran. *Iran J Arthropod Borne Dis* **2009**, *3*, 41-45.

105. Telmadarraiy, Z.; Vatandoost, H.; Mohammadi, S.; Akhavan, A.A.; Abai, M.R.; Rafinejad, J.; Kia, E.B.; Naini, F.F.; Jedari, M.; Aboulhasani, M. Determination of Rodent Ectoparasite Fauna in Sarpole-Zahab District, Kermanshah Province, Iran, 2004-2005. *Iranian Journal of Arthropod-Borne Disease* **2007**, *1*, 5.

106. Uslu, U.; Dik, B.; Gökçen, A. Ectoparasites of the ground squirrel (Citellus citellus (L.)) in Turkey. *Turkiye Parazitol Derg* **2008**, *32*, 142-145.

107. Yeruham, I.; Hadani, A.; Galker, F.; Rosen, S. The occurrence of Ixodes-Eldaricus (Dzhaparidze, 1950) (Acarina, Ixodidae) in Israel. *Acarologia* **1995**, *36*, 191-193.

108. Younis, T.A.; Fayad, M.E.; el Hariry, M.A.; Morsy, T.A. Interaction between acari ectoparasites and rodents in Suez Governorate, Egypt. *Journal of the Egyptian Society of Parasitology* **1995**, *25*, 377-394.

109. Yousefi, A.; Nosrati, M.R.C.; Karimi, A.; Naisi, S. Leptopsylla taschenbergi taschenbergi (Siphonaptera: Leptopsyllidae), new flea from Iran. *Asian Pacific Journal of Tropical Disease* **2015**, *5*, 606-607.

110. Yousefi, A.; Rahbari, S.; Eslami, A. Ectoparasites associated with small mammals (orders Insectivora, Eulipotyphla, and Rodentia) in Razan plain, western region of Iran. *Comparative Clinical Pathology* **2018**, *27*, 667-671.

111. Zarei, Z.; Mohebali, M.; Heidari, Z.; Kia, E.B.; Azarm, A.; Bakhshi, H.; Davoodi, J.; Hassanpour, H.; Roohnavaz, M.; Khodabakhsh, M., et al. Wild Rodent Ectoparasites Collected from Northwestern Iran. *Journal of Arthropod-Borne Diseases* **2017**, *11*, 36-41.

112. Zeese, W.; Khalaf, S.A.; Abou el-Ela, R.G.; Morsy, T.A. Rodents and their ectoparasites in Sharkia Governorate, Egypt. *Journal of the Egyptian Society of Parasitology* **1990**, *20*, 827-835.

113. Zendehfili, H.; Zahirnia, A.H.; Maghsood, A.H.; Khanjani, M.; Fallah, M. Ectoparasites of rodents captured in Hamedan, Western Iran. *Journal of Arthropod-Borne Diseases* **2015**, *9*, 267-273.
