# Supplementary material for: Rodent Ectoparasites in the Middle East: A Systematic Review and Meta-Analysis
Source: Pathogens. 2021 Jan 31;10(2):139. doi: 10.3390/pathogens10020139 (PMC7911898; doi:10.3390/pathogens10020139)
Supplement: Supplementary file 1 [file pathogens-10-00139-s001.zip › Supplementary documents/Supplimentary Table S4.docx]

Rodent Ectoparasites in the Middle East: A Systematic Review and Meta-Analysis

**Supplementary Table S4:** **Rodents, fleas, lice, mites, and ticks prevailing on rodents in the Middle East**

## Supplementary Table S4a: Rodents in the Middle East

| 1. **Family:** Calomyscidae  *Calomyscus bailwardi* (Iran), *Calomyscus elburzensis* (Iran), *Calomyscus hotsoni* (Iran), *Calomyscus* sp. (Iran) |
| --- |
| 2. **Family:** Cricetidae  *Arvicola amphibius* (Iran), *Chionomys nivalis* (Iran, Turkey), *Chionomys* sp. (Iran), *Clethrionomys* sp. (Iran), *Cricetulus migratorius* (Iran, Lebanon, Turkey), *Ellobius fuscocapillus* (Iran), *Mesocricetus auratus* (Turkey), *Mesocricetus brandti* (Turkey), *Microtus arvalis* (Turkey), *Microtus guentheri* (Lebanon, Turkey), *Microtus levis* (Turkey), *Microtus majori* (Turkey), *Microtus mystacinus* (Iran), *Microtus paradoxus* (Iran), *Microtus qazvinensis* (Iran), *Microtus socialis* (Iran, Israel), *Microtus* sp. (Iran, Turkey), *Microtus subterraneus* (Turkey), *Microtus transcaspicus* (Iran), *Myodes glareolus* (Turkey), *Prometheomys schaposchnikowi* (Turkey) |
| 3. **Family:** Dipodidae  *Jaculus blanfordi* (Iran), *Jaculus jaculus* (Egypt, Israel, Saudi Arabia), *Jaculus orientalis* (Egypt), *Scarturus elater* (Iran), *Scarturus migratorius* (Iran), *Scarturus* sp. (Iran), *Scarturus tetradactyla* (Egypt), *Sciurus anomalus* (Iran, Lebanon) |
| 4. **Family:** Gliridae  *Dryomys nitedula* (Iran), *Eliomys melanurus* (Egypt, Israel), *Eliomys quercinus* (Egypt) |
| 5. **Family:** Muridae  *Acomys cahirinus* (Egypt, Iran, Israel), *Acomys dimidiatus* (Egypt, Iran, Saudi Arabia), *Acomys russatus* (Egypt, Israel), *Apodemus agrarius* (Turkey), *Apodemus flavicollis* (Iran, Turkey), *Apodemus mystacinus* (Iran, Lebanon, Turkey), *Apodemus ponticus* (Iran), *Apodemus* spp. (Iran, Turkey), *Apodemus sylvaticus* (Iran, Israel, Lebanon, Turkey), *Apodemus uralensis* (Turkey), *Apodemus witherbyi* (Iran, Turkey), *Arvicanthis niloticus* (Egypt), *Dipodillus dasyurus* (Egypt, Israel), *Gerbillus andersoni* (Israel), *Gerbillus cheesmani* (Iran, Saudi Arabia), *Gerbillus dasyurus* (Israel), *Gerbillus gerbillus* (Egypt, Israel), *Gerbillus henleyi* (Israel), *Gerbillus nanus* (Iran, Israel, Saudi Arabia), *Gerbillus pyramidum* (Egypt, Israel), *Gerbillus* spp. (Saudi Arabia), *Golunda ellioti* (Iran), *Meriones crassus* (Egypt, Iran, Israel), *Meriones hurrianae* (Iran), *Meriones libycus* (Iran, Saudi Arabia, Turkey), *Meriones meridianus* (Iran), *Meriones nivalis* (Lebanon), *Meriones persicus* (Iran, Turkey), *Meriones rex* (Egypt, Saudi Arabia), *Meriones sacramenti* (Israel), *Meriones* spp. (Iran, Saudi Arabia, Turkey), *Meriones tamariscinus* (Iran), *Meriones tristrami* (Iran, Israel, Lebanon, Turkey), *Meriones vinogradovi* (Iran), *Mus macedonicus* (Iran, Turkey), *Mus musculus* (Cyprus, Egypt, Iran, Israel, Saudi Arabia, Turkey), *Mus spicilegus* (Iran), *Psammomys obesus* (Israel), *Nesokia indica* (Iran), *Rattus norvegicus* (Cyprus, Egypt, Iran, Israel, Kuwait, Lebanon, Qatar, Saudi Arabia), *Rattus pyctoris* (Iran), *Rattus rattus* (Cyprus, Egypt, Iran, Israel, Lebanon, Palestine, Saudi Arabia, Turkey), *Rhombomys opimus* (Iran), *Tatera indica* (Iran, Turkey), *Myomyscus Yemeni* (Saudi Arabia), *Sekeetamys calurus* (Egypt, Israel) |
| 6. **Family:** Sciuridae  *Sciurus anomalus* (Iran, Lebanon) |
| 7. **Family:** Spalacidae  *Nannospalax xanthodon* (Turkey), *Spalax leucodon* (Turkey). *Spermophilus citellus* (Turkey), *Spermophilus fulvus* (Iran) |

## Supplementary Table S4b: Rodent fleas reported in the Middle East

| 1. **Family:** Ceratophyllidae  *Callopsylla caspia* (Lebanon), *Ceratophyllus hirundinis* (Lebanon), *Megabothris turbidus* (Turkey), *Myoxopsylla laverani* (Egypt, Israel), *Nosopsyllus baltazardi* (Iran), *Nosopsyllus consimilis* (Turkey), *Nosopsyllus durii* (Lebanon, Turkey), *Nosopsyllus fasciatus* (Cyprus, Egypt, Iran, Turkey), *Nosopsyllus geneatus* (Egypt), *Nosopsyllus gerbillophilus* (Egypt), *Nosopsyllus henleyi* (Egypt), *Nosopsyllus iranus* (Iran, Israel, Lebanon, Saudi Arabia), *Nosopsyllus laeviceps* (Iran), *Nosopsyllus londiniensis* (Egypt), *Nosopsyllus medus* (Iran), *Nosopsyllus penicus* (Egypt), *Nosopsyllus pringlei* (Iran), *Nosopsyllus pumilionis* (Israel), *Nosopsyllus sarinus* (Turkey), *Nosopsyllus sinaiensis* (Egypt), *Nosopsyllus* spp. (Egypt, Iran), *Nosopsyllus theodori* (Israel), *Nosopsyllus turkmenicus* (Iran), *Nosopsyllus vlasovi* (Iran), *Nosopsyllus ziarus* (Iran), *Paraceras melis* (Iran), *Paraceras* sp. (Iran) |
| --- |
| 2. **Family:** Coptopsyllidae  *Coptopsylla Africana* (Israel), *Coptopsylla bairamalienis* (Iran), *Coptopsylla iranica* (Iran), *Coptopsylla lamellifer* (Iran), *Coptopsylla mesghalii* (Iran), *Coptopsylla mofidii* (Iran), *Coptopsylla neronovi* (Iran) |
| 3. **Family:** Ctenophthalmidae  *Ctenophthalmus agyrtes* (Turkey), *Ctenophthalmus bifidatus* (Turkey), *Ctenophthalmus congener* (Lebanon), *Ctenophthalmus coniunctus* (Turkey), *Ctenophthalmus contiger* (Turkey), *Ctenophthalmus dolichus* (Iran), *Ctenophthalmus euxinicus* (Turkey), *Ctenophthalmus fissurus* (Turkey), *Ctenophthalmus fransmiti* (Turkey), *Ctenophthalmus golovi* (Turkey), *Ctenophthalmus harputus* (Turkey), *Ctenophthalmus hypanis* (Turkey), *Ctenophthalmus inornatus* (Turkey), *Ctenophthalmus iranus* (Iran), *Ctenophthalmus Proximus* (Turkey), *Ctenophthalmus reconditus* (Turkey), *Ctenophthalmus rettigi* (Iran), *Ctenophthalmus secundus* (Turkey), *Ctenophthalmus* sp. (Iran), *Ctenophthalmus stirps* (Turkey), *Ctenophthalmus teres* (Turkey), *Ctenophthalmus beyzanurae* (Turkey), *Ctenophthalmus kefelioglui* (Turkey), *Hystrichopsylla orientalis* (Turkey), *Palaeopsylla incisa* (Turkey), *Rhadinopsylla bivirgis* (Iran), *Rhadinopsylla masculana* (Egypt, Israel), *Rhadinopsylla pentacantha* (Turkey), *Rhadinopsylla syriaca* (Iran), *Rhadinopsylla ucrainica* (Iran), *Stenoponia tripectinata* (Egypt, Iran, Israel, Lebanon, Turkey), *Stenoponia vlasovi* (Iran) |
| 4. **Family:** Leptopsyllidae  *Amphipsylla rossica* (Iran, Turkey), *Amphipsylla* spp. (Iran), *Ctenophyllus rufescens* (Iran), *Frontopsylla elata* (Turkey), *Hopkinsipsylla occulta* (Egypt), *Leptopsylla aethiopicus* (Iran), *Leptopsylla algira* (Israel), *Leptopsylla segnis* (Cyprus, Egypt, Iran, Lebanon), *Leptopsylla taschenbergi* (Iran, Lebanon, Turkey), *Mesopsylla tuschkan* (Egypt), *Ophthalmopsylla volgensis* (Israel), *Paradoxopsyllus grenieri* (Iran), *Paradoxopsyllus microphthalmos* (Iran) |
| 5. **Family:** Pulicidae  *Ctenocephalides arabicus* (Egypt), *Ctenocephalides canis* (Cyprus, Egypt), *Ctenocephalides felis* (Cyprus, Egypt, Iran, Lebanon, Saudi Arabia), *Echidnophaga gallinacea* (Egypt), *Echidnophaga murina* (Egypt, Israel, Lebanon), *Echidnophaga oschanini* (Iran), *Parapulex chephrenis* (Egypt, Israel, Saudi Arabia, Yemen), *Pulex irritans* (Egypt, Iran, Israel, Turkey), *Synosternus pallidus* (Egypt), *Xenopsylla astia* (Egypt, Iran, Kuwait, Qatar), *Xenopsylla buxtoni* (Iran), *Xenopsylla cheopis* (Cyprus, Egypt, Iran, Israel, Lebanon, Palestine, Saudi Arabia, Yemen), *Xenopsylla cleopatrae* (Egypt, Iran, Israel, Saudi Arabia, Yemen), *Xenopsylla conformis* (Egypt, Iran, Israel, Saudi Arabia), *Xenopsylla dipodilli* (Egypt, Israel), *Xenopsylla gerbilli* (Iran), *Xenopsylla Hussain* (Iran), *Xenopsylla hutoni* (Iran), *Xenopsylla nubica* (Egypt, Iran, Saudi Arabia), *Xenopsylla nuttalli* (Iran), *Xenopsylla persica* (Iran), *Xenopsylla ramesis* (Egypt, Israel, Lebanon), *Xenopsylla* spp. (Egypt, Iran, Israel, Saudi Arabia, Yemen), *Xenopsylla taractes* (Egypt) |

## Supplementary Table S4c: Rodent lice reported in the Middle East

| 1. **Family:** Hoplopleuridae (suborder: Anoplura)  *Hoplopleura acanthopus* (Iran), *Hoplopleura affinis* (Iran), *Hoplopleura captiosa* (Egypt, Iran), *Hoplopleura longula* (Iran), *Hoplopleura meridionidis* (Iran), *Hoplopleura oenomydis* (Egypt), *Hoplopleura pacifica* (Egypt), *Hoplopleura* spp. (Iran) |
| --- |
| 2. **Family:** Pediculidae (suborder: Anoplura)  *Pediculus affinis* (Iran), *Pediculus humanus* (Egypt) |
| 3. **Family:** Polyplacidae (suborder: Anoplura)  *Eulinognathus aculeatus* (Iran), *Eulinognathus* sp. (Iran), *Neohaematopinus laeviusculus* (Iran), *Neohaematopinus* spp. (Iran), *Polyplax abyssinica* (Egypt), *Polyplax asiatica* (Iran), *Polyplax brachyrrhyncha* (Egypt, Iran), *Polyplax calomysci* (Iran), *Polyplax cannomydis* (Kuwait), *Polyplax gerbilli* (Iran, Israel), *Polyplax kaiseri* (Iran), *Polyplax oxyrrhyncha* (Egypt), *Polyplax paradoxa* (Iran), *Polyplax reclinate* (Iran), *Polyplax serrata* (Iran, Saudi Arabia), *Polyplax spinulosa* (Egypt, Iran, Kuwait, Palestine, Saudi Arabia), *Polyplax* spp. (Iran), *Polyplax stephensi* (Iran) |

## Supplementary Table S4d: Rodent mites reported in the Middle East

| 1. **Family:** Acaridae  *Tyrophagus* sp. (Egypt) |
| --- |
| 2. **Family:** Cheyletidae  *Cheyletus eruditus* (Egypt, Saudi Arabia), *Cheyletus* sp. (Egypt), *Cheyletus zaheri* (Egypt) |
| 3. **Family:** Demodicidae  *Demodex aurati* (Turkey), *Demodex criceti* (Turkey) |
| 4. **Family:** Dermanyssidae  *Dermanyssus americanus* (Iran), *Dermanyssus gallinae* (Egypt, Iran), *Dermanyssus muris* (Iran, Yemen), *Dermanyssus sanguineus* (Egypt, Iran), *Dermanyssus* spp. (Egypt, Iran) |
| 5. **Family:** Haemogamasidae  *Eulaelaps* spp. (Iran), *Eulaelaps stabularis* (Egypt, Iran, Turkey) |
| 6. **Family:** Hirstionyssidae  *Hirstionyssus arcuatus* (Turkey), *Hirstionyssus carticulatus* (Israel), *Hirstionyssus eversmani* (Turkey), *Hirstionyssus isabellinus* (Egypt, Turkey), *Hirstionyssus* spp. (Iran, Turkey) |
| 7. **Family:** Laelapidae  *Androlaelaps androgynus* (Israel), *Androlaelaps centrocarpus* (Israel), *Androlaelaps hermaphrodita* (Iran), *Androlaelaps hirsuta* (Israel), *Androlaelaps insculptus* (Israel), *Androlaelaps marshalli* (Israel), *Androlaelaps tateronis* (Saudi Arabia), *Articholaelaps glasgowi* (Saudi Arabia), *Echinolaelaps echidninus* (Egypt, Iran, Saudi Arabia), *Haemogamasus horridus* (Turkey), *Haemogamasus kusumotoi* (Turkey), *Haemogamasus nidiformis* (Turkey), *Haemogamasus pontiger* (Egypt), *Haemogamasus* sp. (Iran), *Haemogamasus zachvatkini* (Turkey), *Haemolaelaps androgynus* (Turkey), *Haemolaelaps fahrenholzi* (Turkey), *Haemolaelaps glasgowi* (Egypt, Iran, Turkey), *Haemolaelaps namrui* (Yemen), *Haemolaelaps* spp. (Egypt, Iran, Turkey), *Haemolaelaps zulu* (Egypt), *Hypoaspis astronomica* (Iran), *Hypoaspis koseii* (Egypt), *Hypoaspis miles* (Turkey), *Laelaps agilis* (Turkey), *Laelaps algericus* (Turkey), *Laelaps ciccuminata* (Iran), *Laelaps hilaris* (Turkey), *Laelaps jettmari* (Turkey), *Laelaps keegani* (Egypt), *Laelaps kochi* (Turkey), *Laelaps longisetosus* (Turkey), *Laelaps nuttalli* (Egypt, Iran, Kuwait, Saudi Arabia, Yemen)), *Laelaps pavlovskyi* (Turkey), *Laelaps sinai* (Egypt), *Laelaps* spp. (Iran), *Myonyssus decumani* (Turkey), *Myonyssus gigas* (Turkey), *Paracheylaelaps pyriformis* (Iran) |
| 8. **Family:** Leeuwenhoekiidae  *Multisetosa persicus* (Iran), *Shunsennia oudemansi* (Iran) |
| 9. **Family:** Listrophoridae  *Listrophorus arishi* (Egypt) |
| 10. **Family:** Macrochelidae  *Macrocheles muscaedomestica* (Turkey), *Macrocheles* spp. (Iran) |
| 11. **Family:** Macronyssidae  *Ornithonyssus bacoti* (Egypt, Iran, Kuwait, Saudi Arabia, Turkey), *Ornithonyssus* spp. (Egypt, Iran, Saudi Arabia), *Ornithonyssus sylviarum* (Egypt, Iran) |
| 12. **Family:** Myobiidae  *Myobia murismusculi* (Iran), *Myobia* sp. (Egypt),  *Radfordia acomys* (Iran), *Radfordia affinis* (Iran), *Radfordia dyromys* (Iran), *Radfordia ensifera* (Egypt), *Radfordia merioni* (Iran), *Radfordia persica* (Iran), *Radfordia* sp. (Egypt) |
| 13. **Family:** Myocoptidae  *Myocoptes musculinus* (Iran), *Trichoecius calomysci* (Iran), *Trichoecius* sp. (Egypt) |
| 14. **Family:** Oribatulidae  Zygoribatula sp. (Egypt) |
| 15. **Family:** Trombiculidae  *Ascoschoengastia browni* (Saudi Arabia), *Brunehaldia iranica* (Iran), *Brunehaldia lucida* (Iran), *Brunehaldia sylvatica* (Iran), *Cheladonta afshari* (Iran), *Cheladonta firdousii* (Iran), *Cheladonta iraniensis* (Iran), *Doloisia skljari* (Iran), *Ericotrombidium caucasicum* (Saudi Arabia), *Ericotrombidium galliardi* (Saudi Arabia), *Ericotrombidium kazeruni* (Saudi Arabia), *Euschoengastia meshhedensis* (Iran), *Helenicula lukshumiae* (Saudi Arabia), *Helenicula sparsa* (Iran), *Hirsutiella alpine* (Iran),  *Kepkatrombicula brevis*  (Iran), *Kepkatrombicula magna* (Iran), *Leptotrombidium noxium* (Iran), *Leptotrombidium raropinne* (Iran), *Leptotrombidium silvaticum* (Iran), *Leptotrombidium subsilvaticum* (Iran), *Microtrombicula centropi* (Saudi Arabia), *Microtrombicula hoogstraali* (Saudi Arabia, Yemen), *Microtrombicula hyraci* (Saudi Arabia), *Microtrombicula microscuta* (Saudi Arabia), *Microtrombicula muhaylensis* (Saudi Arabia), *Microtrombicula traubi* (Iran, Saudi Arabia), *Miyatrombicula ramitensis* (Iran), *Neoschongastia yemenensis* (Yemen), *Neotrombicula aideriensis* (Iran), *Neotrombicula autumnalis* (Iran), *Neotrombicula delijani* (Iran), *Neotrombicula elegans* (Iran), *Neotrombicula faghihi* (Turkey), *Neotrombicula lazistanica* (Turkey), *Neotrombicula lubrica* (Iran), *Neotrombicula monticola* (Iran), *Neotrombicula rostrata* (Iran), *Neotrombicula sabzavari* (Turkey), *Neotrombicula saperoi* (Yemen), *Neotrombicula subtilis* (Turkey), *Neotrombicula talmiensis* (Iran), *Neotrombicula tehranensis* (Iran), *Neotrombicula turkestanica* (Iran), *Neotrombicula vernalis* (Iran), *Neotrombicula vulgaris* (Iran), *Pentidionis agamae* (Saudi Arabia), *Walchia cognata* (Iran), *Walchia parvula* (Saudi Arabia) |
| 16. **Family:** Walchiidae  *Gahrliepia lawrencei* (Saudi Arabia), *Schoengastiella wansoni* (Saudi Arabia), *Schoutedenichia angusta* (Iran), *Schoutedenichia asirensis* (Saudi Arabia), *Schoutedenichia Saudi* (Saudi Arabia), *Schoutedenichia thracica* (Saudi Arabia), *Schoutedenichia zarudnyi* (Saudi Arabia) |

## Supplementary table S4e: Rodent ticks reported in the Middle East

| 1. **Family:** Argasidae (Soft tick)  Ornithodoros sp. (Iran) |
| --- |
| 2. **Family:** Ixodidae (Hard tick)  *Amblyomma* sp. (Egypt), *Boophilus annulatus* (Iran), *Boophilus* sp. (Iran), *Dermacentor marginatus* (Turkey), *Haemophysalis leachi* (Yemen), *Haemaphysalis punctata* (Iran), *Haemaphysalis* spp. (Iran, Israel, Turkey, Yemen), *Hyalomma asiaticum* (Iran), *Hyalomma dromedarii* (Egypt), *Hyalomma excavatum* (Egypt), *Hyalomma impeltatum* (Israel, Saudi Arabia), *Hyalomma Koch* (Iran), *Hyalomma rhipicephaloides* (Egypt), *Hyalomma savignyi* (Israel), *Hyalomma* spp. (Egypt, Iran, Saudi Arabia), *Ixodes eldaricus* (Israel), *Ixodes laguri* (Turkey), *Ixodes redikorzevi* (Iran, Turkey), *Ixodes* spp. (Iran, Turkey, Yemen), *Ixodes trianguliceps* (Iran), *Rhipicephalus bursa* (Iran), *Rhipicephalus camicasi* (Saudi Arabia), *Rhipicephalus sanguineus* (Egypt, Israel, Saudi Arabia), *Rhipicephalus simus* (Yemen), *Rhipicephalus* spp. (Egypt, Iran, Saudi Arabia, Yemen), *Rhipicephalus turanicus* (Iran, Israel, Saudi Arabia |
